# Small extracellular vesicle signaling and mitochondrial transfer reprogram T helper cell function in human asthma

**DOI:** 10.1038/s41467-026-73684-y

**Published:** 2026-05-26

**Authors:** Kenneth P. Hough, Jennifer L. Trevor, Shaheer Ahmad, Yong Wang, Balu K. Chacko, Kayla F. Goliwas, John G. Strenkowski, Yuelong Liu, Joanna I. Nowak, Eugene J. Becker Jr, Young-il Kim, Renita Holmes, Nathaniel B. Bone, Shia Vang, Alexandra Pritchard, Jay Chin, Sandeep Bodduluri, Veena B. Antony, Sultan Tousif, Mohammad Athar, Diptiman Chanda, Kasturi Mitra, Jaroslaw W. Zmijewski, Jianhua Zhang, Steven R. Duncan, Victor J. Thannickal, Susanne Gabrielsson, Victor M. Darley-Usmar, Jessy S. Deshane

**Affiliations:** 1 Division of Pulmonary, Allergy, and Critical Care Medicine, Department of Medicine, University of Alabama at Birmingham, Birmingham, AL USA; 2 Mitochondrial Medicine Laboratory, Department of Pathology, University of Alabama at Birmingham, Birmingham, AL USA; 3 Department of Histology, Medical University of Gdansk, Gdansk, Poland; 4 Division of Global and Rural Health, Department of Obstetrics and Gynecology, University of Alabama at Birmingham, Birmingham, AL USA; 5 Department of Cellular Biology & Anatomy, LSUHS, LSU Health, Shreveport, LA USA; 6 Department of Dermatology, University of Alabama at Birmingham, Birmingham, AL USA; 7 Department of Genetics, University of Alabama at Birmingham, Birmingham, AL USA; 8 Department of Biology, Ashoka University, National Capital Region-Delhi, India; 9 Department of Medicine, Tulane University School of Medicine and the Southeast Louisiana Veterans Health Care System, New Orleans, LA USA; 10 Division of Immunology and Respiratory Medicine, Department of Medicine, Karolinska Institutet, Stockholm, Sweden; 11 Clinical Immunology and Transfusion Medicine and Center for Molecular Medicine, The Karolinska University Hospital, Stockholm, Sweden

**Keywords:** Asthma, Chronic inflammation, Mucosal immunology

## Abstract

Small extracellular vesicles (sEVs) orchestrate cell-cell communication, but the role of sEV signaling via mitochondria in perpetuating asthmatic airway inflammation is unknown. Myeloid-derived regulatory cells (MDRCs) control CD4^+^ T cell responses in asthma. We demonstrate that airway MDRC-derived sEVs from asthmatics mediate T cell receptor engagement and transfer of mitochondria that induce antigen-specific activation and polarization of Th17 and Th2 cells. sEV-dependent T cell activation and Th polarization were mediated by mitochondrial oxidant-dependent NF-κB signaling, which, when blocked, mitigated CD4^+^ T cell activation. Mitochondrial fission regulator, DRP-1, promoted mitochondrial packaging within MDRC-sEVs. Internalized sEVs co-localized with the polarized cytoskeleton and mitochondrial networks in recipient T cells. Intranasal transfer of mitochondria packaged sEVs enhanced allergic airway inflammation and Th polarization in a murine asthma model. Our studies indicate a previously unrecognized role for mitochondrial fission and sEV- mitochondria-mediated signaling in dysregulated T cell activation, Th polarization, and pathology in asthma.

## Abstract (web summary)

The mechanisms for unremitting airway inflammation in human asthma remain to be explored. The authors here identify that mitochondrial-small extracellular vesicle-mediated signaling in T cell activation and differentiation contributes to dysregulated immune responses in asthma.

## Introduction

CD4^+^ T cells orchestrate the immune response in diverse chronic inflammatory diseases, including asthma^1^. Pathogenic T helper type 2 (Th2) and T helper type 17 (Th17) subsets drive airway inflammation, remodeling, and smooth muscle hyperplasia in asthmatics^2–11^. Recent advances implicate cellular crosstalk between the innate and adaptive immune systems in the initiation and propagation of the allergic immune response. In allergic asthma, antigen-presenting cells (APCs) internalize antigens, present processed peptides bound to class II molecules^12–14^ and engage the T cell receptor (TCR), triggering a multi-molecular signaling cascade to activate CD4^+^ T cells^15^. HLA-DR-expressing APCs, including immature myeloid cells, regulate T cell function through TCR engagement^16–21^. In human asthma, the pro-inflammatory HLA-DR^+^ subsets of myeloid-derived regulatory cells (MDRCs) promote proliferation of T cells via redox signaling pathways^21^. Although antigen presentation by MDRCs is not well-defined, TCR engagement and redox mechanisms may synergize to regulate MDRC-mediated T cell activation.

Recently, acellular antigen presentation by exosomes, has been described as an unconventional mechanism of immune activation^22–25^. Small extracellular vesicles (sEVs), an endosomally-derived class of extracellular vesicles, that are known for facilitating cellular crosstalk, are decorated with scaffolding proteins for immune receptors called tetraspanins^24,26–31^. Tetraspanins cluster at the immune synapse, and aid in antigen presentation and immune signaling^30^. Thus, sEVs may modulate the immune response via the immune synapse. While professional APC-derived MHC-peptide-bound sEVs induce Th2 cytokines in experimental asthma^22,24,32^, antigen presentation by MDRC-derived sEVs as a driver of dysregulated CD4^+^ T cell responses and Th differentiation has not been demonstrated.

We reported that MDRC-derived sEVs containing polarized mitochondria are internalized by CD4^+^ T cells^33^. Increasing evidence for mitochondrial components and respiration in sEVs suggests that functional mitochondria packaged within the sEVs may elicit diverse physiologic responses^33–39^. This unconventional signaling mechanism may compensate for the altered bioenergetics of recipient cells and/or provide an alternative mechanism to outsource autophagy/mitophagy during cellular stress^36,37,40,41^.

Here, we show that MDRC-derived sEV mitochondria with intact membrane potential activate CD4^+^ T cell proliferation and Th polarization into Th2 and Th17 cells via mitochondrial oxidant-mediated NF-κB signaling; this event is triggered by membrane fusion of sEV-derived mitochondria and downstream complex formation with polarized cytoskeleton and mitochondrial network in recipient T cells. We demonstrate that DRP1 promotes packaging of functional mitochondria within sEVs and implicates MDRC-derived sEV-dependent mitochondrial signaling in enhancing in vivo Th cell polarization, chronic airway inflammation, and asthma pathology in mice with asthma and in human asthmatics. Our studies suggest targeting sEV-mitochondrial signaling to attenuate the Th cell programming underlying allergic asthma responses.

## Results

### Bronchoalveolar lavage fluid (BALF) sEVs from asthmatics drive CD4^+^ T cell proliferation and Th polarization

We determined the concentration and size distribution of human BALF EVs (Fig. S1) from healthy controls and asthmatics (Table 1: Characteristics of study subjects). BALF EVs from both asthmatic and healthy individuals were predominantly 65-150 nm with a median size consistent with sEVs, and asthmatics had a slightly higher number of sEVs compared to healthy individuals (Fig. S1A–D). Immune regulation by sEVs is attributed to expression of MHC-II (HLA-DR) and co-stimulatory molecules CD81, CD86, and CD54^25,42^. We and others reported that the % HLA-DR^+^ EVs and HLA-DR expression are increased in airways of asthmatics compared to healthy controls^24,33,43,44^. Additionally, we showed that both human BALF and airway MDRC-derived sEVs were MitoTracker Green^+^(MitoTG^+^) and transferred mitochondria to T cells^33^. Consistent with this, we saw here that BALF sEVs that were CD63^+^ and CD63^+^MitoTG^+^ were HLA-DR^+^ and were increased in asthmatics; sEVs from both healthy controls and asthmatics expressed HLA-DR (Fig. S1E–J). Nanoimaging studies on BALF sEVs confirmed their size (Fig. S2A), EV marker distribution (Fig. S2B), and presence of Tomm20^+^ (mitochondrial outermembrane protein) BALF sEVs in both study groups (Fig. S2B); high-resolution imaging confirmed expression of this mitochondrial marker in BALF sEVs (Fig. S2C). Expression of CD81 (sEV marker) as well as TIM23 (an inner mitochondrial membrane protein) was further confirmed (Fig. S2D) by western blot analyses.

**Table 1:** Characteristics of volunteer subjects enrolled in the study

Characteristics of enrolled patients				
Characteristics		Healthy (*N* = 22)	Asthmatics (*N* = 21)	*p*-value
Sex				
Male		8	4	
Female		14	17	
Median age (IQR)		44 (23)	40 (26)	0.3054
Median IgE titer (IQR)—IU/L		24.71 (83.2)	102.3 (556.2)	0.0254
Median predicted % FEV1 (IQR)		106.3 (20.14)	85 (30)	0.0001
Median eosinophils (IQR)		123.6 (86.8)	227 (146.2)	0.0029
Median neutrophils (IQR)		3.71 (1.61)	3.59 (1.82)	0.5371

**Fig. 1:**
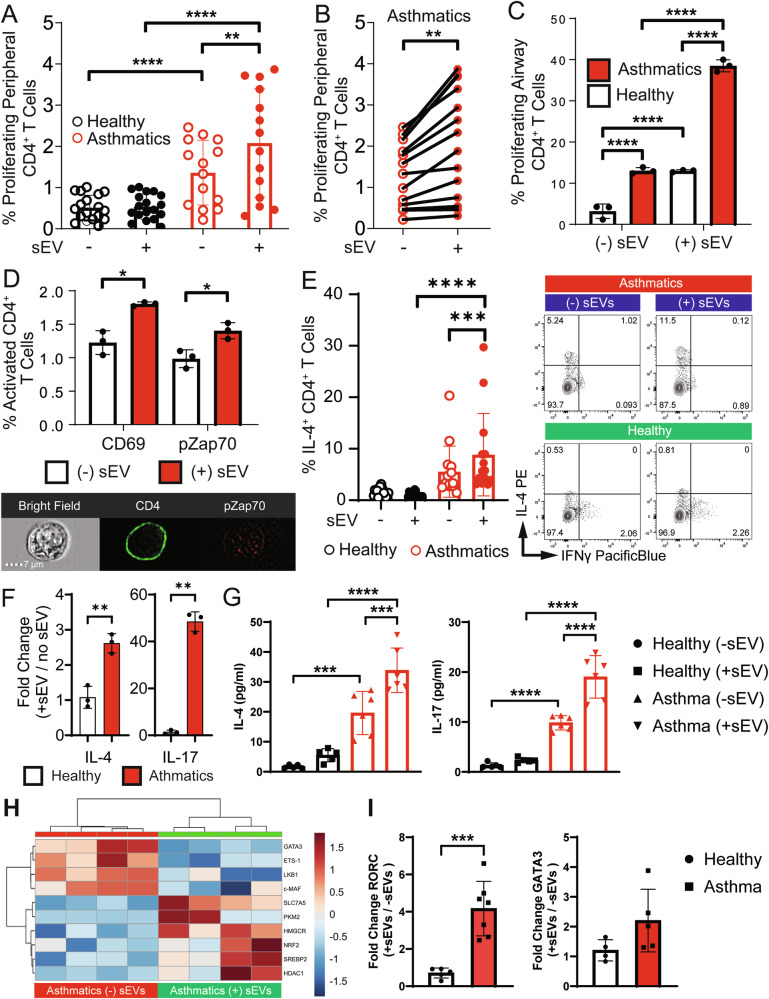
BALF sEVs from the airways of asthmatics promote proliferation and activation of CD4^+^ T cells, alter transcriptional signature, and enhance T helper cell polarization. **A**, **B** Percentage of proliferating peripheral CD4^+^ T cells after co-culture with airway sEVs. sEVs from the BAL fluid (BALF) were co-cultured with CFSE-labeled autologous peripheral CD4^+^ T cells at a ratio of 1:10 (T cells:sEVs) in the presence of rhIL-2 (50 IU/ml) for 7 days. Cells were harvested at day 7 for flow cytometry analysis on the BD LSRII. **A** Mixed Effect ANOVA with Sidak’s multiple comparisons test was performed (*n* = 14–18), ^**^p = 0.0013, ^****^p < 0.0001. **B** Mann–Whitney *T*-test for comparison between no sEVs vs sEVs in asthmatics, ^**^p < 0.01. **C** Percentage of proliferating airway CD4^+^ T cells after co-culture with airway sEVs. sEVs from the BALF were co-cultured as described above with autologous airway CD4^+^ T cells. Cells were harvested at day 7 for flow cytometry analysis on the BD LSRII. Mixed Effect ANOVA with Sidak’s multiple comparison test, ^****^p < 0.0001 (*n* = 3). **D** BALF sEVs were co-cultured with autologous CD4^+^ T cells (1:10 T cells:sEVs) for 15 min, and the percent CD69^+^ or phospho-Zap70^+^ was assessed by ImageStream imaging flow cytometry (representative image). Mixed Effect ANOVA with Sidak’s multiple comparisons test (*n* = 3), ^*^p < 0.05. **E** sEVs purified from the BALF were co-cultured with autologous peripheral CD4^+^ T cells in a ratio of 1:10 (T cells: sEVs) in the presence of rhIL-2 (50 IU/ml) for 7 days. Cells were harvested at day 7 for intracellular staining of cytokines and analyzed by flow cytometry using a BD LSRII. Left, percent of Th2 cells (assessed by IL-4 expression), and representative flow plots shown on the right. Mixed Effect ANOVA with Sidak’s multiple comparisons test (*n* = 14–17), ^***^p = 0.0008, ^****^p < 0.0001. **F** Quantitative real-time PCR analysis of IL-4 and IL-17 gene expression of healthy and asthmatic CD4^+^ T cells co-cultured with and without sEVs for 7 days. Data are normalized to gene expression in T cells cultured without sEVs (ACTB as reference gene). Co-culture of asthmatic sEVs with T cells significantly increased IL-4 and IL-17 gene expression (*n* = 3), Mann–Whitney *T*-test ^**^p < 0.01, ^****^p < 0.0001. **G** Cytokine measurements by ELISA of IL-4 and IL-17 in co-culture supernatants collected from CD4^+^ T cells co-cultured with autologous BALF sEVs at Day 7 of culture (*n* = 6/study group/experimental group, 3 replicates each), One way ANOVA (*n* = 5 for Healthy, *n* = 6 for Asthma, Mean of two replicate values shown for each sample), ^**^p = 0.0016, ^***^p = 0.0003 and ^****^p < 0.0001 for IL-4, ^****^p < 0.0001 for IL-17, **H** Clustered heatmap generated using MetaboAnalyst 3.0 from a custom NanoString panel comparing gene expression of CD4^+^ T cells from asthmatics co-cultured with BALF sEVs (right), and no sEVs control (left). Heatmap and clustering were generated using the nSolver Analysis Software 4.0 from nanoString (*n* = 3). **I** Quantitative real-time PCR analysis of RORC and GATA-3 gene expression of healthy and asthmatic CD4^+^ T cells co-cultured with and without sEVs. Data are normalized to gene expression in T cells cultured without sEVs. Mann–Whitney *t*-test for comparison between healthy and asthmatic subjects (*n* = 4 for healthy, *n* = 7 for asthma). Individual data points are presented with each bar representing mean ± SD. Source data are provided as a Source Data file.

BALF sEVs when co-cultured with peripheral autologous CD4^+^ T cells (10 sEVs:1T cell ratio), induced their proliferation (CFSE dilution) (Figs. 1A, B, and S3A). Furthermore, proliferation of autologous airway CD4^+^ T cells of asthmatics was increased in co-cultures with BALF sEVs (Figs. 1C and S3B). An increase in %CD69^+^ T cells (an early activation marker), and an increase in phosphorylated Zap70 (pZap70), a kinase downstream of the TCR (Fig. 1D), was observed, suggesting an MHC Class II-TCR interaction.

Additionally, airway sEVs from asthmatics modulated polarization of autologous CD4^+^ T cells into Th subsets. The baseline Th2 response in asthmatics compared to healthy controls was enhanced following co-culture with BALF sEVs (Figs. 1E and S4). Th17 polarization was also elevated in asthmatics with an increase in % IL-17^+^ T cells (Figs. S3C and S4) and an increase in fold change of both IL-4 and IL-17 gene expression (Fig. 1F) and IL-4 and IL-17 cytokine production (Fig. 1G). Modulation of transcription factors, metabolic genes, and epigenetic modifiers underlie Th polarization^45,46^. Principal component analyses of gene expression in T cells from asthmatics co-cultured with or without sEVs indicated unique differences in gene programs (Fig. S3D). Heatmaps with hierarchical clustering revealed differential modulation of metabolic genes, transcription factors, and cytokines (Fig. S3E). The top ten differentially expressed genes included sterol pathway genes (*SREBP2, HMGCR*), the glutamate transporter (*SLC7A5*), hexokinase 2 (*HK2*), Phosphoglycerate kinase 1 (*PGK1*) and histone deacetylase 1 (*HDAC1)*, in line with Th17 gene programming (Figs. 1H and S3E); *RORC*, the gene encoding the retinoic acid receptor-related orphan receptor gamma t (RORγt), a transcription factor for Th17 cell development and function, was increased in the asthma group (Fig. 1I). The difference in GATA3 expression in these co-cultures from asthmatics was not significant (Fig. 1I). Thus, airway sEVs from asthmatics enhance T cell proliferation, activation, and pathogenic Th polarization.

### MDRC-derived sEVs containing mitochondria are internalized by T cells via membrane fusion

We have previously reported that the pro-inflammatory airway MDRC-derived sEVs containing mitochondria are internalized by peripheral autologous CD4^+^ T cells^33^. Although treatment of MDRCs with mitochondrial complex I inhibitor (rotenone) and uncoupler (FCCP) did not modulate the % MitoTG^+^ sEVs, evaluation with a mitochondrial membrane potential dye, tetramethylrhodamine methyl ester (TMRE), showed that TMRE^+^MitoTG^+^ BALF sEVs were present only in asthmatics (Fig. S5A–C). Treatment of sEVs with FCCP or rotenone reduced the % TMRE^+^MitoTG^+^CD63^+^ sEVs, suggesting that the airways of asthmatics have an increased proportion of sEVs that maintain mitochondrial membrane potential (Fig. S5B). Transduction of purified pro-inflammatory airway MDRCs with baculovirus constructs CellLight Mitochondria-GFP (Mito-GFP)^33^ yielded MDRC-derived GFP^+^ sEVs, which were also internalized by T cells (Fig. S5D).

**Fig. 2:**
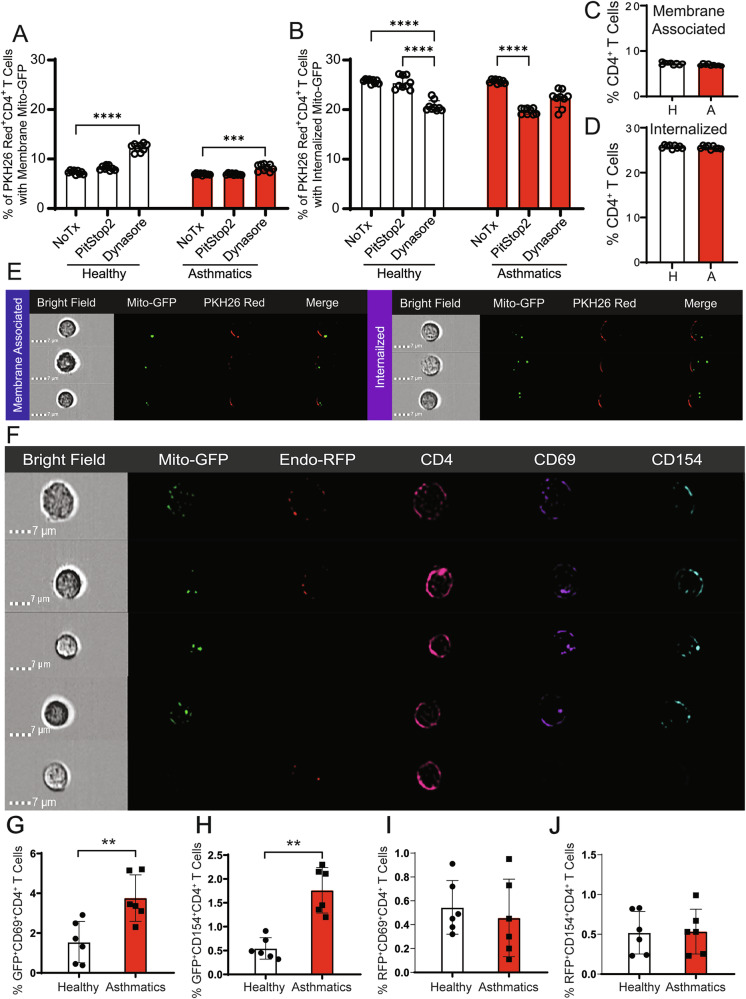
CD4^+^ T cells internalize MDRC-derived sEVs predominantly by membrane fusion and not by endocytosis. Activation of CD4^+^ T cells is associated with internalization of Mito-GFP^+^ sEVs derived from airway MDRCs. **A**, **B** Blockade of clathrin-dependent and independent mechanisms of endocytosis did not completely abrogate MDRC-derived Mito-GFP^+^ sEV internalization by CD4^+^ T cells. CD4^+^ T cells were treated with PitStop2 (50 nM), Dynasore (50 μM), or a combination of the two prior to co-culture with MDRC-derived sEVs for 24 h, and internalization was assessed by ImageStream flow cytometry. **A** Quantitation of the number of CD4^+^ T cells with membrane-associated Mito-GFP^+^ MDRC sEVs. **B** Quantitation of the number of CD4^+^ T cells with internalized Mito-GFP^+^ MDRC sEVs. **A**, **B** Mixed Effect ANOVA with Sidak’s multiple comparisons test (n = 9 each for Healthy and Asthma), ^***^<0.001, ^****^<0.0001. **C**–**E** MDRCs were transduced with Mito-GFP, and the membrane was labeled with PKH26 red. sEVs derived from MDRCs were purified 48 h later. Purified MDRC-derived sEVs were co-cultured with autologous peripheral CD4^+^ T cells for 24 h in the presence of rhIL-2 (50 IU/ml) in a ratio of 1:10 T cells:sEVs. Association of sEVs with the membrane or intracellular localization of sEVs was assessed by ImageStream flow cytometry. **C** Quantitation of membrane-associated Mito-GFP^+^ MDRC sEVs (*n* = 9 each for Healthy and Asthma). **D** Quantitation of intracellular Mito-GFP^+^ MDRC sEVs (*n* = 9 each for Healthy and Asthma). **E** Representative image strips from ImageStream flow cytometry analysis illustrating the association of PKH26 red with the membrane and intracellular space or membrane association of Mito-GFP. **F**–**J** MDRCs were transduced with CellLight Mito-GFP and CellLight Endo-RFP, and sEVs were purified from the supernatant 48 h later. Autologous peripheral human T cells were cultured with MDRC sEVs for 24 h, and internalization and activation were assessed by ImageStream imaging flow cytometry. An sEV -generator-cell (MDRCs) to T cell ratio of 2.5 × 10^5^:1 × 10^6^ was maintained. **F** Representative images from ImageStream illustrating MDRC sEV internalization and T cell activation (CD69 and CD154). **G**, **H** Percent of GFP^+^CD69^+^CD4^+^ and GFP^+^CD154^+^CD4^+^ T cells that represent T cells that have internalized GFP^+^ sEVs and show early activation and antigen-specific activation. **I**, **J** Percent of RFP^+^CD69^+^CD4^+^ and RFP^+^CD154^+^CD4^+^ T cells that represent T cells that have internalized RFP^+^ sEVs and show early activation and antigen-specific activation (**F**, **G**) Mann–Whitney *T* (*n* = 6 each for Healthy and Asthmatics), ^**^<0.01. Individual data points are presented with each bar representing mean ± SD. Source data are provided as a Source Data file.

We next investigated the mechanism of sEV uptake. Since clathrin and dynamin-dependent endocytosis pathways or micropinocytosis, facilitate internalization of sEVs by immune and non-immune cells^47,48^, we examined if their inhibition alters internalization of Mito-GFP^+^ sEVs by T cells. Although T cells are non-phagocytic, surface receptor recycling in CD4^+^ T cells is clathrin-dependent^49^. Treatment of CD4^+^ T cells with PitStop2 or Dynasore (inhibitors of clathrin and dynamin) decreased membrane- and cytoplasm-associated Mito-GFP^+^ sEVs only modestly (Fig. 2A, B), suggesting that endocytosis is not the primary mechanism for sEV internalization. To assess if sEV membrane fused with recipient T cell membranes, we pre-labeled Mito-GFP^+^ sEV with PKH26; in co-cultures, PKH26 signal remained restricted to the T cell membrane while the GFP signal was within the CD4^+^ T cells (Fig. 2E). The percent of CD4^+^ T cells with membrane-associated and internalized signals was not different between study groups (Fig. 2C, D). Thus, membrane fusion may be the primary mode of internalization of sEVs in lymphocytes.

### Mito-GFP^+^ MDRC-derived sEVs are sufficient to trigger CD4^+^ T cell activation

To confirm endosomal derivation of Mito-GFP^+^ sEVs, we transduced MDRCs with baculovirus constructs Mito-GFP and CellLight Early Endosome-RFP (Endo-RFP). We observed two distinct populations of either Mito-GFP^+^ or Endo-RFP^+^ sEVs. The expression of CD81 and CD63 (Fig. S5E) and % CD63^+^, CD81^+^, or CD63^+^CD81^+^ sEVs were not different between Mito-GFP^+^ and Endo-RFP^+^ sEVs. Importantly, GFP and RFP signals did not overlap within sEVs, or co-localize following internalization in T cells (Fig. 2F). Therefore, the Mito-GFP^+^ sEVs may have an alternate pathway of biogenesis than the Endo-RFP^+^ sEVs, and/or they follow different paths following internalization by T cells. We investigated whether internalized Mito-GFP^+^ sEVs elicit activation of co-cultured autologous peripheral CD4^+^ T cells, by assessing % CD69^+^ (early activation marker) and CD154^+^ (marker for antigen-specific activation) T cells. Only CD4^+^ T cells that internalized Mito-GFP^+^ or both Endo-RFP^+^ and Mito-GFP^+^ sEVs demonstrated an increase in both early and antigen-specific activation signals (Fig. 2F–H). Furthermore, activation was only seen in T cells of asthmatics and not in the healthy controls or in T cells that internalized only Endo-RFP^+^ sEVs (Fig. 2I, J). These data suggest that internalization of the Mito-GFP^+^ sEVs was sufficient for promoting T cell activation in asthmatics.

### Inhibition of MHC-II blocks MDRC-derived Mito-GFP^+^ sEV-mediated activation

**Fig. 3:**
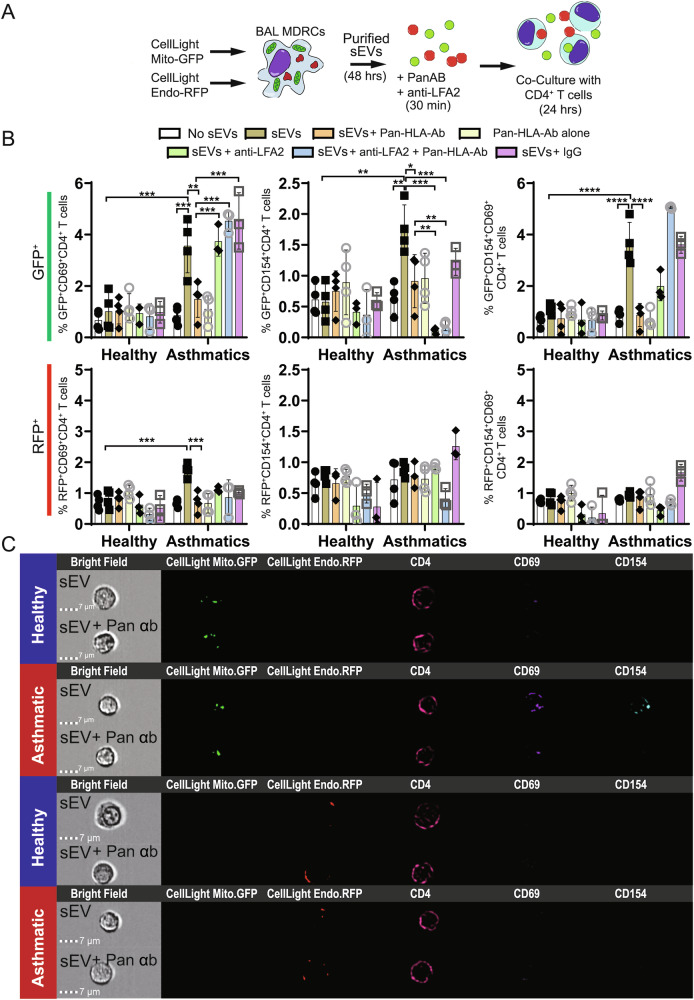
Blockade of class II molecules using a pan-HLA antibody (HLA-DR/DP/DQ) results in loss of MDRC sEV mediated activation of CD4^+^ T cells. **A** Illustration of an experimental approach. **B**, **C** MDRCs were transduced with CellLight Mito-GFP and CellLight Endo-RFP, and sEVs were purified from the supernatant 48 h later. sEVs derived from MDRCs transduced with Mito-GFP and Endo-RFP were co-cultured with autologous peripheral CD4^+^ T cells for 24 h in the presence of rhIL-2 (50 IU/ml) in a ratio of 1:10 T cells:sEVs. MDRC sEVs were pre-treated with pan-HLA-Ab (10 µg/ml), LFA2 antibody (1 µg/ml), IgG control, and a combination of pan-HLA-Ab and anti-LFA2 for 30 min at room temperature. sEVs were washed and re-purified using the Invitrogen Total Exosome Isolation kit. ImageStream flow cytometry was used to assess early activation (CD69) and antigen-specific activation (CD154) marker expression. **B** Graphs illustrating the percentage of CD69^+^, CD154^+^, or CD69^+^CD154^+^ T cells that internalized either Mito-GFP^+^ MDRC sEVs (top row) or Endo-RFP^+^ MDRC sEVs (bottom row). Two-way ANOVA, Tukey’s multiple comparisons test, ^*^p < 0.05, ^**^p < 0.01, ^***^p < 0.001, ^****^p < 0.0001. (*n* = 4 each for Healthy and Asthmatics, Mean of three replicates for each sample is represented). **C** Representative image strips from ImageStream illustrating that Mito-GFP^+^ MDRC sEVs activate T cells in asthmatics, while treatment with pan-HLA-Ab abrogates this activation by MDRC sEVs. Individual data points are presented with each bar representing the mean ± SD. Source data are provided as a Source Data file.

We determined if MDRC-derived sEVs activate CD4^+^ T cells through MHC-II-TCR interaction (Fig. 3A). When MDRC-sEVs were pre-treated with a pan-HLA antibody (targets HLA-DR, HLA-DP, and HLA-DQ), and then co-cultured with autologous peripheral CD4^+^ T cells, the activation of T cells that had internalized Mito-GFP^+^ (Fig. 3B, C) or both Mito-GFP^+^ and Endo-RFP^+^ sEVs (Fig. S6A–D), was inhibited (Fig. 3B, C) in asthmatics. The internalization of sEVs was not abrogated (Fig. 3C), suggesting activation was independent of internalization. When MDRC-derived sEVs were pre-treated with LFA-1 antibody (blocks LFA-1-ICAM1 interaction), differences in early activation (CD69^+^) were not observed (Fig. 3B); however, antigen-specific activation (CD154^+^) and CD69^+^CD154^+^ populations were reduced in asthmatics compared to sEV treatment alone.

### Inhibition of mitochondrial complex III and V abrogates MDRC-derived Mito-GFP^+^ sEV-mediated CD4^+^ T cell activation

**Fig. 4:**
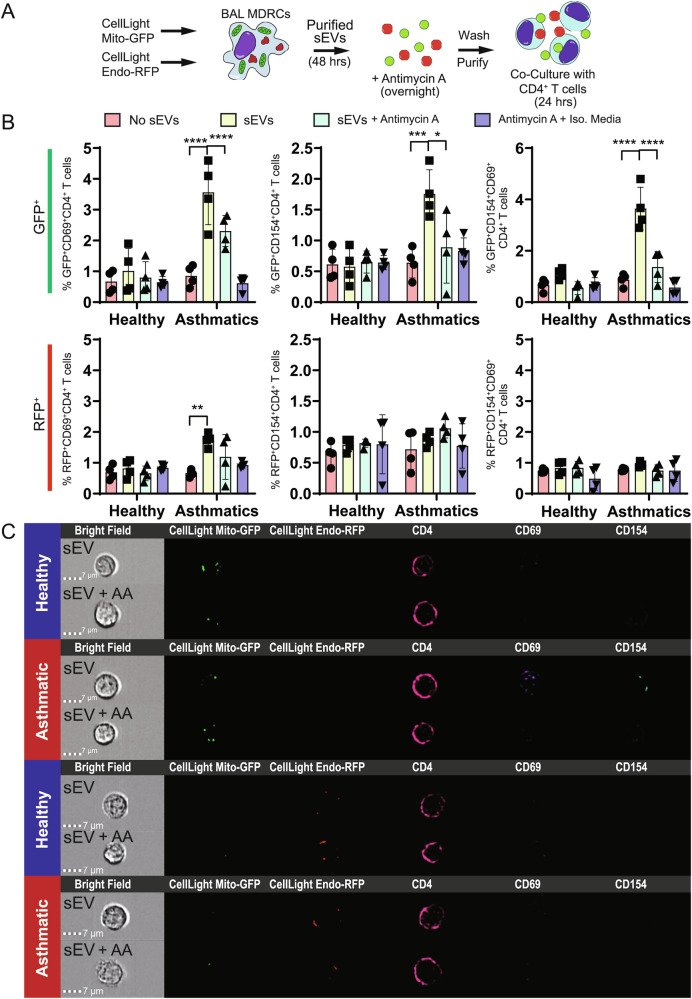
Inhibition of complex III by antimycin A in MDRC sEVs diminishes CD4^+^ T cell activation in asthmatics. **A** Illustration of an experimental approach. **B**, **C** MDRCs were transduced with CellLight Mito-GFP and CellLight Endo-RFP, and sEVs were purified from the supernatant 48 h later. sEVs derived from MDRCs transduced with Mito-GFP and Endo-RFP were pre-treated with antimycin A (10 µM) overnight or untreated prior to co-culture with autologous peripheral CD4^+^ T cells for 24 h in the presence of rhIL-2 (50 IU/ml) in a ratio of 1:10 T cells:sEVs. sEVs were washed and re-purified using the Invitrogen Total Exosome Isolation kit. ImageStream flow cytometry was used to assess early activation (CD69) and antigen-specific activation (CD154). **B** Graphs illustrating the percentage of CD69^+^, CD154^+^, or CD69^+^CD154^+^ T cells that internalized either Mito-GFP^+^ MDRC sEVs (top row) or Endo-RFP^+^ MDRC sEVs (bottom row). Two-way ANOVA, Tukey’s multiple comparisons test (*n* = 4 each for Healthy and Asthmatics, Mean of three replicates for each sample is represented), ^**^p < 0.01, ^***^p < 0.001, ^****^p < 0.0001. **C** Representative image strips from ImageStream analysis illustrating that Mito-GFP^+^ MDRC sEVs activate T cells in asthmatics, and antimycin A blocks activation in asthmatics. Individual data points are presented with each bar representing the mean ± SD. Source data are provided as a Source Data file.

We then determined if the mitochondria in Mito-GFP^+^ sEVs have functional effects on CD4^+^ T cell activation. Pre-treatment of MDRC-derived Mito-GFP^+^ sEVs with antimycin A, a high-affinity complex III inhibitor (Fig. 4A), before co-culture with T cells abrogated Mito-GFP^+^ sEV-mediated CD4^+^ T cell activation (Figs. 4B, C and S6C, D). To control for potential transfer of mitochondrial inhibitors from the sEV preparation to recipient cells, an sEV-free isolation medium with inhibitors was included, which had no effect (Figs. 4B, C and S6C, D). To determine if the inhibition was dependent on ATP synthesis by sEV- mitochondria, we pre-treated MDRC-derived sEV with oligomycin, a mitochondrial ATP synthase inhibitor. Oligomycin pre-treatment abrogated Mito-GFP^+^ sEV-mediated activation of CD4^+^ T cells (Fig. S7A–D), consistent with a requirement of a functional oxidative phosphorylation system within the sEV mitochondria to activate T cells.

Interestingly, pre-treatment of MDRC-derived sEVs with the complex I inhibitor, rotenone, enhanced Mito-GFP^+^ sEV-mediated activation of peripheral CD4^+^ T cells in asthmatics and, importantly, induced activation of healthy T cells (Fig. S8A–D). This aberrant activation of CD4^+^ T cells, short-circuiting the conventional MHC-II-TCR activation pathways, is unlikely to be solely due to effects on oxidative phosphorylation since oligomycin, antimycin A, and rotenone inhibit mitochondrial ATP synthesis but differ in their mechanisms. Oligomycin, rotenone, and Antimycin generate ROS through different mechanisms and sites in the mitochondria^50,51^.

### Activation of CD4^+^ T cells requires ROS generated within MDRC-derived sEVs

**Fig. 5:**
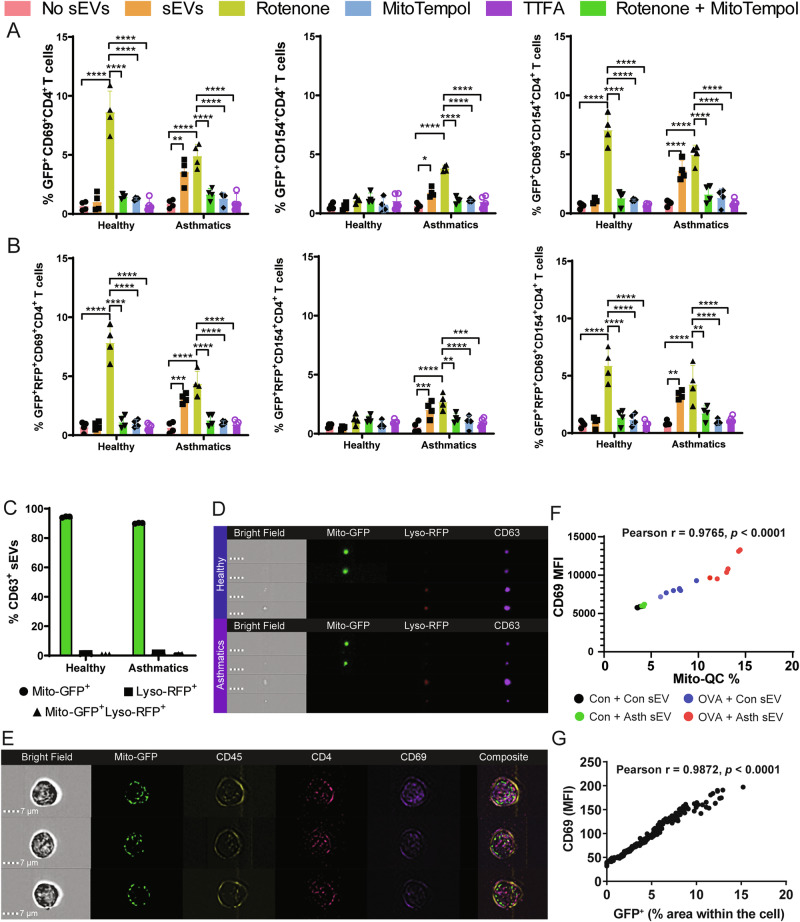
Reduction of mitochondrial ROS within sEVs by MitoTEMPOL inhibits the activation of CD4^+^ T cells by MDRC-derived Mito-GFP^+^ sEVs from asthmatics. Enhanced expression of early activation markers in CD4^+^ T cells in vivo in mice that received intranasal transfer of MDRC-derived sEVs isolated from allergen-sensitized and challenged Mito-QC mice. **A**, **B** MDRCs were transduced with CellLight Mito-GFP and CellLight Endo-RFP, and sEVs were purified from the supernatant 48 h later. sEVs derived from MDRCs transduced with Mito-GFP and Endo-RFP were pre-treated with 10 nM rotenone, MitoTEMPOL (1 μM), rotenone + MitoTEMPOL, TTFA (10 μM) overnight, or untreated prior to co-culture. sEVs were then washed and re-purified using the Invitrogen Total Exosome Isolation kit. MDRC-derived sEVs were co-cultured with autologous peripheral CD4^+^ T cells for 24 h in the presence of rhIL-2 (50 IU/ml) in a ratio of 1:10 T cells:sEVs. ImageStream flow cytometry was used to assess early activation (CD69) and antigen-specific activation (CD154). **A**, **B** Graphs illustrating the percentage of CD69^+^, CD154^+^, or CD69^+^CD154^+^ T cells that internalized **A** Mito-GFP^+^ MDRC sEVs, **B** both Mito-GFP^+^ and Endo-RFP^+^ MDRC sEVs. Two-way ANOVA, Tukey’s multiple comparisons test, (*n* = 4 for Healthy and Asthmatics, Mean of three replicates per sample are represented), ^*^p < 0.05, ^**^p < 0.01, ^***^p < 0.001, ^****^p < 0.0001. **C**, **D** MDRCs were transduced with CellLight Mito-GFP and CellLight Lyso-RFP, and sEVs were purified from the supernatant 48 h later. Purified sEVs were characterized using ImageStream flow cytometry to assess if the Mito-GFP and Lyso-RFP co-localized. **C** Quantitation of CD63^+^ MDRC sEVs that are Mito-GFP^+^Lyso-RFP^−^, Mito-GFP^−^Lyso-RFP^+^, or Mito-GFP^+^Lyso-RFP^+^ (*n* = 3 each for Healthy and Asthmatics, Mean of two replicates per sample are represented). **D** Representative image strips from ImageStream analysis illustrating Mito-GFP^+^Lyso-RFP^−^ MDRC sEVs, and Mito-GFP^−^Lyso-RFP^+^ MDRC sEVs. **E** sEVs were isolated from conditioned media of proinflammatory Ly6G^+^ lung MDRCs sorted from control, or OVA sensitized and challenged donor Mito-QC mice using the Total Exosome Isolation kit (*n* = 7 mice/group). Isolated sEVs were then i.n. delivered (1 × 10^8^ particles/mouse in 30 *μ*l PBS) to recipient sensitized and challenged mice as before. Lung tissue was harvested and digested two days after sEV delivery. Image strips showing co-localization of GFP^+^ signal in CD45^+^CD4^+^CD69^+^ T cells. **F** Pearson correlation analysis between Mito-QC (%) and CD69 expression (MFI) on CD4^+^ T cells from (**E**). The data were analyzed by the IDEAS 6.2 software. The black dots represented Con + Con sEV group (*n* = 5 mice), the green dots represented Con + Asth sEV (*n* = 5 mice), the blue dots represented OVA + Con sEV group (*n* = 7 mice), and the red dots represented OVA + Asth sEV group (*n* = 7 mice). **G** Pearson correlation analysis between Mito-QC (%) and CD69 expression (MFI) on CD4^+^ T cells. The percent area of GFP^+^ signal or mean fluorescence intensity (MFI) of CD69^+^ within each cell (*n* = 430). Individual data points are presented with each bar representing the mean ± SD. Source data are provided as a Source Data file.

Next, we asked whether ROS levels within sEVs are dependent on complex I, and if the T cell activation is dependent on sEV ROS. We used MitoTEMPOL, a superoxide dismutase mimetic, to scavenge ROS within the MDRC-derived sEVs following rotenone treatment (Fig. 5A). To test if blocking complex II resulted in a similar effect to rotenone or antimycin A, we used thenoyltrifluoroacetone (TTFA), a complex II inhibitor. When MDRC-derived sEVs were treated with rotenone and MitoTEMPOL, the rotenone-induced activation of CD4^+^ T cells was inhibited (Fig. 5A, B) with a reduction in % GFP^+^CD69^+^CD4^+^, GFP^+^CD154^+^CD4^+^, and GFP^+^CD69^+^CD154^+^CD4^+^ T cells, specifically in CD4^+^ T cells that have internalized Mito-GFP^+^ or both Mito-GFP^+^ and Endo-RFP^+^ (Fig. 5A, B) but not just the Endo-RFP^+^ MDRC-derived sEVs (not shown). Furthermore, treatment of MDRC-derived sEVs with TTFA did not induce CD4^+^ T cell activation, suggesting that complex II is not involved.

Since epithelial cells are the first responders to environmental cues that induce oxidative stress and influence sEV production, we investigated if their sEVs modulate CD4^+^ T cell activation. Although CD45^neg^ cells produced Mito-GFP^+^ sEVs and T cells internalized these sEVs, T cell activation was not observed (Fig. S9A–D). Therefore, sEVs derived from MDRCs or other APCs, are important in facilitating sEV-mediated T cell activation.

### NF-κB signaling is enhanced in CD4^+^ T cell co-cultures with Human BALF sEVs treated with rotenone, a complex I inhibitor

Since CD4^+^ T cell activation required ROS and the complex I inhibitor rotenone promoted this T cell activation, we evaluated ROS-driven signaling pathways underlying T cell activation and polarization. NF-κB family proteins are transcription factors that are important in inflammation and ROS impacts NF-κB signaling; NF-κB targets also may regulate levels of ROS^52^. Canonical NF-κB members, RelA(p65) and c-Rel, have a central role in mediating TCR signaling and T-cell activation^53^. NF-κB also regulates T-cell differentiation and effector function through proinflammatory cytokines^54,55^, with RelA recently being identified, as a requirement for Th17 polarization^56,57^. We evaluated sEV-mediated effects on NF-kB signaling in the presence or absence of rotenone. In CD4^+^ human T cells that internalized MitoTG^+^ sEVs (Fig. S10A–C), the proportions of NF-kB p65(pS529)^+^ cells increased in the presence of rotenone more in asthmatics compared to healthy subjects (Fig. S10D, E). RelA expression was also increased in T cells of asthmatics in the presence of rotenone (Fig. S10F). These data suggest that reverse electron transport (RET) does not mediate these effects, since rotenone inhibits RET. Alternative mechanisms are not clear at this point, but could include the impact due to specific sites of mitochondrial ROS production on RelA activation or accumulation of TCA substrates capable of mediating cell signaling^58^.

### Mito-GFP^+^ MDRC-derived sEVs internalize at the site of cytoskeletal polarization and mitochondrial trafficking in CD4^+^ T cells

**Fig. 6:**
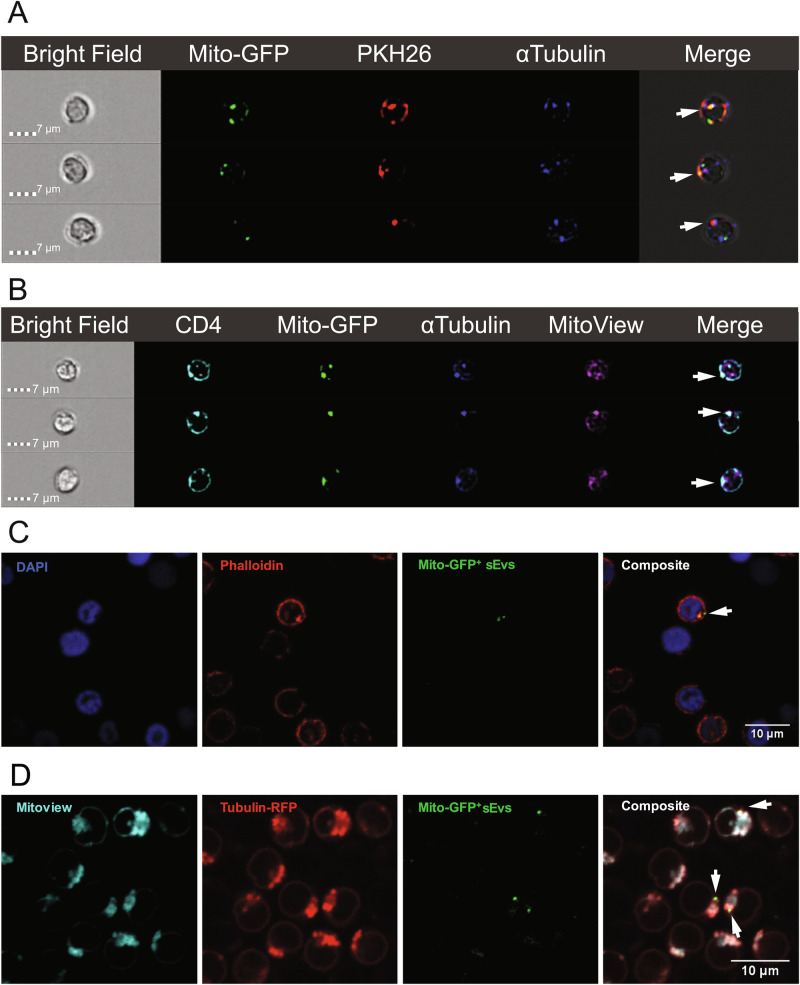
Internalization of Mito-GFP^+^ MDRC sEVs occurs at the site of tubulin, actin, and mitochondrial polarization in T cells. **A** Representative images from ImageStream flow cytometry analysis illustrating polarization of α-tubulin at the site of internalization of Mito-GFP^+^ sEVs (white arrow). MDRCs were transduced with CellLight Mito-GFP, and sEVs were purified from the supernatants 48 h later. Mito-GFP^+^ MDRC sEVs were membrane labeled with PKH26 red dye. T cells were transduced with CellLight Tubulin-RFP 24 h prior to co-culture with sEVs. MDRC sEV co-culture with T cells (1:10 T cell:sEV) was carried out for 24 h and analyzed on the ImageStream flow cytometer. **B** Representative images from ImageStream flow cytometry analysis illustrating polarization of α-tubulin and host mitochondria to the site of MDRC sEV internalization (white arrow). T cells were transduced with CellLight Tubulin-RFP and labeled with MitoView Deep Red dye 24 h prior to co-culture with MDRC sEVs. sEVs derived from MDRCs transduced with Mito-GFP were co-cultured with T cells (1:10 T cell:sEV) for 24-h and analyzed on the ImageStream flow cytometer. **C** Confocal image illustrating internalization of MDRC sEVs inside the cytoplasm of the CD4^+^ T cells (white arrow). sEVs derived from MDRCs transduced with Mito-GFP were co-cultured with T cells (1:10 T cell:sEV) for 24-h in the ibidi µ-dish. Cells were fixed with 2% PFA and permeabilized with 0.5% triton-X in PBS. The fixed T cells were labeled with Phalloidine-Rhodamine for actin, and DAPI for the nucleus. Cells were imaged on the Nikon A1 confocal. Experiments were performed in *n* = 6-8/study group. **D** Confocal image illustrating polarization of α-tubulin and host mitochondria to the site of MDRC sEV internalization (white arrow). T cells were transduced with CellLight Tubulin-RFP and labeled with MitoView Deep Red dye 24 h prior to co-culture with sEVs. sEVs derived from MDRCs transduced with Mito-GFP were co-cultured with T cells (1:10 T cell:sEV) for 24 h in the ibidi µ-dish and imaged on the Nikon A1 confocal.

We determined if the internalization of Mito-GFP^+^ sEVs occurs at the CD4^+^ T cell immune synapse, where cytoskeletal structures polarize with the mitochondria at the site of TCR engagement. When PKH-26 labeled MDRC-derived Mito-GFP^+^ sEVs were co-cultured with autologous peripheral T cells, the Mito-GFP^+^ sEVs co-localized with both α-tubulin, marking the CD4^+^ T cell cytoskeleton, and the polarized CD4^+^ T cell mitochondria (Fig. 6A). In CD4^+^ T cells labeled with both α-tubulin and Mitoview (labels mitochondria), the Mito-GFP^+^ sEVs co-localized with mitochondria and cytoskeleton (Fig. 6B). Confocal analysis of phalloidin and 4′,6-diamidino-2-phenylindole (DAPI) labeled CD4^+^ T cells co-cultured with Mito-GFP^+^ sEVs, revealed both intracellular and membrane co-localization of the sEVs with actin (Figs. 6C and S11). A z-stack slice of the confocal images indicated co-localization (yellow) of the Mito-GFP^+^ sEVs with cytoplasmic actin in the T cells (Fig. S11 and Movie Supplementary Video 1). Live confocal imaging of Tubulin-RFP transduced, and Mitoview-labeled T cells, co-cultured with Mito-GFP^+^ sEVs, also showed co-localization of Mito-GFP with the polarized cytoskeleton and mitochondrial network of the recipient CD4^+^ T cells (Fig. 6D and Movie Supplementary Video 2). These data suggest that sEVs are internalized at the site of the immune synapse, which may aid in the organization of the synapse through cytoskeletal and mitochondrial reorganization.

### Mitochondrial dynamics in sEV mitochondria-mediated T cell responses

We explored whether mitochondrial dynamics involving mitophagy contribute to packaging mitochondria within sEVs. Mitophagy is shared with the endosomal sorting complexes required for transport (ESCRT) pathway^59^. We transduced MDRCs with CellLight Mito-GFP and Lyso-RFP, a baculovirus construct that encodes lysosomal localization of RFP protein. The Mito-GFP^+^ and Lyso-RFP^+^ signals were mutually exclusive in the purified sEVs, indicating that Mito-GFP^+^ sEVs were not generated by mitophagy (Fig. 5C, D).

To ascertain that it is not mitophagy-mediated shuttling of mitochondria in sEVs that elicits pathogenic Th responses in vivo, we utilized Mito-QC mice. Quenched GFP and stable mCherry signal marks mitophagy in Mito-QC mice^60^. Both purified BALF EVs (Fig. S12A–D) and MDRC-derived EVs from lungs of asthmatic or OVA sensitized Mito-QC mice (Fig. S12E–H) were predominantly 65-150 nm with a median size consistent with sEVs. We utilized nanoimaging studies to further confirm the size (Fig. S13A) and EV marker distribution of MDRC-derived sEVs (Fig. S13B) and observed subpopulations of sEVs co-expressing sEV markers, including CD81, CD63, and CD9. The sEVs were predominantly, either CD63^+^ or CD63^+^CD9^+^ in both study groups. High-resolution imaging of MDRC-derived sEVs confirmed the co-expression of sEV markers in both groups (Fig. S13C). Cryo-electron microscopy of MDRC-derived sEVs from lungs of both asthmatic and OVA sensitized mice showed lipid bilayered vesicles within the sEV size range with electron-dense inclusions; multi-vesicular bodies were present in the asthma group (Fig. S13D, E). We performed intranasal transfer of these lung MDRC-derived sEVs from Mito-QC asthmatic or OVA sensitized donor mice to OVA sensitized and OVA challenged recipient mice. ImageStream analyses showed that lung-derived CD45^+^CD4^+^CD69^+^ activated T cells contained a stable mitochondrial network with unquenched GFP signal in transfer recipient asthmatic mice compared to controls (Fig. 5E). Correlation analyses showed that CD69 MFI correlated with % GFP^+^ CD4^+^ T cells as well as % GFP area within T cells of asthmatic recipient mice (Fig. 5F, G).

**Fig. 7:**
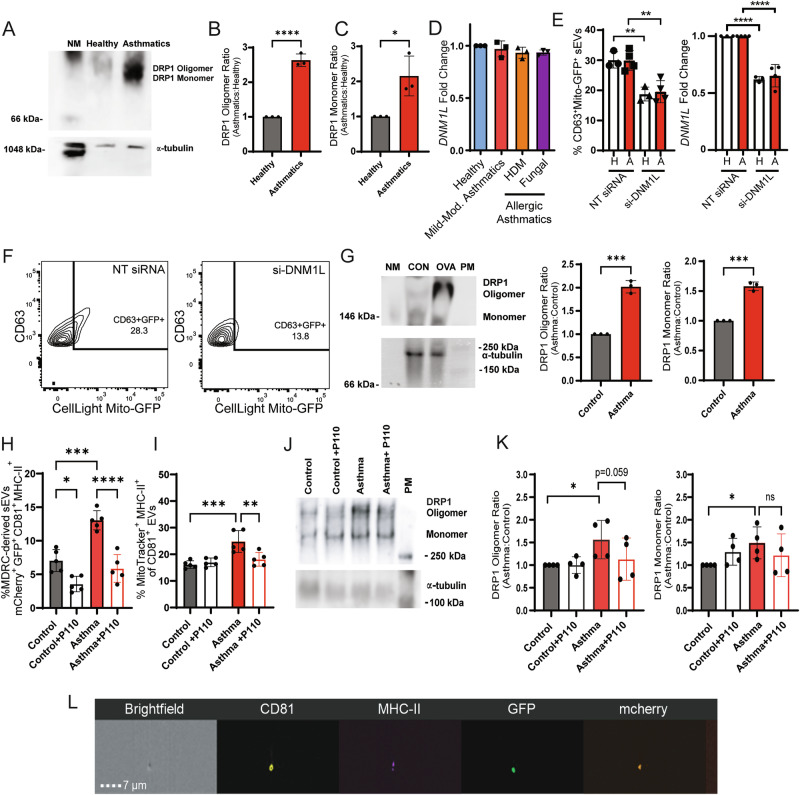
DRP1 oligomerization is enhanced in airway MDRCs from mice and humans with asthma. Packaging of stable mitochondrial network in MDRC-derived sEVs is enhanced in asthma and is dependent on DRP1. **A** Protein level and oligomerization of DRP1 were assessed in HLA-DR^+^ MDRCs sorted from the BAL of healthy and asthmatics by Native-PAGE with β-actin as a control. **B** Quantitation of the DRP1 oligomer ratio in samples from asthmatics and healthy. **C** Quantitation of DRP1 monomer ratio in samples from asthmatics and healthy. **A**–**C** Mann–Whitney test, samples were pooled from *n* = 3/study group, densitometry analysis shown from *n* = 3 western blots, ^*^p < 0.05, ^**^p < 0.01. **D** Quantitation of real-time PCR analysis of *DNM1L* gene expression in purified HLA-enriched peripheral myeloid cells from healthy, mild-to-moderate asthmatics, and allergic asthmatics (house dust mite or fungal). Human airway MDRCs were transfected with either non-target (NT) control siRNA, or siRNA that targets DNM1L using lipofectamine 3000 and cultured for 48 h in 10% human serum media that has been depleted of EVs, including sEVs. Culture supernatant was isolated for sEV isolation, and cells for gene expression analysis of *DNM1L* (*ACTB* as reference, *n* = 3). **E** Left: quantitation of percent CD63^+^Mito-GFP^+^MDRC-derived sEVs (*n* = 3 Healthy, *n* = 4 Asthma). Right: fold-change of *DNM1L* (DRP1) expression in transfected MDRCs to confirm siRNA efficacy (*n* = 3 Healthy, *n* = 4 Asthma). One wayANOVA, ^**^p = 0.006 for comparisons between healthy samples, ^**^p = 0.004 for comparison between asthma samples, ^****^p < 0.0001. **F** Representative flow plots illustrating the reduction of CD63^+^Mito-GFP^+^MDRC-derived sEVs after transfection with NT or siRNA targeting *DNM1L*. **G** Protein level and oligomerization of DRP1 were assessed in Ly6G^+^MDRCs sorted from the lungs of control or OVA-challenged mice by Native-PAGE. The relative expression of DRP1 oligomer or monomer was normalized with α-tubulin (Right, densitometry analysis shown from *n* = 3 western blots). **H** Proinflammatory Ly6G^+^MDRCs were sorted from the lungs of control or OVA-challenged Mito-QC mice. DRP1 oligomerization and binding to fission complex proteins were inhibited by treatment with P110 peptide (1 µM) or Vehicle control (Veh) for 48 h. sEVs were isolated from conditioned media of these cultures using the Total Exosome Isolation kit (*n* = 5 mice/group). Quantitation of the percentage of MDRC-derived mCherry^+^GFP^+^ MHC-II^+^CD81^+^ sEVs produced by treated cells within the CD81^+^ gate was characterized by ImageStream analyses. One-way ANOVA analysis was performed. ^*^p = 0.0161, ^***^p = 0.0001, ^****^p < 0.0001. **I** Proinflammatory Ly6G^+^MDRCs sorted from the lungs of control or OVA-challenged C57BL/6 mice were treated with P110 peptide (1 µM) or Vehicle control for 48 h as in (**H**). sEVs were isolated from conditioned media of these cultures using the Total Exosome Isolation kit (*n* = 5 mice/group). These sEVs were then stained with MitoTracker Green as described before, and sEVs were purified after washing. Quantitation of the percentage of MDRC-derived MitoTracker^+^MHC-II^+^CD81^+^ sEVs produced by treated cells within the CD81 gate was characterized by ImageStream analyses and shown. One-way ANOVA analysis was performed. ^**^p = 0.0054, ^***^p = 0.0005. **J** Native PAGE Western Blot analyses of Ly6G^+^ MDRC cell lysate post-treatment from (**H**) and probed with DRP1 antibody. Expression of DRP1 oligomer and monomer was detected. Alpha tubulin expression was used as a loading control. 5 mice per group (Control and Asthma), and 3 replicate experiments were performed for sorting MDRCs. **K** Quantitation of the DRP1 oligomer ratio in samples from Control and Asthma groups treated with P110 or Vehicle control (left). Quantitation of DRP1 monomer ratio in samples from Control and Asthma groups treated with P110 or Vehicle control (right). Mixed Effect ANOVA with Sidak’s multiple comparison test (Densitometric analyses of *n* = 4 western blots) ^*^p < 0.05. **L** Representative image strip from ImageStream analysis illustrating mCherry^+^GFP^+^MHC-II^+^CD81^+^ sEVs. Individual data points are presented with each bar representing the mean ± SD. Source data are provided as a Source Data file.

Mitochondria may be packaged in sEVs by dynamin-dependent mitochondrial fission, which is regulated by DRP1. As dynamin proteins, such as DRP1, oligomerize to function as facilitators of mitochondrial fission, we compared DRP1 oligomerization in HLA-DR^+^ MDRCs from healthy human individuals and asthmatics. Reduced levels of DRP1 monomers and oligomers were noted in HLA-DR^+^ MDRCs from healthy individuals compared to asthmatics (Fig. 7A–C). Gene expression of DNM1L was not significantly different comparing healthy, mild to moderate, and allergic asthmatics (Fig. 7D). We transduced MDRCs with CellLight Mito-GFP, transfected them with either non-target control siRNA or siRNA targeting *DNM1L*, the gene encoding DRP1, and confirmed knockdown of *DNM1L* by gene expression analysis (Fig. 7E, right panel). The percent of Mito-GFP^+^ sEVs was significantly reduced following *DNM1L* knockdown (Fig. 7E, left panel, F). This enhanced mitochondrial fission in MDRCs from asthmatics may alter DRP1 oligomerization (Fig. 7A–C), and promote the packaging of mitochondria in sEVs, and elicit sEV-mediated activation in CD4^+^ T cells. The oligomerization of DRP1 was also enhanced in pro-inflammatory MDRCs sorted from OVA-challenged Mito-QC mice compared to sensitized controls (Fig. 7G). We then treated MDRCs sorted from OVA-challenged Mito-QC mice and sensitized controls with P110 peptide, an inhibitor of mitochondrial fission and DRP1 activity. The percentage of mCherry^+^GFP^+^CD81^+^MHCII^+^ sEVs derived from MDRCs was increased in the asthma group compared to sensitized controls (Fig. 7H); these percentages were significantly reduced by treatment with P110 compared to vehicle-treated controls. Similar observations were made in MDRCs isolated from control and asthma groups of C57BL/6 mice following treatment with P110; the percentage of MitoTracker^+^CD81^+^MHCII^+^ sEVs was reduced in the asthma group following treatment compared to controls (Fig. 7I). The increased DRP1 oligomerization in MDRCs from the asthma group was reduced after P110 treatment (*p* = 0.059) (Fig. 7J, K). Image Stream analyses confirmed the characterization of mCherry^+^GFP^+^CD81^+^MHCII^+^ sEVs isolated from proinflammatory Ly6G^+^ lung MDRCs (Fig. 7L). These data suggest that increased DRP-1-dependent mitochondrial dynamics in MDRCs contribute to mitochondrial packaging in sEVs.

### Mito-GFP^+^ MDRC-derived sEVs may elicit both innate and adaptive immune responses

We determined if T cell activation was HLA-specific or due to allogeneic responses. We co-cultured HLA-DR^+^ MDRC-derived sEVs from asthmatics with CD4^+^ T cells from healthy subjects, and vice versa. Modest CD69 activation with unchanged CD154 was seen in CD4^+^ T cells that had internalized Mito-GFP^+^ or both Mito-GFP^+^ and Endo-RFP^+^ MDRC-derived sEVs (Fig. S14A–C) compared to autologous co-cultures (Figs. 3, 4, and S5–S8). These data demonstrate that MDRC-derived sEVs elicit HLA-specific activation. We then investigated the direct effects of sEV-mitochondria on innate immune cells^61,62^. MDRC-derived sEVs from asthmatics and healthy subjects were co-cultured with a human myeloid cell line, THP-1, for 24 h. Pro-inflammatory cytokines increased in THP-1 cells treated with sEVs of asthmatics (Fig. S14D–I), while healthy sEVs reduced MCP-1 levels (Fig. S14E). These results indicate that sEVs from asthmatic individuals can promote both innate and adaptive immune responses.

### Mito-GFP^+^ MDRC-derived sEVs drive Th2, Th17, and Th2/17 polarization

**Fig. 8:**
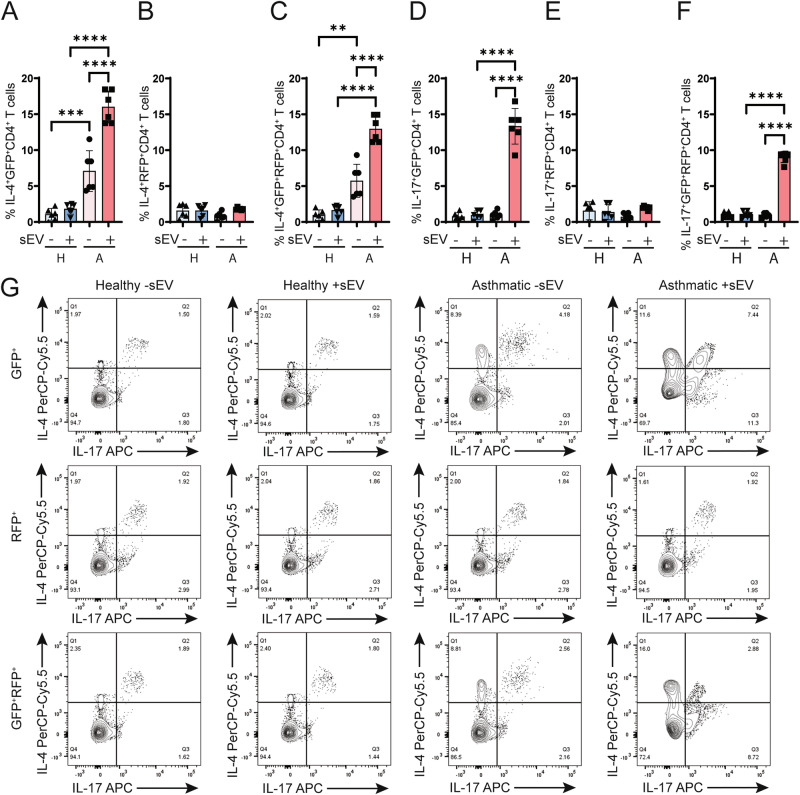
T cells that internalize MDRC-derived Mito-GFP^+^ sEVs from asthmatics polarize to Th17 and Th2 subsets. **A**–**F** Airway MDRCs were transduced with CellLight Mito-GFP and CellLight Endo-RFP, and sEVs were purified from the supernatant 48 h later. Purified MDRC sEVs were co-cultured with autologous peripheral CD4^+^ T cells for 7 days in the presence of rhIL-2 (50 IU/ml) in a ratio of 1:10 T cells:sEV. T helper subsets were assessed by flow cytometry 7 days later by intracellular staining for IL-4 (Th2) and IL-17 (Th17) cytokines. Cells were gated for CD4^+^GFP^+^IFNγ^−^ cells, and then identified as either IL-4^+^IL-17^−^ or IL-4^−^IL-17^+^. Similar gating strategies were used for CD4^+^RFP^+^cells and CD4^+^GFP^+^RFP^+^cells as shown in Supplementary Fig. S15. Experiments were performed in *n* = 6 for healthy and *n* = 6 for asthmatics. Mixed effect ANOVA with Sidak’s multiple comparison tests were done, ^**^p < 0.01, ^***^p < 0.001, ^****^p < 0.0001. **G** Representative flow cytometry plots of cells gated as above, showing % Th2 and % Th17 cells shown as % IL-4^+^ and % IL-17^+^CD4^+^ T cells from the co-cultures with sEVs. Flow plots are shown for these Th cell populations that are GFP^+^, RFP^+^, or GFP^+^RFP^+^ in experimental groups of Healthy −sEV, Healthy + sEV, Asthmatic −sEV and Asthmatic +sEV. Individual data points are presented with each bar representing the mean ± SD. Source data are provided as a Source Data file.

As BALF sEVs drive Th2 and Th17 polarization (Figs. 1E–I and S3C), we assessed whether airway MDRC-derived Mito-GFP^+^ sEVs directly promote Th polarization. Flow cytometry analyses showed IL-4-producing Th2 and IL-17-producing Th17 responses only in T cells that have internalized Mito-GFP^+^ and not the Endo-RFP^+^ sEVs alone (Figs. 8A–G and S15). Additionally, healthy MDRC-derived Mito-GFP^+^ sEVs induced IFN-γ producing Th1 responses (Figs. S16A–C, G). Th2/Th17 hybrid cells that produce both IL-4 and IL-17 are also potent inducers of airway inflammation in asthmatics^11,63^. The MDRC-derived sEVs from asthmatics induced Th2/17 hybrid responses only in T cells that internalized Mito-GFP^+^ and not Endo-RFP^+^ sEVs (Figs. S16D, H and S13F).

We then investigated whether rotenone-mediated sEV-mediated NF-κB signaling enhances Th responses in co-cultures. Increase in percent of CD4^+^ T cells with activated NF-κB p65 (Fig. S17A, B) and enhancement of IL-17^+^ Th17 and IL-4^+^ Th2 cells (Fig. S17A–E) within these activated cells were noted in rotenone-treated sEV-T cell co-cultures from asthmatics compared to vehicle-treated sEVs and T cell co-cultures of healthy subjects; sEV-mitochondria-derived ROS-mediated NF-κB signaling may drive Th responses in asthmatics.

### In vivo transfer of lung sEVs with a stable mitochondrial network enhanced airway inflammation, lung pathology, and pathogenic Th responses in allergic recipients

**Fig. 9:**
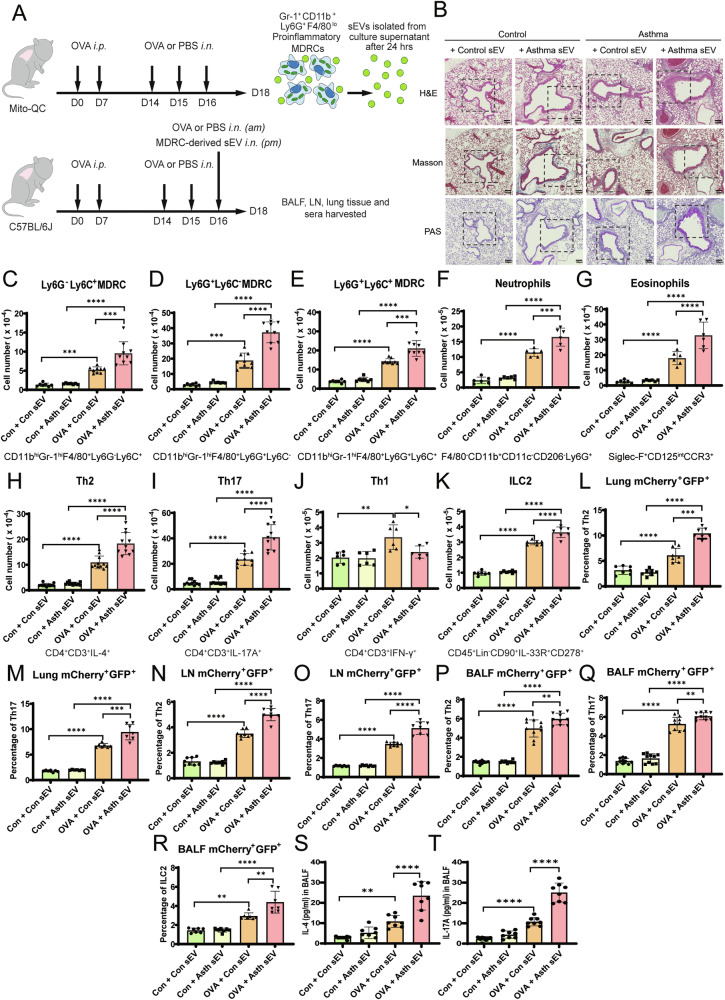
Intranasal transfer of pro-inflammatory lung MDRC-derived sEVs from sensitized and challenged donor Mito-QC mice with asthma exacerbates Th2 and Th17 responses and allergic airway inflammation and enhanced inflammatory cell infiltration, goblet cell hyperplasia, and mucus hypersecretion in sensitized and challenged recipients. Enhanced mCherry^+^GFP^+^ Th2 and Th17 cell infiltration in the lung tissue, draining LNs, and BALF were observed in sensitized and challenged recipients during allergic airway inflammation. **A** Schematic showing the experimental design for the murine asthma model and sEV transfer. Mice were sensitized by intraperitoneal injection on d0 and d7 with 50 *μ*g of alum-adsorbed OVA. On d14, d15, and d16, mice were challenged once i.n. with 15 *μ*g OVA in 30 *μ*l PBS or PBS alone. On d16, i.n. delivery of lung MDRC-derived sEVs (1 × 10^8^ particles/mouse in 30 μl PBS) from control or OVA-challenged Mito-QC mice was carried out as before. For isolation of MDRC-sEVs, 5 donor mice per group were used, and 3 replicate transfer experiments were performed. Lung infiltration of immune cells in lung tissue was determined by FACS analyses in the recipient mice. **B** Representative light-microscopy photomicrographs of lung sections from recipient experimental groups stained with hematoxylin and eosin for morphological analysis and assessment of inflammatory cell infiltration (top panel), Masson Trichrome to compare collagen accumulation (middle panel), and periodic acid-schiff (PAS) to assess changes in goblet cell hyperplasia and mucus production (bottom panel). Scale bars: 100 µm. (*n* = 3 mice per group). **C**–**K** Cell numbers of Ly6G^-^Ly6C^+^ MDRC (**C**), Ly6G^+^Ly6C^-^ MDRC (**D**), Ly6G^+^Ly6C^+^ MDRC (**E**), neutrophils (**F**), eosinophils (**G**), Th2 (**H**), Th17 (**I**), Th1 (**J**), and ILC2 (**K**) in the lung tissue were determined by flow cytometry. *n* = 6-10 recipient mice/group. One-way ANOVA with Tukey’s multiple comparison testing, ^**^p < 0.01, ^***^p < 0.005, ^****^p < 0.001). For quantitation of mCherry^+^GFP^+^ Th2 and Th17 cell infiltration in the lung tissue, draining LNs, and BALF, 5 donor mice per group were used for isolation of MDRC-sEVs, and recipient mice were 3–4 mice/group. Infiltration of immune cells in the lung, draining LNs, and BALF was detected by FACS analyses at two days after sEV delivery. Frequencies of mCherry^+^GFP^+^ Th2 (**L**) and Th17 (**M**) in the lung tissue were determined by flow cytometry. Frequencies of mCherry^+^GFP^+^ Th2 (**N**) and Th17 (**O**) in the draining LNs were characterized by flow cytometry. Frequencies of mCherry^+^GFP^+^ Th2 (**P**), Th17 (**Q**), and ILC2 (**R**) in BALF were determined by flow cytometry. *n* = 7–10 recipient mice/group. **S** Levels of IL-4 and IL-17A (**T**) in BALF determined by ELISA (*n* = 8 mice/group, mean of two replicate measurements shown for each). Statistical significance was evaluated using one-way ANOVA with Tukey’s multiple comparison testing. ^**^p < 0.01, ^***^p < 0.005, ^****^p < 0.0001). Individual data points are presented with each bar representing the mean ± SD. Source data are provided as a Source Data file.

We observed that the intranasal transfer of these lung MDRC-derived sEVs from Mito-QC asthmatic or OVA sensitized donor mice also enhanced overall inflammatory cell infiltration in the lungs, airways, and lung draining lymph nodes of asthmatic recipient mice compared to OVA sensitized controls (C57BL/6) (see Schematic in Figs. 9A and S18A, C). Following transfer of MDRC-derived sEVs, OVA-specific IgE in both BALF and serum was increased along with levels of Muc5AC in asthmatic recipient mice compared to OVA-sensitized controls (Fig. S18D–F). MDRC-derived sEVs isolated from OVA-sensitized controls elicited inflammatory responses of much lower magnitude when transferred to OVA-challenged mice and little to no response in OVA-sensitized mice (Fig. S18A–F). BALF differential also showed increased airway infiltration of eosinophils, neutrophils, macrophages, and lymphocytes in asthmatic recipient mice of MDRC-derived sEVs (Fig. S18G–J). Further, we investigated whether sEV transfer enhanced asthma-associated lung pathology in recipient mice. Histopathological evaluation of inflated lung tissues was performed in recipient mice following intranasal transfer of donor-derived MDRC sEVs from both control and asthma groups. As shown in Fig. 9B and S19, the overall inflammatory cell infiltration, goblet cell hyperplasia, mucus hypersecretion, and accumulation of collagen were all increased in lungs of allergen-challenged mice that received sEV transfers from mice with asthma compared to controls. The absolute numbers and frequencies of lung MDRCs, including Ly6C^+^, Ly6G^+^, and Ly6C^+^Ly6G^+^ subpopulations as well as neutrophils and eosinophils, also showed similar higher levels in asthmatic recipient mice following sEV transfer (Figs. 9C–G, S20A–S20E, and S21). Importantly, the absolute numbers and frequencies of Th2, Th17 cells (Figs. 9H–I and S20F–G) as well as Type 2 innate lymphoid cells (Figs. 9K and S20I) also increased in asthmatic recipient mice following sEV transfer compared to relevant controls; the absolute numbers and frequency of Th1 cells declined in mice with asthma (Figs. 9J and S20H). Thus, in vivo transfer of MDRC-derived sEVs with mitochondria enhanced asthma-associated lung pathology.

We then investigated whether intranasal transfer of these MDRC-derived sEVs from OVA-sensitized or challenged donor Mito-QC mice to sensitized or challenged recipients would enhance pathogenic Th responses and exacerbate allergic airway inflammation in vivo in recipients. As a stable mitochondrial network without mitophagy is marked by a binary signal of both mCherry and GFP in Mito-QC mice, we evaluated percentages and absolute numbers of mCherry^+^GFP^+^ Th2 and Th17 cells and found that both were increased in BALF, lung tissues, as well as lymph nodes (Figs. 9L–9Q and S22–S24) of asthmatic recipient mice compared to control recipients. Consistent with this, IL-4 and IL-17 levels were enhanced in the BALF of the recipient mice (Fig. 9S–T). These data confirmed that a stable mitochondrial network was transferred in vivo via sEVs to CD4^+^ T cell subsets to promote Th polarization. Additionally, increased proportion and absolute numbers of mCherry^+^GFP^+^ ILC2s were also noted in BALF of recipient mice with asthma compared to sensitized controls (Figs. 9R and S24G).

## Discussion

sEVs are novel intercellular messengers, both in homeostasis and disease. Our observations extend the role of sEVs as immune modulators in human asthma. We report that airway sEVs from asthmatics drive proliferation of Th cells, and their polarization to Th2 and Th17 phenotypes is supported by mitochondrial oxidant-dependent NF-κB signaling. Accompanying the changes in epigenetic programming, the airway HLA-DR^+^ MDRC-derived immunogenic sEVs containing mitochondria elicit antigen-specific activation that requires mitochondrial ROS signaling through the transfer of sEV mitochondria to recipient T cells; this transfer involves internalization of sEVs by membrane fusion that initiates TCR engagement/activation in CD4^+^ T cells, potentially at the site of the immune synapse. We demonstrate that in vivo transfer of a stable mitochondrial network carrying sEVs can enhance in vivo pathogenic Th polarization, Th cytokine production, and enhanced pathology in the lungs of allergic recipients.

CD4^+^ T cells use clathrin-dependent mechanisms to recycle cell surface receptors^49^; however, transfer of the PKH26 signal from labeled sEVs to CD4^+^ T cell membranes, internalization of the mitochondrial GFP signal, and the preservation of sEV internalization by CD4^+^ T cells in the presence of inhibitors of clathrin or dynamin, or LFA-1 antibody, suggest that membrane fusion is the primary mechanism of internalization of mitochondria-containing sEVs. Although CD4^+^ T cell activation correlated with Mito-GFP^+^ sEV internalization, blockade of class II or LFA-1 on sEVs failed to inhibit their internalization, but attenuated activation of recipient CD4^+^ T cells. LFA-1 helps bind T cells to APCs via ICAM-1 and ICAM-2, which aids in antigen presentation^64^, and CD8^+^ T cells utilize LFA-1 to internalize sEVs^65^. Synergistic signaling between class II and LFA-1 may facilitate MDRC-derived sEV-mediated antigen-specific T cell activation without APCs.

Our studies indicate that mitochondria within sEVs are bioenergetically functional and required for sEV-mediated TCR activation. Inhibition of mitochondrial complex II, complex III, and complex V abrogated Mito-GFP^+^ sEV-mediated T cell responses. Interestingly, pre-treatment of MDRC-derived sEVs with complex I inhibitor, rotenone, aberrantly activated healthy T cells (CD69^+^) and promoted antigen-specific activation in asthmatics. Since these mitochondrial inhibitors block oxidative phosphorylation, this aberrant activation could be ATP-dependent. However, mitochondria-targeted antioxidant, mitoTEMPOL, scavenged the ROS within MDRC-derived sEVs after rotenone treatment, implicating ROS in the sEV-dependent activation of healthy T cells. Rotenone-induced activation was accompanied by NF-κB signaling as well as RELA upregulation. Additionally, Th differentiation occurred in these cells with induced NF-kB signaling; this is consistent with the implication of NF-κB signaling in T cell activation and Th differentiation. Our data, thus, suggests that it is not RET-mediated and may be due to rotenone-mediated ROS production altering normal T cell function^51,58,66,67^. Although low rotenone doses were used on sEVs, and the high affinity of this inhibitor prevents it from leaving sEVs, these concentrations may cause off-target effects; additional downstream mechanisms for rotenone-dependent effects may need to be further clarified^68^. Despite these caveats, our studies implicate mitochondrial ROS as an essential mediator of T cell activation. T cells produce ROS via complex I after TCR engagement^69^, and complex I is important for T helper differentiation/polarization^70^. This is consistent with activation of Th transcriptional program in T cells by mitochondrial signaling via MDRC-sEVs. sEV-mediated activation of Th1 transcriptional regulators may be suppressed by robust activation of Th2 and Th17 programs in T cells from asthmatics, and this threshold is different in healthy T cells; this difference may account for Th1 polarization seen in healthy T cells. Taken together, our studies suggest that CD4^+^ T cell-activation by sEVs in the context of asthma or other chronic inflammatory diseases may depend on both TCR-MHC engagement and transfer of functional mitochondria capable of generating ROS.

We interrogated mechanisms underlying mitochondrial packaging within airway myeloid cell-derived sEVs. Mesenchymal stem cells package mitochondria in extracellular vesicles to outsource mitophagy^36^. In our studies, the absence of co-localization of the packaged Mito-GFP^+^ signal with Lyso-RFP^+^ associated with mitophagy, demonstrates a novel role for MDRC-derived sEVs in mitochondrial retrograde signaling. Although TLR-mediated activation of immune cells by mitophagy is possible^71^, our results suggest that antigen presentation and mitochondrial ROS signaling are the primary mechanisms of immune activation.

Although transfer of mitochondria by sEVs has been reported^72^, mechanisms of packaging mitochondria within airway immune cell-derived sEVs are largely unknown. We provide insights into DRP-1’s role and potentially mitochondrial fission in the generation of sEVs that transport functional mitochondria. Our observations that intranasal transfer of Mito-QC mice-derived sEVs with a binary signal of mCherry and GFP results in exacerbation of allergic airway inflammation and enhancement of pathogenic Th polarization and lung pathology in allergic models, provide in vivo evidence for the significance of sEV mitochondria in asthma. While current approaches and available sEV inhibitors do not enable efficient targeting of specific sEVs or sEV-mediated signals, future studies using genetic strategies and sEV transfer approaches are warranted to investigate if inhibition of sEVs/sEV-specific signals attenuates asthma responses, and thereby could provide the basis for future EV-targeting therapies of asthma.

DRP-1-dependent mitochondrial fission requires its oligomerization^73^; here, DRP-1 oligomerization was increased in MDRCs from asthmatics and in mice with asthma. Importantly, knockdown of DRP-1 or inhibition of DRP-1 activity reduced the packaging of mitochondria within sEVs. Mitochondrial fragmentation alters immune function in cancer; this limited the function of NK cells^74^. In our studies, DRP-1 activity and consequent mitochondrial fission account for the generation of sEVs with mitochondria that facilitate sEV-mediated immune activation, and a role for mitochondrial fission in immune regulation. The extent of sEV signaling is different between healthy controls and asthmatics, including ROS and NF-κB-mediated signaling. Genetic differences are, so far, not known to contribute to differences in proinflammatory MDRCs reported in asthmatics. Epigenetic modifications from lung damage/oxidative stress may alter MDRC-derived sEVs in asthmatics and/or the differential presence/presentation of antigens by sEVs and/or may account for the increased mitochondrial fission noted in MDRCs from asthmatics; this differential response may not be restricted to asthma and may be important in COPD or sarcoidosis.

Additionally, we demonstrate that Mito-GFP^+^ sEV-mediated signaling and internalization occur in close proximity to the polarized cytoskeleton of activated T cells, often associated with synapse formation in activated T cells, which provides an antigen-specific dimension to this cellular crosstalk. Our observation that the polarized cytoskeleton and the mitochondria of the activated CD4^+^ T cell co-localize with sEV mitochondria from donor MDRCs suggests that upon TCR engagement by the MHC-II on sEVs, reorganization of the immune synapse may occur at the site of sEV internalization.

Here, we uncover an unexplored mechanism by which sEVs activate CD4^+^ T cells, and mitochondrial transfer may function as a second signal in CD4^+^ T cell activation. Traditionally, T cell activation/polarization requires antigen presentation by APCs, co-stimulation, and an optimal cytokine milieu^75–77^. Our studies support an acellular form of T cell activation by sEV mitochondria and a new mechanism of MDRC-T cell crosstalk in vitro and in vivo at the intersection of bioenergetics and cellular function. We propose that these intricate immunological mechanisms facilitated by sEVs participate in both normal physiological responses and may be dysregulated in pathological processes such as human asthma.

## Method

### Human subjects

This study was approved by the University of Alabama at Birmingham Institutional Review Board (Protocol IRB-151209005), and written informed consent was obtained from all participants. All procedures performed in this study were performed in accordance with relevant guidelines and regulations. Asthmatic and healthy control subjects were screened by the clinical coordinator based on the inclusion and exclusion criteria of the approved IRB protocol. These included IgE titer, blood eosinophil frequencies, past medical history, and FEV_1_ (Table 1). Study subjects were then enrolled through the University of Alabama at Birmingham Lung Health Center. Healthy controls did not have histories of asthma, pulmonary infections, or other known lung diseases. All asthmatic patients had a prior diagnosis of asthma and demonstrated a 12% or greater increase in FEV_1_ within 30 min of administering 400 µg of albuterol, as outlined in the GINA guidelines (Global Strategy for Asthma Management and Prevention; http://www.ginasthma.org/)^78^. Screened subjects with serum cotinine levels greater than 10 ng/ml (smokers) and those subjects who received treatment with inhaled or systemic corticosteroids within the six weeks prior to or during the study were excluded. Previous exposure to secondhand smoke was determined by LC-MRM mass spectrometry for serum cotinine levels between 0.05 and 10 ng/ml^79^. Healthy controls and asthmatics with serum cotinine levels below 0.05 ng/ml were considered non-SHS-exposed subjects. Bronchoscopies were carried out as previously described^21,80^. In brief, a total of 200 ml of saline solution was instilled, with an average return of 123.3 ± 39.09 ml among healthy individuals, and 92.14 ± 32.50 ml for asthmatics. Eighty (80 ml) of BAL fluid (BALF) was used for the isolation of sEVs. A total of 32 volunteer subjects were assessed in this study, of which 17 were in the healthy study arm, and 15 in the asthma study arm. The median IgE titer was significantly higher in the asthma study arm (Table 1).

### Primary cell culture

Primary cells isolated from BALF or peripheral blood were cultured in RPMI 1640 supplemented with 1% Penicillin-Streptomycin, 1% L-Glutamine, and 10% human AB serum, unless otherwise noted. sEV-depleted media were used (described below) when cells were cultured for sEV isolation or when sEVs were co-cultured with T cells. For primary T cell co-cultures, recombinant human IL-2 was supplemented at a final concentration of 50 IU/ml.

### Experimental allergic airway inflammation and intranasal adoptive transfer of MDRC-derived sEVs in murine models

Female C57BL/6J mice at 6 to 8 weeks of age were purchased from The Jackson Laboratory (Stock #: 000664). Mice were maintained in a conventional pathogen-free housing facility under standard conditions and handled in accordance with the Guidelines for Animal Experiments at the University of Alabama at Birmingham. Experimental and control animals were maintained in separate cages but co-housed in the same space within the animal facility. All protocols were approved by the University of Alabama at Birmingham Institutional Animal Care and Use Committee (Animal Protocol Numbers: 20273, 21974). Mito-QC mice (female mice in C57BL/6J background, 6–8 weeks of age) were obtained from the University of Dundee under a Material Transfer Agreement and were maintained and utilized according to the MTA guidelines. These mice express a functionally inert, tandem mCherry-GFP tag fused to the mitochondrial targeting sequence of the outer mitochondrial membrane (OMM) protein, FIS1 (comprising amino acids 101–152). Under steady-state conditions, the mitochondrial network fluoresces both red and green and can be monitored as mCherry^+^ GFP^+^. If there is mitophagy, mitochondria are delivered to lysosomes, where mCherry fluorescence remains stable, but GFP signal will be quenched. Mito-QC mice were sensitized by intraperitoneal injection (i.p.) on d0 and d7 with 50 *μ*g of alum-precipitated OVA (Grade VII, all reagents detailed in Supplementary Table 1), as previously described in refs. ^81,82^. On d14, d15, and d16, under anesthesia with Isoflurane, Mito-QC mice were challenged intranasally (i.n.) with 15 *μ*g OVA in 30 *μ*l PBS or PBS alone. On day 18, lung tissues were harvested from both the asthmatic and the control groups. Animals were euthanized with a two-step procedure compliant with NIH and IACUC guidelines under deep anesthesia, induced using ketamine hydrochloride in combination with xylazine. All procedures were performed under surgical plane anesthesia to ensure that pain and distress were appropriately relieved before euthanasia, consistent with Pain Category D of the IACUC guidelines. Gr-1^+^CD11b^+^Ly6G^+^F4/80^lo^ proinflammatory MDRCs were sorted and sEVs isolated from the culture supernatant after 24 h of culture. For intranasal transfer of isolated sEVs into mice with asthma, C57BL/6J mice were first sensitized by i.p. injection on d0 and d7 with 50 *μ*g of OVA. On d14 and d15, C57BL/6J mice were challenged i.n. with 15 *μ*g OVA in 30 *μ*l PBS or PBS alone. On d16, C57BL/6J mice were challenged i.n. with 15 *μ*g OVA in 30 *μ*l PBS or PBS alone in the morning, followed by i.n. delivery of lung MDRC-derived sEVs (1 × 10^8^ particles in 30 *μ*l PBS) from control or OVA-challenged Mito-QC mice in the afternoon. Two days after OVA challenge and sEV delivery, BALF and sera were harvested for OVA-IgE and Muc5AC ELISA. Lung tissues were digested with collagenase B, and immunophenotyping was carried out.

### Preparation of sEV-depleted media

sEV-depleted media was prepared by supplementing RPMI 1640 with 20% or 40% human AB serum or fetal bovine serum (FBS) and centrifuged on an ultracentrifuge overnight at 100,000xg at 4 °C. The supernatant was transferred to a new tube and sterile filtered through a 0.2 μm cellulose acetate filter. sEV-depleted media was then diluted 1:1 with RPMI 1640 to make a final concentration of 10% or 20% serum. sEV-depleted media was supplemented with 1% Penicillin-Streptomycin and 1% L-Glutamine, unless otherwise noted. Media was stored at 4 °C.

### Isolation of sEVs from BALF

sEVs were isolated using a previously described differential centrifugation method^42,83^. Briefly, 40 ml of BALF was centrifuged at 300×*g* for 10 min at 4˚C to remove airway cells. Then the supernatant was further centrifuged at 2000×*g* for 10 min at 4 °C to remove any dead cells and large cellular debris. The supernatant was spun again at 10,000×*g* for 30 min at 4 °C to remove any smaller cellular debris and finally filtered through a 0.2 μm cellulose acetate filter. The filtrate was then centrifuged at 100,000×*g* for 70 min at 4 °C, and the pellet was washed with fresh PBS to remove any contaminating proteins. Finally, the washed pellet was centrifuged at 100,000×*g* for 70 min at 4 °C, and the final pellet was resuspended in 100 µl of fresh PBS. The purified sEVs were stored at −80 °C.

### Isolation of sEVs from human airway HLA-DR^+^ MDRCs

Human airway HLA-DR^+^ MDRCs were sorted by FACS from the BALF of healthy and asthmatic volunteers as described in ref. ^21^. Cells isolated from the BALF were first blocked with RPMI 1640 containing 10% human AB serum for 30 min on ice and then stained with the following antibodies: CD11b APC-Cy7, CD169 BV510, HLA-DR APC, CD163 PE, CD33 PE-Cy7, CD14 PerCP-Cy5.5, and CD11c PE-Cy5. The pro-inflammatory HLA-DR^+^ MDRCs were identified as CD11b^+^HLA-DR^+^CD33^+^CD11c^+^CD163^+^CD14^lo^CD169^lo^ and sorted using a BD FACS Aria. Sorted cells were cultured in sEV-depleted human AB serum media for 48 h. Conditioned media were collected and centrifuged twice at 2000×*g* to remove any cell pellets and apoptotic bodies. sEVs were purified from the supernatant using the Total Exosome Isolation Reagent for Cell Culture Media kit, following the manufacturer’s protocol. Purified sEVs were stored in 50 µl of PBS at −80 °C.

### Quantitation of sEVs

The concentration and size distributions of purified airway sEVs were determined using a NanoSight NS300^84^. The instrument was calibrated using 100 nm polystyrene latex microspheres. The sEVs were diluted 1000-fold with PBS to make a final volume of 1 ml and loaded into a 1 ml syringe, which was placed on a syringe pump attached to the NanoSight. The diluted sEVs were injected into the NanoSight at a flow rate of 25 μl/s at room temperature. A total of 5 videos were acquired per sample under the following conditions and settings: temperature: 22.4–22.6 °C; viscosity 0.939–0.944 cP; camera level: 7; capture duration: 1 min/video; shutter speed: 11.12 ms; camera type: SCMOS; slider gain: 250; slider shutter: 250; minimum tracks completed: 2000–4000/video; frames processed: 1951/video; frames per second: 32.5 fps; blur: auto; detection threshold: 5. sEV particle measurements and size distributions were performed using Spectradyne’s nCS1 nanoparticle analyzer with Hardware Version 1^85^. Briefly, the microfluidic system was primed with 1% Tween20 in PBS. Diluents were first filtered through a 0.01 filter membrane, then C-400 or TS-400 cartridges (65-400 nm range) were used to determine the concentration spectral density (CSD) of particles. All experiments consisted of acquisitions of *n* > 3000 particles (Error < 3.2). After each measurement was completed, data were combined, and (i) peak filters and (ii) background subtraction were applied in the nCS1 Data viewer, as recommended by the manufacturer. To avoid false positive counts due to electrical noise, background subtraction was applied to all measurements, then sEV concentrations and particle size distribution (using Gaussian Fit) were determined.

### Cryo-electron microscopy

Murine MDRC-derived sEV samples (10^8^ particles) were applied to 300 mesh copper lacey carbon grids that had been glow-discharged at 30 mA for 25 s with a Pelco easiGlow glow discharger. For each grid, 3 ml samples were incubated on the grid for ~2 min, the grid was manually blotted, another 3 ml sample was added, and the grid was loaded into a Vitrobot Mark IV for the final blotting process. The Vitrobot was set to 12 °C, and 100% humidity, and the grids were blotted for 4 s before plunging into liquid ethane. The samples were imaged using the EPU software on a Glacios 2 electron microscope operated at 200 kV and equipped with a Falcon 4i direct detector^86^ at the UAB Cryo-EM Facility. Images were collected as TIFF files at a nominal magnification of 120,000× (1.19 Å/pix) with 3 mm defocus and either 40 or 60 e^−^/Å^2^ total dose. Using Fiji, a mean filter with a 3-pixel radius was applied to reduce high-resolution noise in the images, and scale bars were added.

### Nanoimaging and quantitation of sEVs

Nanoimaging and quantitation of sEVs were performed on the ONi Nanoimager in collaboration with Oxford Nanoimaging Company^87^. Briefly, purified human BALF sEVs were captured and stained using the Oxford Nanoimaging EV Profiler Kit 2 with the following modifications. Biotinylated antibodies against CD81, CD63, and CD9 were used to capture human EVs in the flow chambers. They were stained using the kit’s lipophilic dye, PanEV markers (biotinylated CD81, CD63, CD9), and an antibody against TOMM20 (1:200) and a secondary antibody (provided by Oni; donkey anti-mouse AZ647, 1:200). The secondary antibody was incubated for 30 min after washing the primary antibody away and prior to the post-fixation in the EV Profiler Kit 2 protocol. Imaging was completed using the AutoEV function. All analysis was completed in the CODI software. Mouse MDRC-derived sEVs were captured using a solution of poly-L-lysine and stained using the EV Profiler Kit 2 with primary conjugated antibodies against CD81, CD63, and CD9. These antibodies were raised in a mouse host and therefore may have a higher non-specific background signal. Their reactivity has not been extensively tested in murine samples. Imaging was completed using the AutoEV function on the ONi Nanoimager, and analysis was completed in the CODI software.

### Western blot analyses of sEVs

BALF-sEV samples isolated from 7 healthy human study subjects and 7 study subjects with asthma were pooled for a total concentration of 8 × 10^9^ sEVs each for both study groups. sEV samples were lysed in 10× RIPA buffer containing protease inhibitor cocktail. Samples were then sonicated at 30% amplitude on ice for 10 s and then incubated on ice for 15 min, mixed with loading buffer and heated at 95 °C for 8 min. Samples were then electrophoresed on a 15% acrylamide SDS-PAGE gel (Tris-Glycine). Samples were run into the stacking gel at 60 volts for 30 min, and then switched to 100 volts to run samples into the separating gel. The gels were then transferred onto methanol-pretreated 0.45 PVDF membranes. Gels were transferred at 100 mA in 20% methanol Tris-Glycine transfer buffer overnight at 4 °C. PVDF membranes were then blocked in 5% w/v BSA (in 1× TBST solution for 1 h at RT). The membranes were probed with the primary antibodies for Tim23 or CD81 with gentle shaking at 4 °C overnight in a cold room. Membranes were then washed three times with 1× TBST, and secondary anti-rabbit-HRP was added and incubated with gentle shaking for 1 h at room temperature. Membranes were washed three times for 10 min each prior to chemiluminescent HRP substrate incubation (1 min). Membranes were then imaged using GeneSys software in the SynGene PXi Gel Imaging System.

### ImageStream analysis of sEVs

ImageStream imaging flow cytometry was completed using sEVs stained with CD63 eFlour450 (positive marker to distinguish sEVs from other types of sEVs, cellular debris, and calibration beads), HLA-DR APC, CD54 PE, CD9 PE, and CD81 PE-Cy7 for human studies or MHC-II eFlour450, CD63 APC, CD81 PE, and CD9 PE-Cy7 for murine studies. For PKH26 and CellTrace CFSE experiments, sEVs were labeled with respective dyes following the manufacturer’s protocols. CFSE labeling was stopped with 3% BSA in PBS instead of FBS, which contains exogenous EVs. Stained samples were imaged at 60X magnification without extended depth of field (EDF), while acquiring data on channels Ch01 (Brightfield), Ch03, Ch06, Ch07, Ch09 (Brightfield), Ch11, and Ch12 (side-scatter). Appropriate controls, single color stains, and calibration beads were used to adjust spectral compensation and to calibrate the machine. ApogeeBead Mix (was used to determine the gating of 100 nm sized particles. A total of 5000 events were acquired, gating on forward- and side-scatters, as well as aspect ratio and area. The acquired data was analyzed using IDEAS software version 6.2.

### Flow cytometry analysis of sEVs

A total of 1 × 10^7^ particles of airway sEVs were stained with antibodies specific for CD63 eFluor450, CD54 PE, HLA-DR APC, CD9 PE, CD81 PE-Cy7, and TSG101 Alexa Fluor 647. Flow cytometry data were acquired on a BD Becton Dickinson LSRII configured to detect small particles with ApogeeBead Mix by changing the photomultiplier tube (PMT) voltage for forward and side scatters to 600 volts and 286 volts, respectively, and thresholding on both forward and side scatters to 500 volts. Particles scattering in the same range as beads between 50 nm and 150 nm were gated, and a total of 100,000 events were acquired. PBS with antibodies alone was used as a control to confirm the absence of a background signal due to antibodies in the samples. Fluorescence compensation was configured using single-color controls of sEVs. Acquired flow cytometry data were analyzed using FlowJo X.

### ImageStream and flow cytometry analysis of CD4^+^ T cells

Autologous peripheral CD4^+^ T cells were blocked for 30 min in sEV-depleted RPMI 1640 with 10% human AB serum. Cells were fixed and permeabilized using BD Cytoperm/Cytofix Kit. Cells were then labeled with CD4 PE-Cy7, IL-4 PE, IL-17A APC, IFNγ BV421, CD69 eFluor450, CD154 APC, pZap70 Alexa Fluor 647, and α-tubulin eFluor 615 for 30 min on ice and washed twice. For flow cytometry, cells were resuspended in 100 µl of PBS. For ImageStream, cells were resuspended in 30 µl of PBS in a 1.5 ml microcentrifuge tube. PMTs were adjusted using unstained and compensation configured using single color controls.

### Assessment of NF-kB signaling in sEV-CD4^+^ T co-cultures

Primary human peripheral T cells isolated from healthy and asthmatics (*n* = 5–7/group) were cultured in a sEV-free RPMI with 20% FBS, 2% L-Glutamine, and 1% Penicillin-Streptomycin. For primary T cell co-cultures, recombinant human IL-2 was supplemented at a final concentration of 50 IU/ml. Purified BALF sEVs labeled were treated with 10 µM rotenone, a mitochondrial Complex I inhibitor. sEVs were then washed and purified by centrifugation and then labeled with MitoTracker Green, following which they were washed and purified. Treated and untreated sEVs were then cultured for 24 h with autologous peripheral CD4^+^ T cells at a ratio of 10 sEV per T cell based on concentrations determined from Spectradyne analysis. CD4^+^ T cells were blocked for 30 min in sEV-depleted RPMI media as described above, then labeled with CD4 PE-Cy7 and washed twice with prewarmed FACS buffer. T cells were then fixed (10 min, 37 °C) with pre-warmed BD Cytofix Fixation Buffer, permeabilized (30 min on ice), and washed twice with the FACS Buffer. Cells were then stained with NF-κB p65(pS529) in permeabilization buffer for 30 min at room temperature. Finally, cells were washed twice and resuspended in 100 μl of PBS for flow cytometry. The percentage of CD4^+^ T cells that were MitoTG^+^ and NF-κB p65 (pS529)^+^ was determined from sEV-T cell co-cultures. To assess effects on Th polarization, both treated and untreated sEVs were cultured for 7 days with autologous peripheral CD4^+^ T cells at a ratio of 10 sEVs per T cell. As above, CD4^+^ T cells were labeled with CD4 PE, fixed with BD Cytofix Fixation Buffer, and permeabilized before cells were stained with NF-kB p65(pS529) BV421, IL-17A APC, and IL-4 PerCP-Cy5.5 in permeabilization buffer. Then, cells were washed twice and resuspended in 100 μl of PBS for flow cytometry. The percentages of CD4^+^ T cells that were NF-kB p65 (pS529)^+^ and IL-17A^+^ or IL-4^+^ were determined from these sEV-T cell co-cultures. Controls, including unstained, single color, and FMO controls, were used for flow cytometry acquisition. Acquired flow cytometry data were analyzed using FlowJo 10.9 version.

### Transduction of cells with CellLight constructs

MDRCs or autologous peripheral CD4^+^ T cells were transduced with CellLight Bacman reagents (Mitochondria GFP, Early Endosome RFP, or Lysosome RFP) at a particle-per-cell value of 10 (10 µl) for 1 × 10^5^ cells/well in a 96-well plate, following the manufacturer’s protocols. An exception to this was with the transduction of CD4^+^ T cells with CellLight Tubulin RFP, where the reagent volume was doubled (20 µl) due to lower transduction efficiency. Transduction was performed in sEV-depleted RPMI 1640 with human AB serum for 24 h.

### Labeling sEVs with PKH26

sEVs were labeled with PKH26 (2 µM) in PBS. Samples were stained for 15 min at 37 ˚C in the dark. sEVs were then isolated using the Total Exosome Isolation from Cell Culture Media kit, per the manufacturer’s protocol. The final sEV pellet was resuspended in 50 µl of sterile PBS.

### Labeling cells with PKH26 or MitoView 633

Autologous peripherial CD4^+^ T cells were labeled with PKH26 (2 µM) following the manufacturer’s protocol. Samples were stained for 15 min at 37˚C in the dark, followed by two washes with pre-warmed sEV-deplete RPMI with 10% human AB serum. For MitoView 633 staining, cells were live stained with MitoView 633 (100 nM) in sEV-depleted RPMI 1640 with 10% human AB serum for 15 min at 37˚C in the dark. Cells were then washed twice with pre-warmed sEV-depleted RPMI 1640 with 10% human AB serum.

### Co-culture of sEVs with CD4^+^ T cells and inhibition assays

Purified airway sEVs were cultured with autologous peripheral CD4^+^ T cells at a ratio of 10 sEV per T cell. For sEVs generated from MDRCs, conditions were normalized so that a ratio of 2.5 × 10^5^ MDRCs (sEV-generating cells) to 1 × 10^6^ CD4^+^ T cells was used. sEV-T cell co-culture was performed for 24-h in sEV-depleted media. Mitochondrial inhibitors used to block complex I, complex II, complex III, or complex V were used at the following final concentrations: 10 nM rotenone, 10 µM TTFA, 10 µM antimycin A, 10 µM oligomycin. MitoTEMPOL was used at a concentration of 1 µM. sEVs were treated with the inhibitors 48 h prior to co-culture and purified twice using the Total Exosome Isolation kit to prevent carryover of inhibitors into the co-culture. LFA-1 antibody (1 µg/ml), pan-HLA (DR/DP/DQ, 10 µg/ml), Pitstop2 (50 nM), or Dynasore (50 nM) were used for blocking experiments.

### Cytokine assessment in sEV-CD4^+^ T cell co-culture supernatants or mouse BALF

Co-culture supernatants collected following co-culture of BALF isolated sEVs and autologous peripheral CD4^+^ T cells, as described above, were utilized to measure levels of IL-4 and IL-17 via ELISA immunoassays, following the manufacturer’s recommendations. BALF collected from sensitized and challenged mice following intranasal adoptive transfer of sEVs was utilized to assess Th2 and Th17 cytokines, including IL-4 and IL-17A, via ELISA immunoassays, following the manufacturer’s recommendations.

### NanoString gene expression analysis of CD4^+^ T cells

Autologous peripheral CD4^+^ T cells were cultured with or without sEVs for 7 days in sEV-depleted RPMI supplemented with 10% human AB serum and 50 IU/ml rhIL-2. Cells were harvested and processed for RNA purification using the Invitrogen PureLink RNA Mini Kit. NanoString differential gene expression analysis was conducted using a custom panel of 77 genes at the NanoString core facility at the University of Alabama at Birmingham. Differential gene expression analysis was performed using nSolver version 3 (NanoString, Seattle, WA), R, and Metaboanalyst 3.0 (Xia Lab, McGill University, Montreal, Canada).

### Real-time qPCR analysis of CD4^+^ T cells

Autologous peripheral CD4^+^ T cells were cultured with or without sEVs for 7 days in sEV-depleted RPMI supplemented with 10% human AB serum and 50 IU/ml rhIL-2. Cells were harvested and processed for RNA purification by Trizol or NucleoSpin@RNA plus. Purified RNA was reconstituted in nuclease-free water, and the concentration was quantified. cDNA was synthesized, and qRT-PCR reactions were prepared with 100 ng of cDNA. *ACTB* or *GAPDH* was used as a reference gene. Gene expression of *IL-4*, *IL-17*, *RELA*, *GATA-3*, or *RORC* was measured (primers and sequences listed in Supplementary Table 1). qRT-PCR was performed on a StepOne system. Gene expression data analysis was performed using the 2^-ΔΔCT^ method.

### DNM1L siRNA and qRT-PCR analysis

MDRCs were transfected with ON-TARGETplus SMARTpool *DNM1L* siRNA using Lipofectamine 3000, per the manufacturer’s protocol. Cells were transduced with CellLight Mitochondria GFP and cultured for 48 h in sEV-depleted RPMI supplemented with 10% human AB serum. Supernatants were harvested for sEV isolation and characterization by flow cytometry. Cell pellets were collected for RNA isolation using Trizol, following the manufacturer’s protocol. RNA was quantified, cDNA generated, and qRT-PCR reactions were prepared with 100 ng of cDNA. *DNM1L* gene expression was evaluated, and *ACTB* was used as a reference gene. qRT-PCR was performed on a StepOne system.

### Native-PAGE and Western blot analyses

For human studies, Sorted HLA-DR^+^CD11b^+^ MDRCs from 3 asthmatics and 3 healthy subjects were pooled within each study group. Cells were washed, and cell pellets were resuspended in 50 µl of PBS. For murine studies, lung MDRCs sorted from five control and five mice with asthma were pooled within each study group. Cells were treated with either 1 µM P110 or vehicle (H₂O) and cultured in sEV-depleted medium in 96-well plates for 48 h. Following culture, cells were washed and pelleted in 100 µL of lysis buffer (PBS with 1% *n*-dodecyl β-D-maltoside (DDM). Cell pellets were sonicated on ice five times in short 5 s bursts to minimize heat. Protein was quantitated and samples were diluted in Native PAGE sample buffer. Thirty-five micrograms (human) to forty micrograms (mouse) of sample was loaded on a 4–15% gradient gel. To estimate the molecular weight of proteins, 20 µl of NativeMARK Standard was loaded onto the gel. Using a non-SDS native gel running buffer (1.92 M Glycine, 250 mM Tris-Base, pH 8.3, not adjusted), gels were run at 100 V for 6 h at 4 °C to minimize heat. After electrophoresis, proteins were transferred onto a methanol-activated PVDF membrane using transfer buffer (1.92 M Glycine, 250 mM Tris-Base) overnight at a constant 100 mA at 4 °C. Following transfer, membranes were washed and blocked (human studies: TBS-T with 5% non-fat milk overnight at 4 °C or murine studies: TBS-T containing 4% BSA for 1 h at room temperature). Membranes were probed with HRP-conjugated α-tubulin antibody (1:5000, loading control normalization) for 2 h at room temperature. Membranes were subsequently incubated with mouse anti-DRP1 primary antibody (1:1000) overnight at 4 °C and washed three times in TBS-T. Detection was performed using HRP-conjugated anti-mouse secondary antibody (1:5000) for 2 h at room temperature. Protein bands were visualized using chemiluminescent substrate, and densitometric analysis was performed using ImageJ software (NIH).

### Cytokine and chemokine assay of sEV-treated THP-1 cells

IL-1β, TNF-α, IFN-γ, MCP-1, MIP-1β, MIP-1α, Eotaxin, and VEGF in culture supernatant were assayed by a Luminex magnetic bead-based multiplexed (Bio-Plex 200 system) assay using the commercially available Milliplex MAP Human cytokine/chemokine assay according to the manufacturer’s recommended protocol.

### Lung histology and imaging

Mouse lungs were inflated with 1 ml of 10% formalin and subsequently processed into paraffin blocks following standard procedures. Sections cut at 5 µm were cleared of paraffin using xylene and then taken through a graded ethanol series to rehydrate. Histochemical stainings were performed using commercially available kits for Hematoxylin and eosin (H&E), Masson Trichrome, and periodic acid schiff (PAS). Images were captured utilizing the 10x objective of the Keyence BZ-X10 microscope, and scale bars of 100 µm were placed by automation.

### Isolation of MDRC-derived sEVs from conditioned media

Immunosorted Gr-1^+^CD11b^+^Ly6G^+^F4/80^lo^ MDRCs were cultured in sEV-depleted RPMI 1640 media for 24 h. Conditioned media were collected and centrifuged at 2000×*g* to remove any cell pellets and apoptotic bodies. The supernatant was then incubated with the Total Exosome Isolation Reagent for Cell Culture Media, per the manufacturer’s protocol. Purified sEVs were stored in 50 µl of PBS at −80 °C.

### BALF differential cell count

BALF was collected for Diff-Quik staining from mice after i.n. adoptive transfer of MDRC-derived sEVs. BALF differential analysis was performed following Diff-Quik staining and assessing standard morphological criteria to quantify BALF cells on cytospin slides. At least 300 cells were examined in each cytospin slide. Numbers of eosinophils, macrophages, neutrophils, and lymphocytes were calculated based on the percentage of each cell population in the slides.

### Flow cytometry of murine lung cells

Murine lung tissues were harvested and digested with collagenase B. Red blood cells were lysed via ACK lysis, and cells were blocked with 3% BSA in PBS containing 2.4G2 antibody (anti-mouse CD16/CD32). Cells were then stained with Gr-1 PE, CD25 PE, CD62L PE, CD206 APC, IL-4 APC, Ly6C PerCP-Cy5.5, FoxP3 PerCP-Cy5.5, CD170 PerCP-eFluor 710, MHC-II PE-Cy5, CD4 PE-Cy7, F4/80 BV605, CD11b APC-Cy7, CD3 APC-Cy7, Ly6G Alexa Fluor 700, CD101 Alexa Fluor 647, IL-33Rα PerCP-Cy5.5, IL-17A PE, CD45 PE-Cy7, CD4 PE-Cy7, CD125 PE-Cy7, lingeage cocktail Pacific Blue, CD4 Pacific Blue, CD8a Pacific Blue, CD11c Pacific Blue, NK1.1 Pacific Blue, FcεRIα Pacific Blue, IFN-γ Pacific Blue, CD193 BV421, CD90.2 APC, CD127 APC, CD3 APC-Cy7, or CD278 PE. Data were collected with an LSR-II flow cytometer and analyzed with FlowJo software (version 8.5.2).

### ImageStream analysis of lung tissue cells after sEV transfer

Cells from collagenase-digested lung tissue were prepared for Imagestream flow cytometry as described above. Following preparation of a single cell suspension, cells were treated with Fc Block and then stained with CD45 PE, CD4 PE-Cy7, and CD69 eFlour450. Unstained control, single color staining, and calibration beads were used to calibrate the machine and adjust compensation. The stained samples were imaged at 60 x magnification with EDF. The data were acquired on channels Ch01 (brightfield), Ch03, Ch06, Ch07, Ch09 (brightfield), Ch11 and Ch12 (side-scatter). A total of 5000 events were acquired for each sample, with three technical replicates per sample. The acquired data were analyzed using IDEAS software version 6.2.

### Quantification and statistical analysis

Statistical analysis was performed using GraphPad Prism 5.04, Metaboanalyst 3.0, or R 3.5.2 (64-bit). NanoString data was formatted to be compatible with Metaboanalyst to perform multivariate statistics using Metaboanalyst’s interactive and intuitive platform. Student’s *T* test was utilized for comparison of 2 groups (95% convidence intervals), and ANOVA was utilized to compare 3 or more groups. Descriptions of the statistical tests used are elaborated in the figure legends. Significance was defined as a *p*-value lower than 0.05, and denoted as: ^*^<0.05; ^**^ <0.01; ^***^<0.001; and ^****^<0.0001. Error bars represent standard deviation.

### Reagent and resource sharing

Further information and requests for resources and reagents should be directed to and will be fulfilled by the Lead Contact, Jessy S. Deshane, PhD (treena@uab.edu).

### Reporting summary

Further information on research design is available in the Nature Portfolio Reporting Summary linked to this article.

## Supplementary information

Supplementary Information

Description of Additional Supplementary Files

Supplementary Video 1

Supplementary Video 2

Reporting Summary

Transparent Peer Review file

## Source data

Source Data

## Data Availability

All data are included in the Supplementary Information or available from the authors, as are unique reagents used in this article. The raw numbers for charts and graphs are available in the Source Data file whenever possible. The NanoString custom panel gene expression data have been deposited in the Gene Expression Omnibus (GEO) under accession #GSE144813 and # GPL28122.

## Code availability

MATLAB code is available through GitHub (https://github.com/kgoliwas/Deshane-Lab-Imagestream-co-localization-Matlab-Code).
