# Supplementary material for: Small extracellular vesicle signaling and mitochondrial transfer reprogram T helper cell function in human asthma
Source: Nat Commun. 2026 May 26;17:6842. doi: 10.1038/s41467-026-73684-y (PMC13389491; doi:10.1038/s41467-026-73684-y)
Supplement: Supplementary file 1 — Supplementary Information [file 41467_2026_73684_MOESM1_ESM.pdf]

Supplementary Information

Small Extracellular Vesicle Signaling and Mitochondrial Transfer Reprograms T Helper Cell Function in Human Asthma

Kenneth P. Hough *et. al.*

Corresponding Author: Jessy S. Deshane

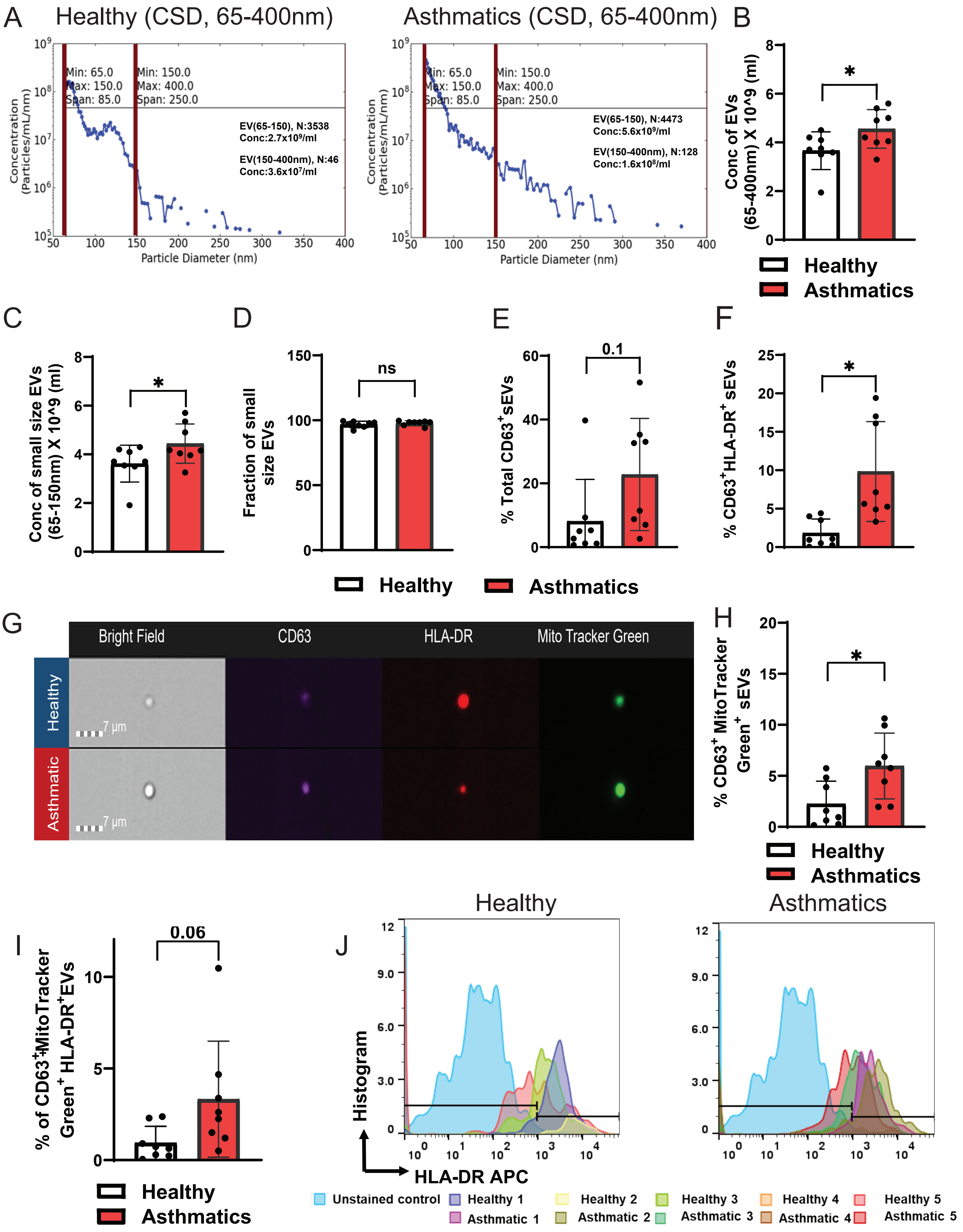

A
Healthy (CSD, 65-400nm)
Asthmatics (CSD, 65-400nm)
Min: 65.0
Max: 150.0
Span: 85.0
Min: 150.0
Max: 400.0
Span: 250.0
EV(65-150), N:3538
Conc:2.7x10⁹/ml
EV(150-400nm), N:46
Conc:3.6x10⁷/ml
EV(65-150), N:4473
Conc:5.6x10⁹/ml
EV(150-400nm), N:128
Conc:1.6x10⁸/ml
Concentration (Particles/mL/nm)
Particle Diameter (nm)
B
Conc of EVs (65-400nm) X 10^9 (ml)
Healthy
Asthmatics
C
Conc of small size EVs (65-150nm) X 10^9 (ml)
D
Fraction of small size EVs
ns
E
% Total CD63⁺sEVs
0.1
F
% CD63⁺HLA-DR⁺ sEVs
G
Bright Field
CD63
HLA-DR
Mito Tracker Green
Healthy
Asthmatic
7 µm
H
% CD63⁺ MitoTracker Green⁺ sEVs
I
% of CD63⁺MitoTracker Green⁺HLA-DR⁺EVs
0.06
J
Healthy
Asthmatics
Histogram
HLA-DR APC
Unstained control
Healthy 1
Healthy 2
Healthy 3
Healthy 4
Healthy 5
Asthmatic 1
Asthmatic 2
Asthmatic 3
Asthmatic 4
Asthmatic 5

Supplementary Figure 1 – BALF CD63$^+$ sEVs and MitoTrackerGreen$^+$ sEVs within these sEVs have Class II expression. BALF sEVs isolated from Healthy controls and Asthmatics were assessed by Spectradyne's nCS1^{TM} Particle Analyzer that delivers accurate nanoparticle size and concentration. (A) Representative acquired CSD image of quantification of BALF sEVs, Red Box highlights the sEV gate that is quantitated in B-D. (B) Concentration of all BALF EVs comparing Healthy controls and Asthmatics (n=8). (C) Concentration of BALF sEVs comparing Healthy controls and Asthmatics (n=8).(D) Percent sEVs of total BALF EVs comparing Healthy controls and Asthmatics (n=8). (E) Percent total CD63$^+$ sEVs isolated from BALF comparing Healthy Controls and Asthmatics (n=8). (F) Percent of CD63$^+$ HLA-DR$^+$ sEVs from BALF comparing Healthy controls and Asthmatics (n=8). (G) Representative image strips from ImageStream analyses showing HLA-DR$^+$ and MitoTracker Green$^+$ BALF sEVs in Healthy controls and Asthmatics. (H) Quantitation of % CD63$^+$ MitoTracker Green$^+$ BALF sEVs by ImageStream (n=8). (I) Quantitation of % CD63$^+$ MitoTrackerGreen$^+$ HLA-DR$^+$ BALF sEVs by ImageStream (n=8). (J) Overlaid Histograms of CD63$^+$ MitoTracker Green$^+$ gated BALF sEVs showing HLA-DR expression in Healthy controls and Asthmatics (n=5). Mann Whitney T test was utilized for comparison between Controls and Asthma groups, $^*p<0.05$. Individual data points presented with each bar representing mean ± SD. Source data are provided as a Source Data file.

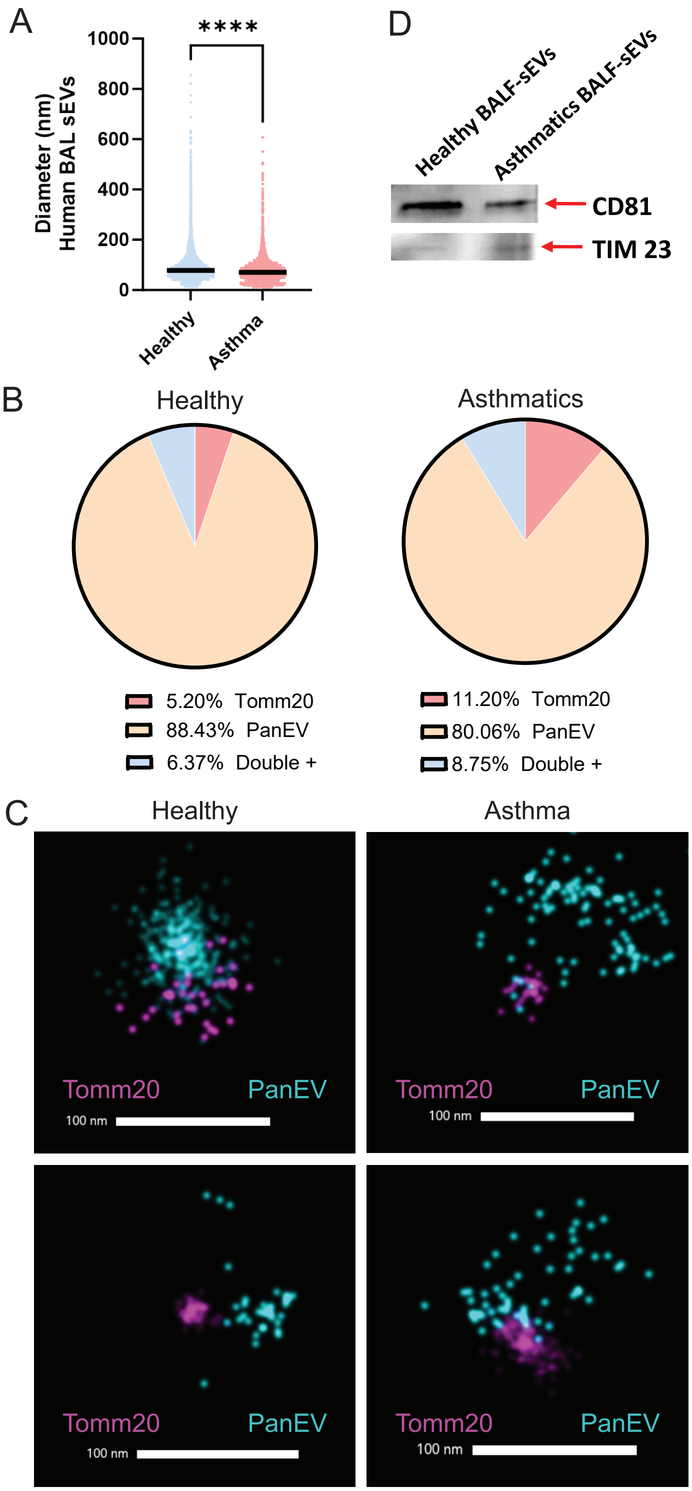

Supplementary Figure 2 – Human BALF- sEVs express PanEV markers and Tomm20. Purified BALF-sEVs from Healthy human controls and Asthmatics were captured and stained using the Oxford Nanoimaging (ONi) EV Profiler Kit 2. Imaging was completed using the AutoEV function on the ONi Nanoimager and utilized direct stochastic optical reconstruction microscopy for high resolution images. All analyses were completed in the CODI software. (A) Quantitation of diameter of BALF-sEVs from Healthy controls and Asthmatics (n=7567 sEVs for Healthy controls and n=6227 sEVs for Asthmatics). Kolmogorov Smirnov test for comparison between Healthy controls and Asthmatics, ****p<0.001. Individual data points presented with bar at mean. (B) Pie charts showing % of sEVs expressing Tomm20 and Pan EV markers in samples from A, determined by Oni image analyses of 10000 events collected for n=3 replicates from pooled BALF-sEVs isolated from n=6/group of Healthy controls and Asthmatics. (C) Representative high-resolution images from nanoimager showing sEVs expressing Tomm 20 and PanEV markers in BALF-sEVs from Heathy controls and Asthmatics. Scale bar = 100 nm. (D) Western Blot Analyses of BALF sEVs from Healthy and Asthmatics isolated as above, lysed and electrophoresed on SDS PAGE and probed with anti-CD81 and anti-TIM23 antibody. Western Blot shows expression of CD81 and TIM 23. Source data are provided as a Source Data file.

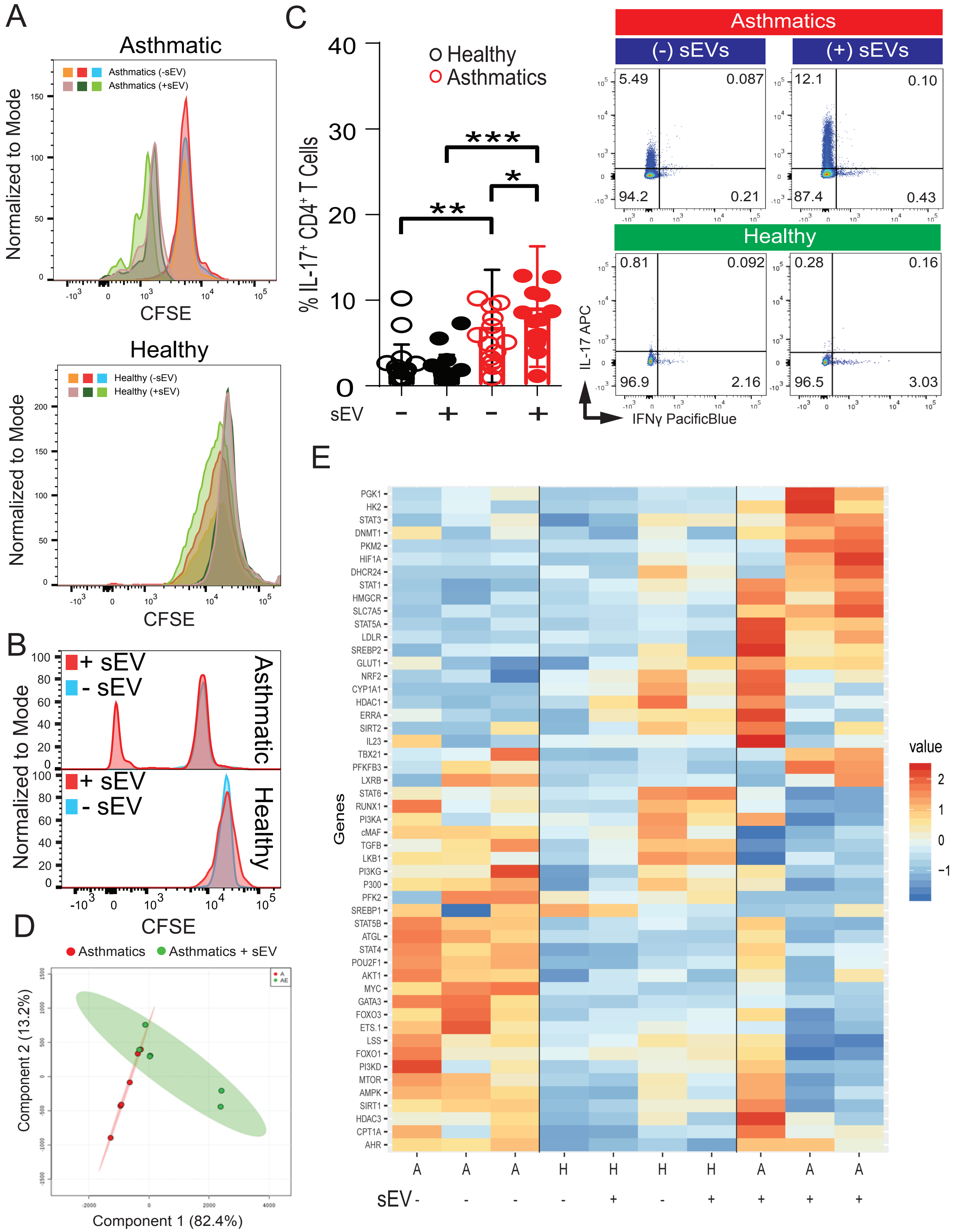

A
Asthmatic
Asthmatics (-sEV)
Asthmatics (+sEV)
Normalized to Mode
CFSE
Healthy
Healthy (-sEV)
Healthy (+sEV)
B
+ sEV
- sEV
Asthmatic
Healthy
C
Healthy
Asthmatics
% IL-17+ CD4+ T Cells
sEV
Asthmatics
(-) sEVs
(+) sEVs
5.49
0.087
94.2
0.21
12.1
0.10
87.4
0.43
Healthy
0.81
0.092
96.9
2.16
0.28
0.16
96.5
3.03
IL-17 APC
IFNγ PacificBlue
D
Asthmatics
Asthmatics + sEV
Component 2 (13.2%)
Component 1 (82.4%)
E
Genes
value
sEV

Supplementary Figure 3 – BALF sEVs from the airways of asthmatics promote proliferation and activation of $CD4^+$ T cells. (A-D) Peripheral and airway $CD4^+$ T cells cultured with and without autologous BALF sEVs for 7 days in the presence of 50 IU/ml rhIL-2 in RPMI supplemented with 10% sEV depleted human AB serum. (A) Representative histogram of peripheral $CD4^+$ T cell CFSE dilution in the –sEV and +sEV groups following culture illustrating proliferation displayed for each group as an overlay. (B) Representative histogram of airway $CD4^+$ T cell CFSE dilution illustrating proliferation displayed for each group as an overlay. (C) sEVs from the BALF fluid were co-cultured with autologous peripheral human $CD4^+$ T cells and percent Th17 subsets analyzed (n=18 for Healthy Controls, n=15 for Asthmatics). Left, percent of Th17 cells (assessed by IL-17 expression), and representative flow plots shown on the right. $CD4^+$ T cells were gated on IL-4^{neg} and then plotted IL-17 versus IFNγ. Mixed Effect ANOVA with Sidak's multiple comparison, **p<0.01, ****p<0.0001. (D) PCA analysis was conducted on gene expression data obtained from NanoString (n=5). PCA analysis was performed using R and MetaboAnalyst 3.0. (E) Heatmap of gene expression data obtained from NanoString. Hierarchical clustering and heatmap was generated using R (libraries used: ggplot2, reshape2, ggdendro, grid; A=Asthmatic, H=Healthy Normal; n=2-3 per group). Individual data points presented with each bar representing mean ± SD. Source data are provided as a Source Data file.

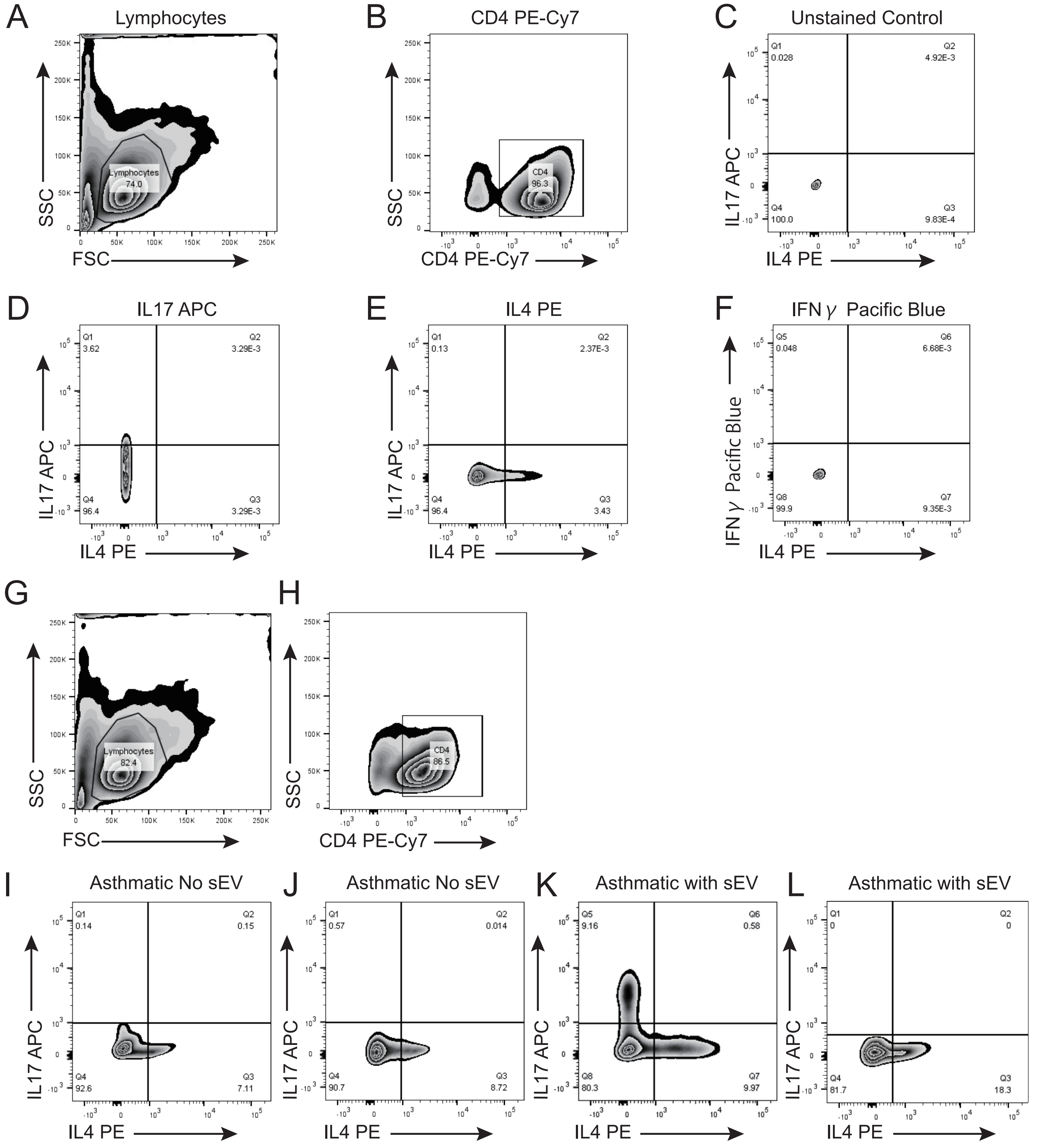

Supplementary Figure 4 – Gating Strategy and Flow Cytometry Controls for T cells from Healthy controls and Asthmatics co-cultured with autologous BALF-EVs and polarized to Th17 and Th2 subsets. (A-B) Cells were gated initially for Lymphocytes (A) followed by CD4 gate (B), (C-F) Unstained and Single color controls, (G-M) Gating strategy for sEV-co-cultured and polarized Th2 and Th17 cells from Healthy controls and Asthmatics as described above, (G-H) Lymphocyte gate (G) followed by CD4 gate (H), (I) $CD4^{+}$ cells were gated for $IFN\gamma^{neg}$ cells, (J-M) Representative flow plots for gates for Th2 and Th17 cells polarized in co-cultures of autologous peripheral T cells in presence or absence of BALF sEVs from Healthy controls and Asthmatics.

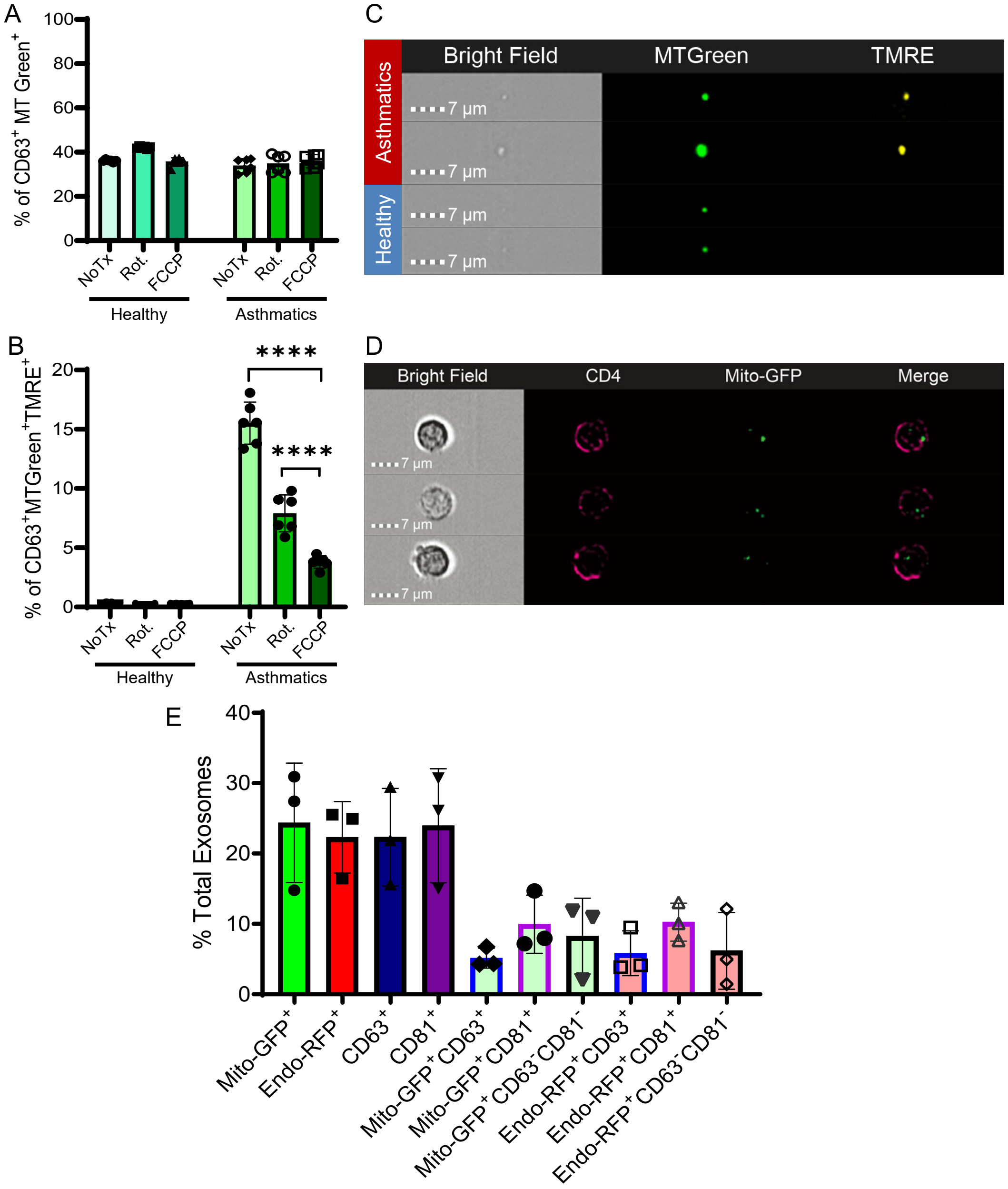

Supplementary Figure 5 – Mitochondrial membrane potential was observed only in MDRC-derived sEVs from asthmatics. (A-C) Airway MDRCs were labeled with MitoTracker Green and sEVs purified from the conditioned media 48 hours later. Purified MDRC sEVs were labeled with TMRE and analyzed on the ImageStream flow cytometer. (A) The % of MitoTracker Green MDRC sEVs that are $CD63^+$. (B) The % of TMRE+ MDRC sEVs that are also positive for CD63 and MitoTracker Green. (C) Representative image strip of MDRC-derived sEVs analyzed by Image-Stream. Mixed Effect ANOVA with Sidak's multiple comparisons test, n=6 per comparison, ****$p<0.0001$. (D-E) MDRC-derived sEVs contain mitochondria and are internalized by autologous peripheral $CD4^+$ T cells. (D) Representative image strip of peripheral $CD4^+$ T cells internalizing Mito-GFP^+ sEVs generated from MDRCs. (E) Characterization of sEVs from MDRCs that were transduced with CellLight Mito-GFP, CellLight Endo-RFP. Transduced MDRCs were cultured for 48 hours before isolation of sEVs from the conditioned media. MDRC sEVs were probed with anti-CD63 and anti-CD81 and characterized on ImageStream flow cytometry. n=3 per comparison. Individual data points presented with each bar representing mean ± SD. Source data are provided as a Source Data file.

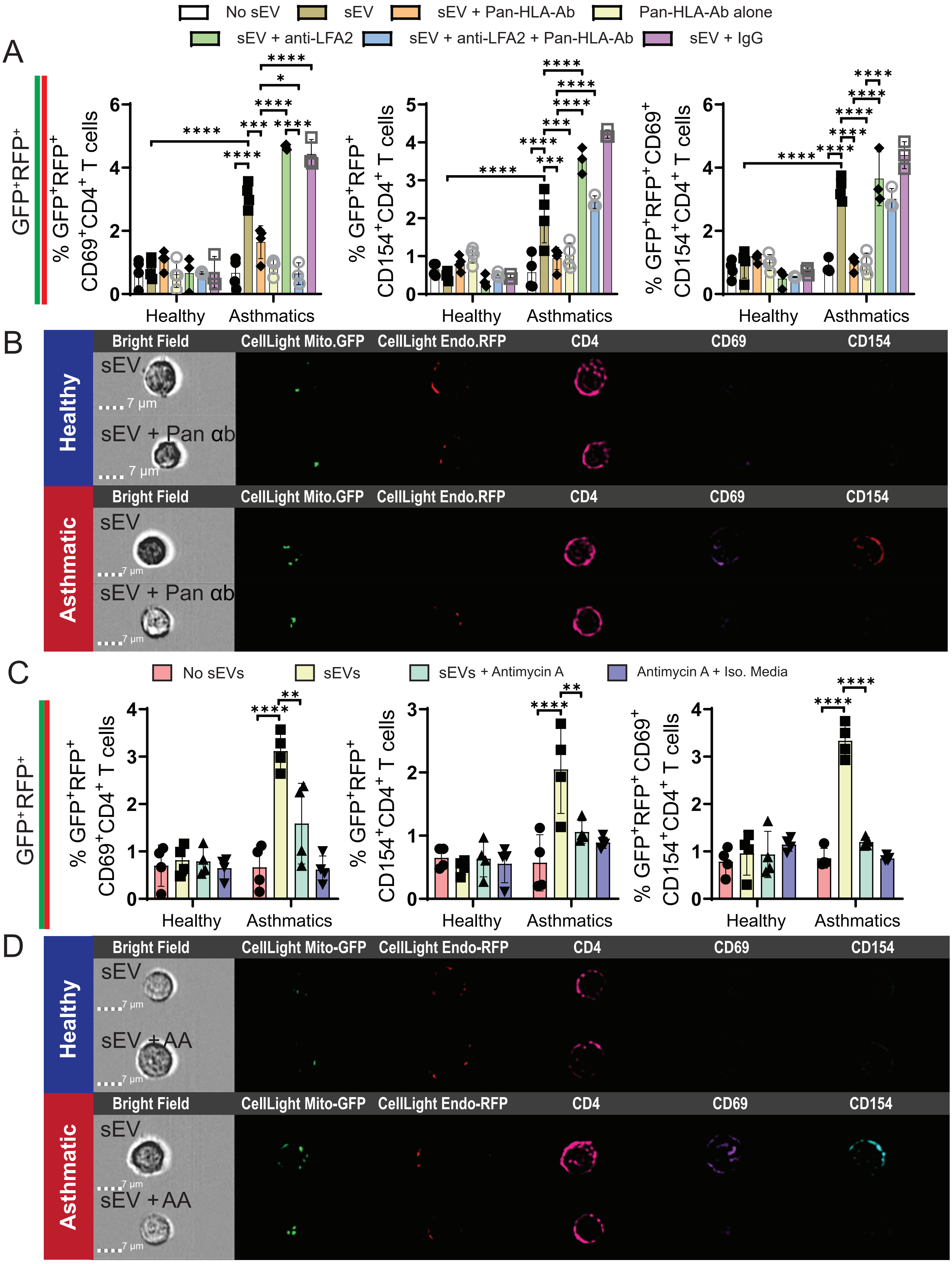
No sEV
sEV
sEV + Pan-HLA-Ab
Pan-HLA-Ab alone
sEV + anti-LFA2
sEV + anti-LFA2 + Pan-HLA-Ab
sEV + IgG
A
GFP+RFP+
% GFP+RFP+ CD69+CD4+ T cells
% GFP+RFP+ CD154+CD4+ T cells
% GFP+RFP+CD69+ CD154+CD4+ T cells
Healthy
Asthmatics
B
Bright Field
CellLight Mito.GFP
CellLight Endo.RFP
CD4
CD69
CD154
Healthy
Asthmatic
sEV
sEV + Pan αb
7 µm
C
No sEVs
sEVs
sEVs + Antimycin A
Antimycin A + Iso. Media
D
CellLight Mito-GFP
CellLight Endo-RFP
sEV + AA

Supplementary Figure 6 - Blockade of class II molecules using a pan-HLA antibody (HLA-DR/DP/DQ) results in loss of MDRC sEV mediated activation of autologous peripheral $CD4^+$ T cells. (A) Graphs illustrating the percentage of $CD69^+$, $CD154^+$, or $CD69^+CD154^+$ T cells that internalized Mito-GFP^+ Endo-RFP^+ MDRC sEVs. Two-way ANOVA, Tukey's multiple comparisons test, n=3-4, Mean of 3 replicates for each sample is represented. *p<0.05, **p<0.01,***p<0.001, ****p<0.0001. (B) Representative image strips from ImageStream illustrating that Mito-GFP^+ Endo-RFP^+ MDRC sEVs activate T cells in asthmatics, while treatment with pan-HLA-Ab abrogates this activation by MDRC sEVs. (C-D) Inhibition of complex III by antimycin A in MDRC sEVs diminishes autologous peripheral $CD4^+$ T cell activation in asthmatics. Results for $CD4^+$ T cells that have internalized both Mito-GFP^+ MDRC sEVs and Mito-RFP^+ MDRC sEVs. (C) Graphs illustrating the percentage of $CD69^+$, $CD154^+$, or $CD69^+CD154^+$ T cells that internalized Mito-GFP^+ Endo-RFP^+ MDRC sEVs. Two way ANOVA, Tukey's multiple comparisons test, n=4, Mean of 3 replicates for each sample is represented. **p<0.01, ***p<0.001, ****p<0.0001. (D) Representative image strips from ImageStream analysis illustrating that Mito-GFP^+ Endo-RFP^+ MDRC sEVs activate T cells in asthmatics, and antimycin blocks activation in asthmatics. Individual data points presented with each bar representing mean ± SD. Source data are provided as a Source Data file.

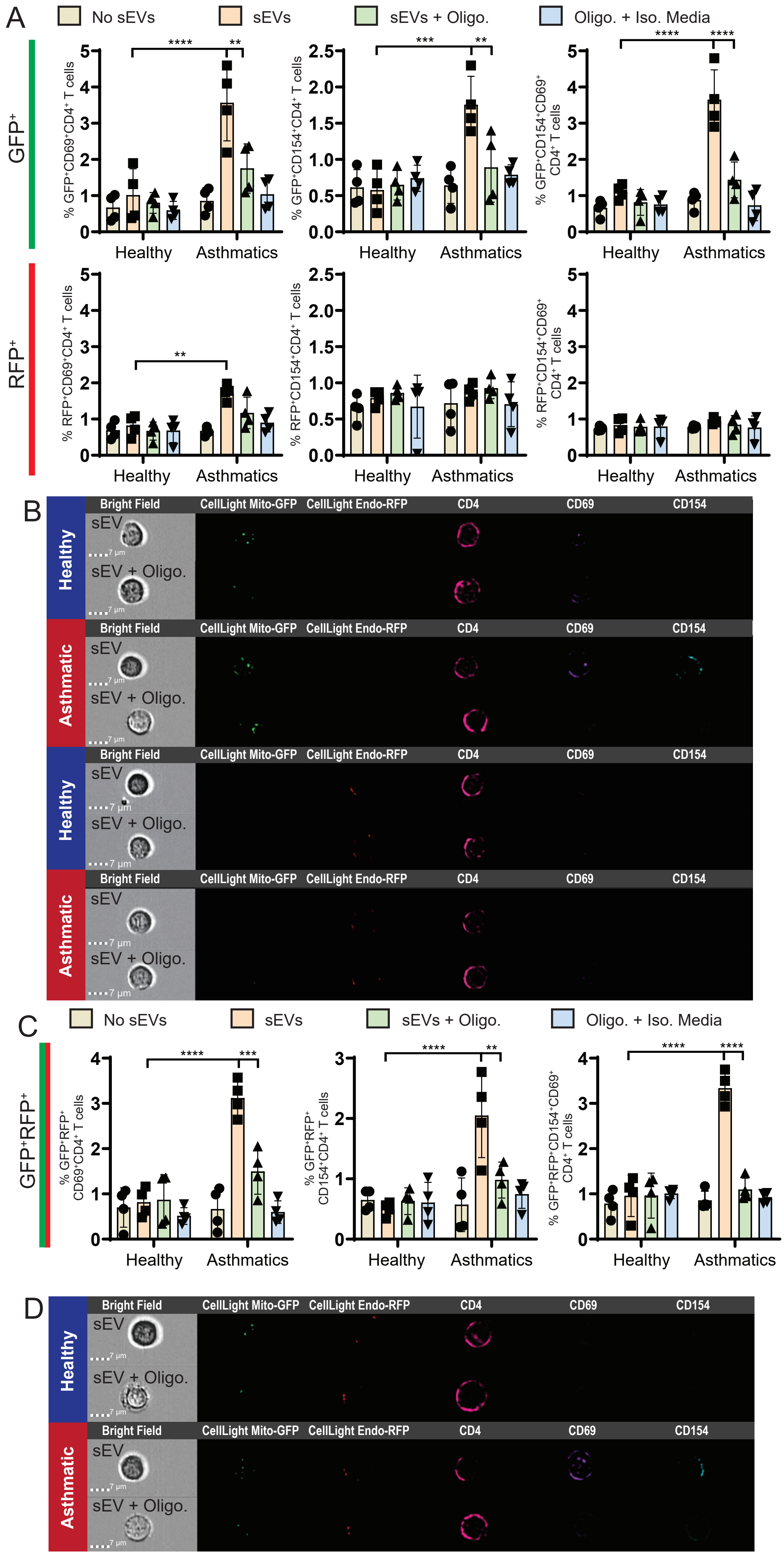

A
No sEVs
sEVs
sEVs + Oligo.
Oligo. + Iso. Media
GFP+
RFP+
% GFP+CD69+CD4+ T cells
% GFP+CD154+CD4+ T cells
% GFP+CD154+CD69+ CD4+ T cells
% RFP+CD69+CD4+ T cells
% RFP+CD154+CD4+ T cells
% RFP+CD154+CD69+ CD4+ T cells
Healthy
Asthmatics
B
Bright Field
CellLight Mito-GFP
CellLight Endo-RFP
CD4
CD69
CD154
Healthy
Asthmatic
sEV
sEV + Oligo.
7 µm
C
GFP+RFP+
% GFP+RFP+ CD69+CD4+ T cells
% GFP+RFP+ CD154+CD4+ T cells
% GFP+RFP+CD154+CD69+ CD4+ T cells
D

Supplementary Figure 7 – Inhibition of complex V by oligomycin in MDRC sEVs diminishes autologous peripheral $CD4^+$ T cell activation in asthmatics. MDRCs were transduced with CellLight Mito-GFP and CellLight Endo-RFP, and sEVs were purified from the supernatant 48 hours later. Purified MDRC sEVs were co-cultured with autologous peripheral $CD4^+$ T cells for 24 hours in the presence of rhIL-2 (50 IU/ml) in a ration of 1:10 T cells:sEVs. MDRC sEVs were pre-treated with oligomycin (10 μM) overnight or untreated prior to co-culture with T cells. sEVs were washed and re-purified using the Invitrogen Total Exosome Isolation kit. ImageStream flow cytometry was used to assess early activation (CD69) and antigen-specific activation (CD154). (A) Graphs illustrating the percentage of $CD69^+$, $CD154^+$, or $CD69^+CD154^+$ T cells that internalized either Mito-GFP^+ MDRC sEVs (top row) or Endo-RFP^+ MDRC sEVs (bottom row). Two way ANOVA, Tukey's multiple comparisons test, n=4, Mean of 3 replicates for each sample is represented. $^{**}p<0.01$,$^{***}p<0.001$, $^{****}p<0.0001$. (B) Representative image strips from ImageStream analysis illustrating that Mito-GFP^+ MDRC sEVs activate T cells in asthmatics, and oligomycin blocks activation in asthmatics. (C) Graphs illustrating the percentage of $CD69^+$, $CD154^+$, or $CD69^+CD154^+$ T cells that internalized either Mito-GFP^+ Endo-RFP^+ MDRC sEVs. Two way ANOVA, Tukey's multiple comparisons test, n=4, Mean of 3 replicates for each sample is represented, $^{**}p<0.01$, $^{***}p<0.001$, $^{****}p<0.0001$. (D) Representative image strips from ImageStream analysis illustrating that Mito-GFP^+ Endo-RFP^+ MDRC sEVs activate T cells in asthmatics, and oligomycin blocks activation in asthmatics. Individual data points presented with each bar representing mean ± SD. Source data are provided as a Source Data file.

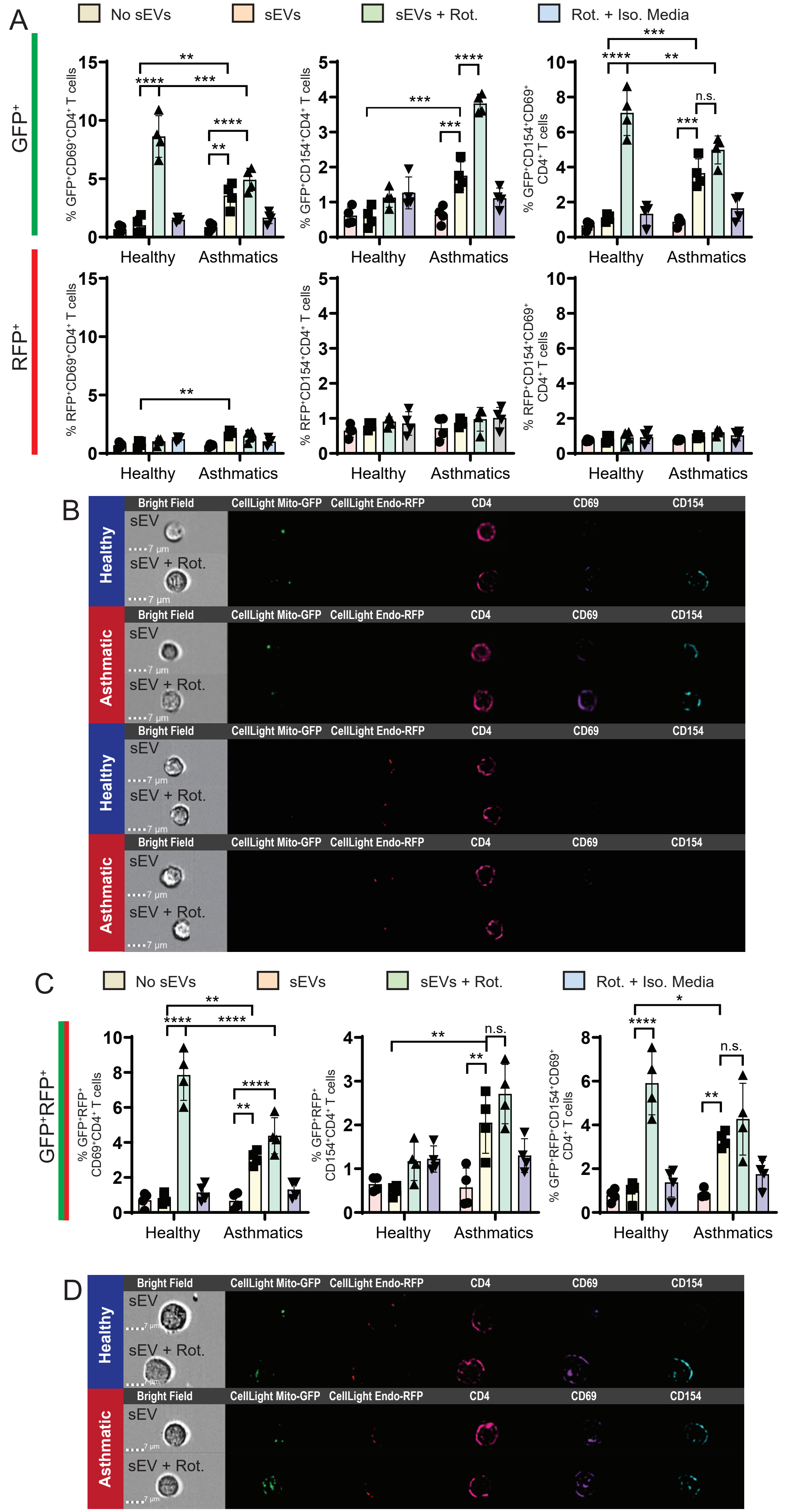
A
No sEVs
sEVs
sEVs + Rot.
Rot. + Iso. Media
GFP⁺
RFP⁺
% GFP⁺CD69⁺CD4⁺ T cells
% GFP⁺CD154⁺CD4⁺ T cells
% GFP⁺CD154⁺CD69⁺ CD4⁺ T cells
% RFP⁺CD69⁺CD4⁺ T cells
% RFP⁺CD154⁺CD4⁺ T cells
% RFP⁺CD154⁺CD69⁺ CD4⁺ T cells
Healthy
Asthmatics
n.s.
B
Healthy
Asthmatic
Bright Field
CellLight Mito-GFP
CellLight Endo-RFP
CD4
CD69
CD154
sEV
sEV + Rot.
7 µm
C
GFP⁺RFP⁺
% GFP⁺RFP⁺ CD69⁺CD4⁺ T cells
% GFP⁺RFP⁺ CD154⁺CD4⁺ T cells
% GFP⁺RFP⁺CD154⁺CD69⁺ CD4⁺ T cells
D

Supplementary Figure 8 – Inhibition of complex I by rotenone in sEVs enhances autologous peripheral $CD4^+$ T cell activation in both healthy and asthmatics. MDRCs were transduced with CellLight Mito-GFP and CellLight Endo-RFP, and sEVs were purified from the supernatant 48 hours later. Purified MDRC sEVs were co-cultured with autologous peripheral $CD4^+$ T cells for 24 hours in the presence of rhIL-2 (50 IU/ml) in a ration of 1:10 T cells:sEVs. MDRC sEVs were pre-treated with rotenone (10 μM) overnight or untreated prior to co-culture with T cells. sEVs were washed and re-purified using the Invitrogen Total Exosome Isolation kit. ImageStream flow cytometry was used to assess early activation (CD69) and antigen-specific activation (CD154). (A) Graphs illustrating the percentage of $CD69^+$, $CD154^+$, or $CD69^+CD154^+$ T cells that internalized either Mito-GFP^+ MDRC sEVs (top row) or Endo-RFP^+ MDRC sEVs (bottom row). Mixed Effect ANOVA with Sidak's multiple comparison's test, n=4, Mean of three replicates per sample are represented. **p<0.01, ***p<0.001, ****p<0.0001. (B) Representative image strips from ImageStream analysis illustrating that Mito-GFP^+ MDRC sEVs activate T cells in asthmatics, and rotenone promotes activation in T cells from healthy and asthmatic subjects. (C) Graphs illustrating the percentage of $CD69^+$, $CD154^+$, or $CD69^+CD154^+$ T cells that internalized Mito-GFP^+ Endo-RFP^+ MDRC sEVs. Two way ANOVA, Tukey's multiple comparisons test, n=4, Mean of 3 replicates for each sample is represented, **p<0.01, ***p<0.001, ****p<0.0001. (D) Representative image strips from ImageStream analysis illustrating that Mito-GFP^+ Endo-RFP^+ MDRC sEVs activate T cells in asthmatics, and rotenone promotes activation in healthy and asthmatic T cells. Individual data points presented with each bar representing mean ± SD. Source data are provided as a Source Data file.

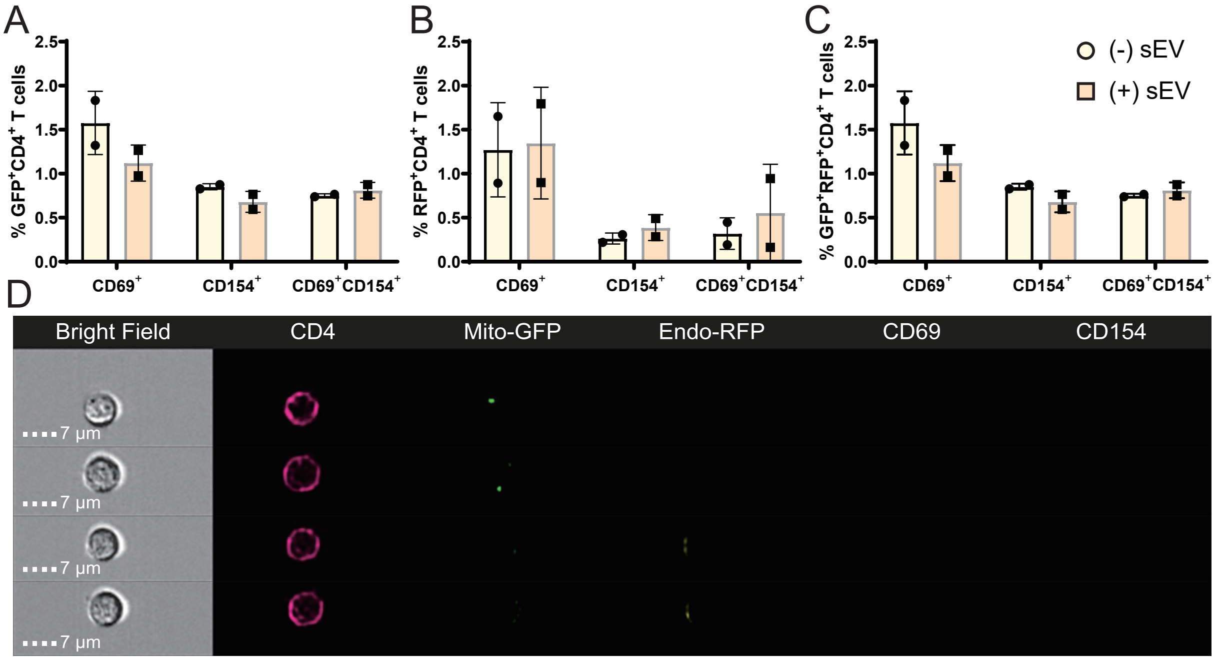

Supplementary Figure 9 – sEVs derived from CD45 negative cells did not activate autologous peripheral CD4⁺ T cells. CD45^{neg} non-immune cells were transduced with CellLight Mito-GFP and CellLight Endo-RFP. Transduced cells were cultured for 48 hours and then sEVs isolated from the conditioned media. Purified CD45^{neg} sEVs were cocultured with autologous peripheral CD4^{+} T cells for 24 hours in the presence of rhIL-2 (50 IU/ml) in a ration of 1:10 T cells:sEVs. ImageStream flow cytometry was used to assess early activation (CD69) and antigen-specific activation (CD154). (A-C) Graphs illustrating the percentage of CD69^{+}, CD154^{+}, or CD69^{+}CD154^{+} T cells that internalized either (A) Mito-GFP^{+} sEVs, (B) Endo-RFP^{+} sEVs, or (C) both Mito-GFP^{+} and Endo-RFP^{+} sEVs, n=2. Individual data points presented with each bar representing mean ± SD. (D) Representative image strips from ImageStream analysis illustrating that activation is not observed despite internalization of nonimmune cell-derived Mito-GFP^{+} sEVs by CD4^{+} T cells. Source data are provided as a Source Data file.

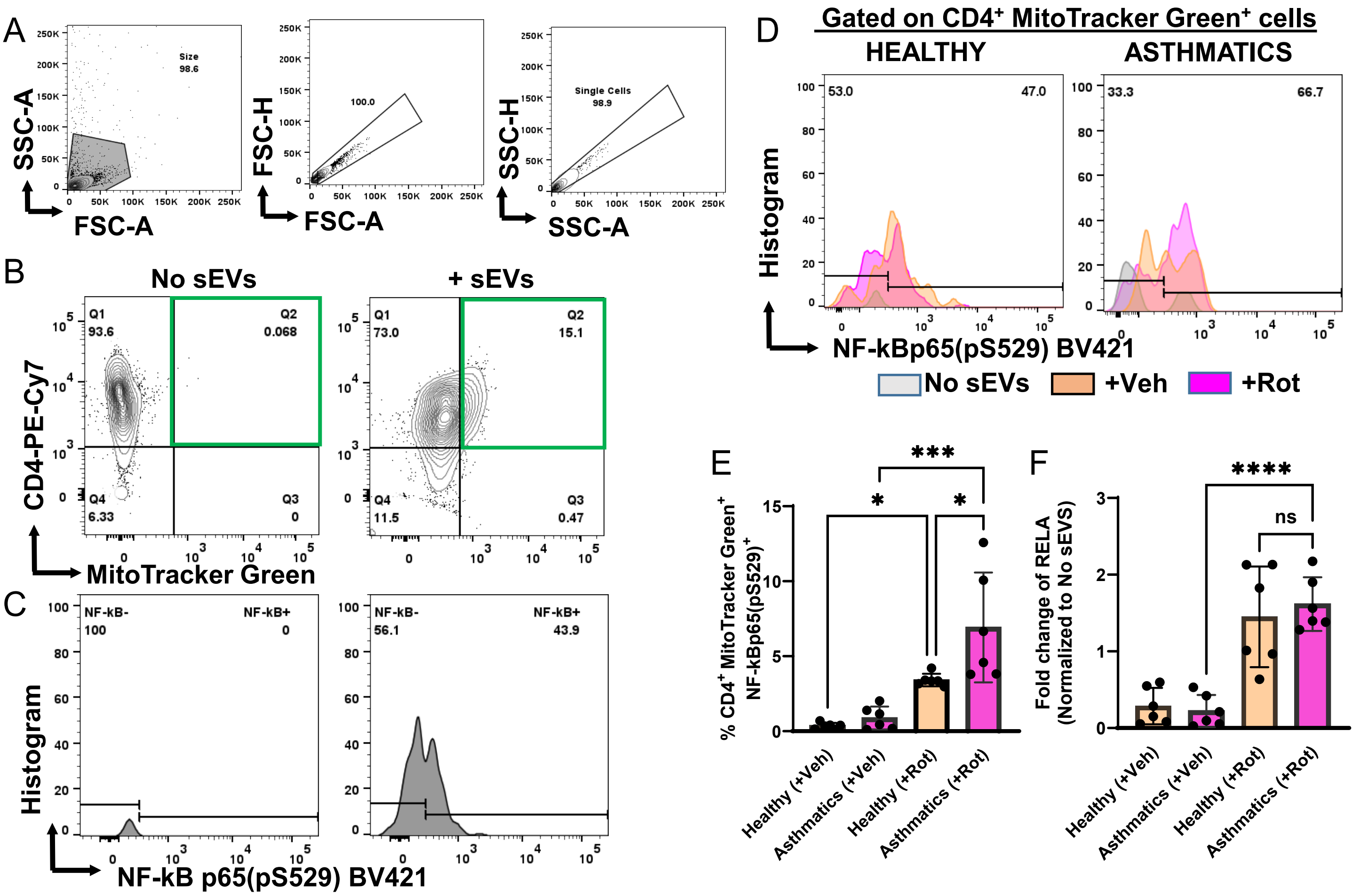

Supplementary Figure 10 – NF-κB signaling in CD4 T cells is enhanced upon co-culture with Human BALF sEVs treated with rotenone, a Complex I inhibitor. Purified human BALF sEVs from healthy and asthmatic subjects were treated with 10 μM rotenone or vehicle control, washed, purified and labeled with MitoTracker-Green. Autologous $CD4^+$ T cells were co-cultured with these sEVs at a 10 sEV: 1 T cell ratio and 24 hours later assessed for % NF-κB p65 $(pS529)^+$ cells within the $CD4^+MitoTrackerGreen^+$ lymphocytes. (A) Gating strategy for ssc-fsc and singlets. (B) Gating Strategy for $CD4^+MitoTrackerGreen^+$ lymphocytes. (C) Gating strategy for NF-κB p65 $(pS529)^+$ cells. (D) Overlaid histogram of NF-κB p65 $(pS529)^+$ cells in CD4 T cells co-cultured with no sEVs, vehicle treated sEVs and rotenone treated sEVs for 24 hours in healthy (n=6) and asthmatic (n=6) subjects. (E) Quantitation of % $CD4^+MItoTrackerGreen^+$ cells that are NF-κB p65 $(pS529)^+$ from (D). (F) Fold change in RELA expression in peripheral $CD4^+$ T cells from healthy (n=6) and asthmatic (n=6) subjects following co-culture with vehicle treated and rotenone treated MitoTracker Green labeled sEVs. Data were normalized to GAPDH and no sEV controls. Gene Data analysis was performed using the 2-ΔΔCT method. One way ANOVA analyses with multiple comparison between treatments and study groups, **p<0.001, ***p=0.0001****p<0.0001. Individual data points presented with each bar representing mean ± SD. Source data are provided as a Source Data file.

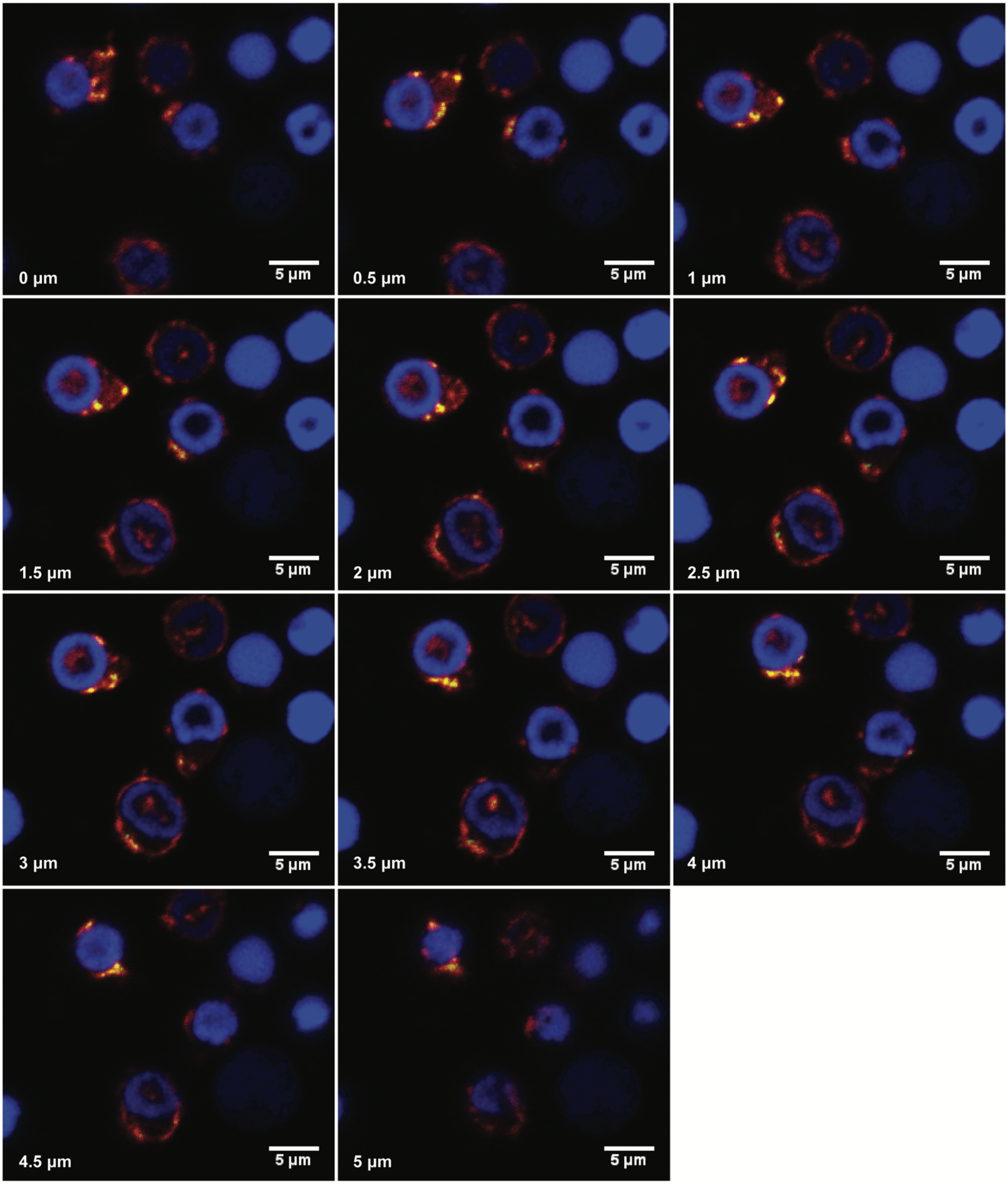

Supplementary Figure 11 – Internalized Mito-GFP⁺ MDRC sEVs co-localizes with cytosolic actin in T cells. Each panel represents a 0.5 μm slice in depth (z-stack, slice depth listed in left corner of each image). Confocal image illustrating internalization of MDRC sEVs inside the cytoplasm of the T cells. MDRCs were transduced with CellLight Mito-GFP and sEVs were purified from the supernatant 48 hours later. Mito-GFP⁺ MDRC sEVs were co-cultured with T cells (1:10 T cell:sEV) for 24-hours in the ibidi μ-dish. Cells were fixed with 2% PFA and permeabilized with 0.5% triton-X in PBS. The fixed T cells were labeled with Phalloidine-Rhodamine and DAPI. Cells were imaged using a Nikon A1 confocal. Scale bar = 5 μm.

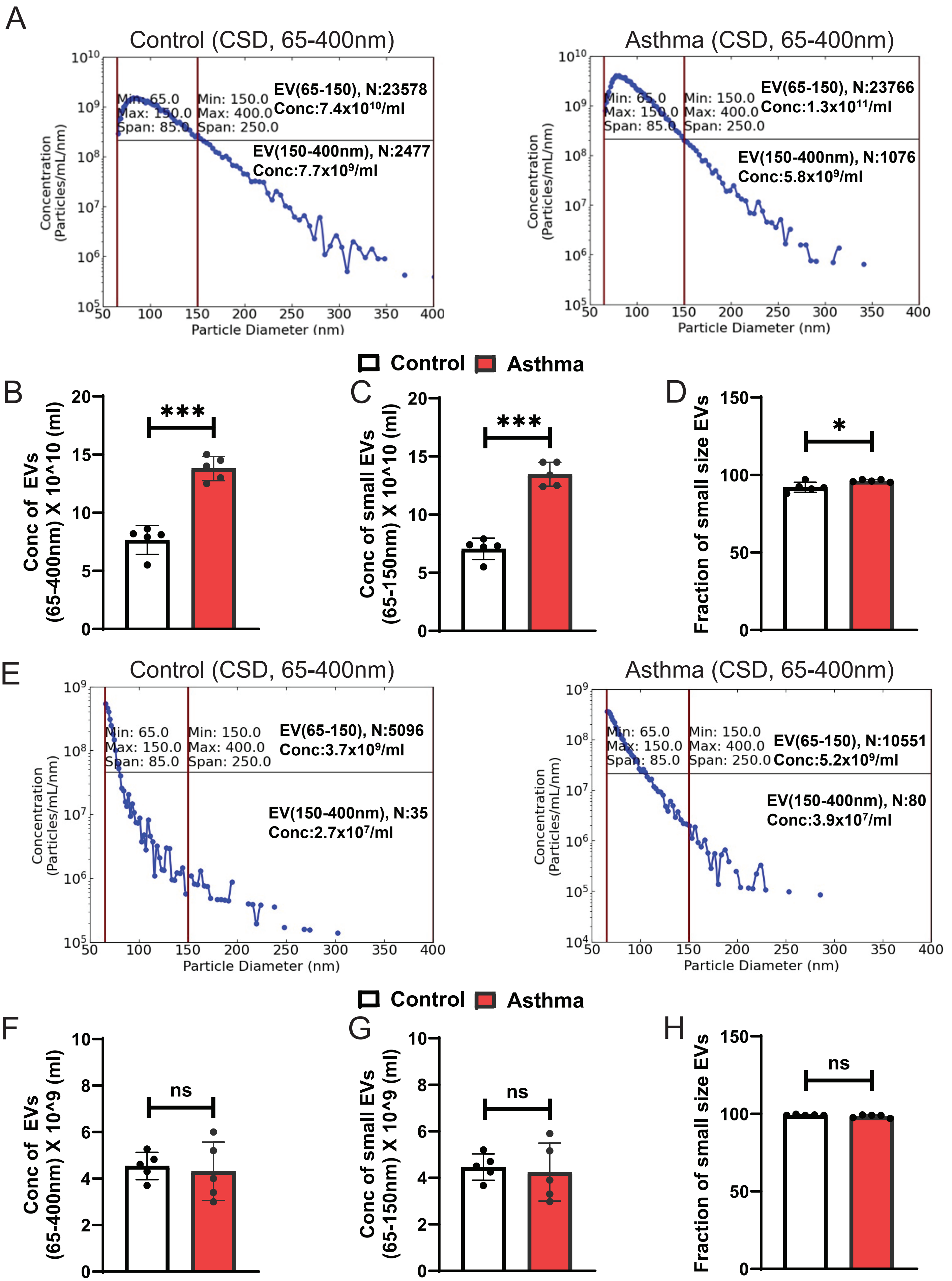

A
Control (CSD, 65-400nm)
Asthma (CSD, 65-400nm)
EV(65-150), N:23578
Conc:7.4x10^10/ml
EV(150-400nm), N:2477
Conc:7.7x10^9/ml
EV(65-150), N:23766
Conc:1.3x10^11/ml
EV(150-400nm), N:1076
Conc:5.8x10^9/ml
Min: 65.0
Max: 150.0
Span: 85.0
Min: 150.0
Max: 400.0
Span: 250.0
Concentration (Particles/mL/nm)
Particle Diameter (nm)
Control
Asthma
B
C
D
Conc of EVs (65-400nm) X 10^10 (ml)
Conc of small EVs (65-150nm) X 10^10 (ml)
Fraction of small size EVs
***
*
E
EV(65-150), N:5096
Conc:3.7x10^9/ml
EV(150-400nm), N:35
Conc:2.7x10^7/ml
EV(65-150), N:10551
Conc:5.2x10^9/ml
EV(150-400nm), N:80
Conc:3.9x10^7/ml
F
G
H
Conc of EVs (65-400nm) X 10^9 (ml)
Conc of small EVs (65-150nm) X 10^9 (ml)
ns

Supplementary Figure 12 – Murine BALF EVs and MDRC-derived EVs are small size EVs. (A) Representative acquired CSD images of quantitation by Spectradyne's nCS1 nanoparticle analyzer of BALF sEVs isolated from Mito-QC mice sensitized by OVA and challenged with PBS (Control) and from Mito-QC mice sensitized and challenged with OVA (Asthma), Red Box highlights the sEV gate that is quantitated in B-D. (B) Concentration of all BALF EVs comparing Control and Asthma groups. (C) Concentration of BALF sEVs comparing Control and Asthma groups. (D) Percent sEVs of total BALF EVs comparing Control and Asthma. (E) Representative acquired CSD images of quantitation by Spectradyne's nCS1 nanoparticle analyzer of MDRC sEVs isolated from MitoQC mice sensitized by OVA and challenged with PBS (Control) and from MitoQC mice sensitized and challenged with OVA (Asthma), Red Box highlights the sEV gate that is quantitated in F-H. (F) Concentration of all MDRC EVs comparing Control and Asthma groups. (G) Concentration of MDRC sEVs comparing Control and Asthma groups. (H) Percent sEVs of total MDRC EVs comparing Control and Asthma. EVs were isolated from n=6 mice/group from BALF and MDRCs and pooled to obtain sEVs for each data point. Data represents 5 replicate experiments. Mann Whitney T test for comparison between Controls and Asthma groups, $*p<0.05$, $****p<0.001$. Individual data points presented with each bar representing mean ± SD. Source data are provided as a Source Data file.

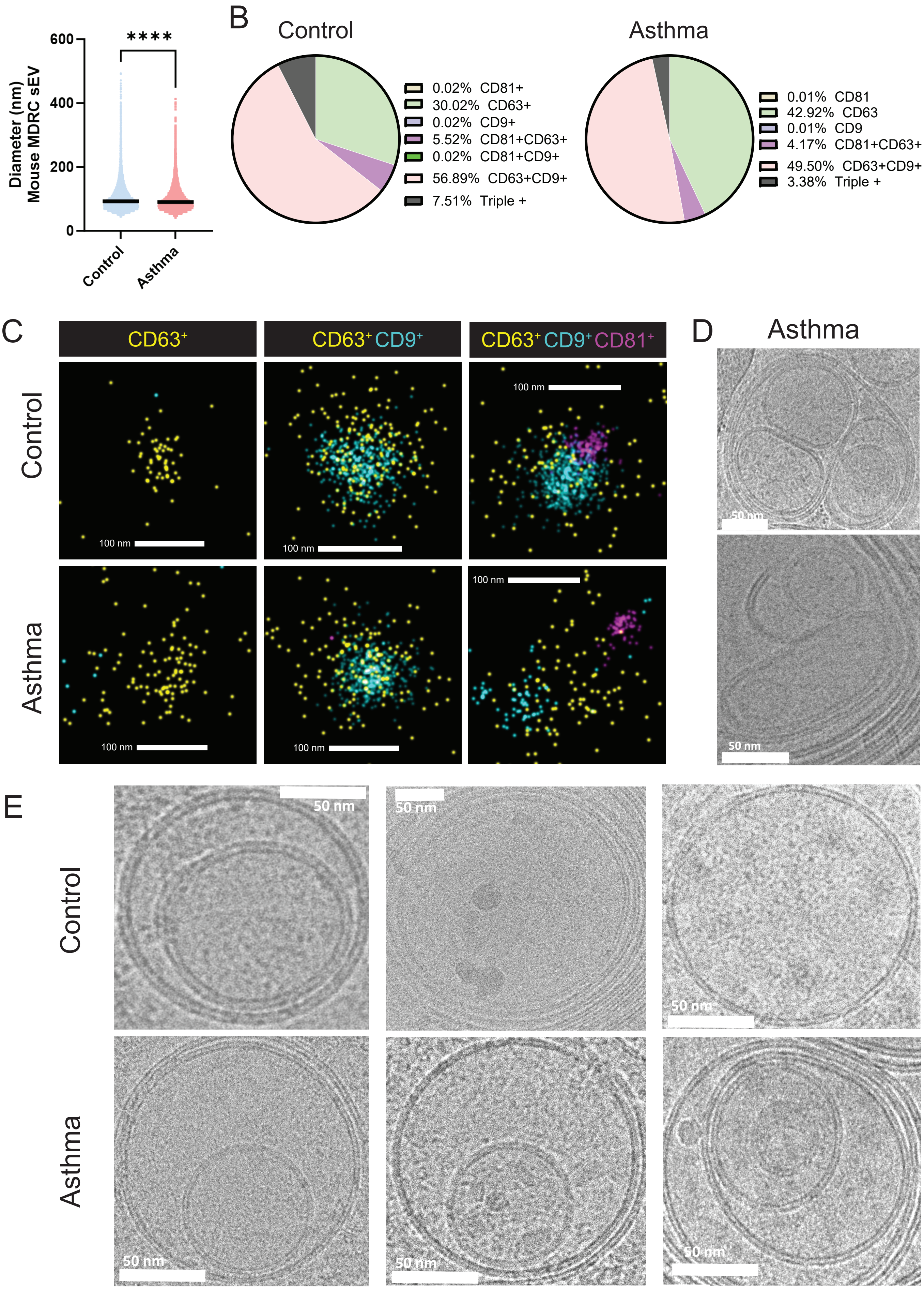

Diameter (nm) Mouse MDRC sEV
600
400
200
0
****
Control
Asthma
B
Control
Asthma
0.02% CD81+
30.02% CD63+
0.02% CD9+
5.52% CD81+CD63+
0.02% CD81+CD9+
56.89% CD63+CD9+
7.51% Triple +
0.01% CD81
42.92% CD63
0.01% CD9
4.17% CD81+CD63+
49.50% CD63+CD9+
3.38% Triple +
C
CD63+
CD63+ CD9+
CD63+ CD9+ CD81+
Control
Asthma
100 nm
D
Asthma
50 nm
E
Control
Asthma
50 nm

Supplementary Figure 13 – Murine MDRC EVs are small size EVs and they express tetraspanins. Purified MDRC EVs from MitoQC mice were captured and stained using the Oxford Nanoimaging (ONi) EV Profiler Kit 2. Imaging was completed using the AutoEV function on the ONi Nanoimager and utilized direct stochastic optical reconstruction microscopy for high resolution images. All analyses were completed in the CODI software. (A) Quantitation of diameter of pooled MDRC EVs isolated from MitoQC mice sensitized by OVA and challenged with PBS (Control) and from MitoQC mice sensitized and challenged with OVA (Asthma). n=18048 – 24809 sEVs per group (n=3 replicate samples), Kolmogorov Smirnov was utilized to compare groups, ****p<0.001. Individual data points presented with bar representing mean. (B) Pie charts showing % of CD9, CD81 and CD63 expressing MDRC sEVs from samples in (A). (C) Representative high-resolution images showing sEVs expressing CD63, CD9 or CD81 isolated from MDRCs from n=5 mice/group. Scale bar= 100 nm. (D) Representative Cryo-Electron microscopy images showing Multi-vesicular inclusions in MDRC sEVs from Asthma group, Scale bar= 50 nm. (E) Representative Cryo-Electron microscopy images showing lipid bilayered sEVs with electron dense inclusions in purified MDRC sEVs from Control and Asthma groups above, Scale bar= 50 nm. Source data are provided as a Source Data file.

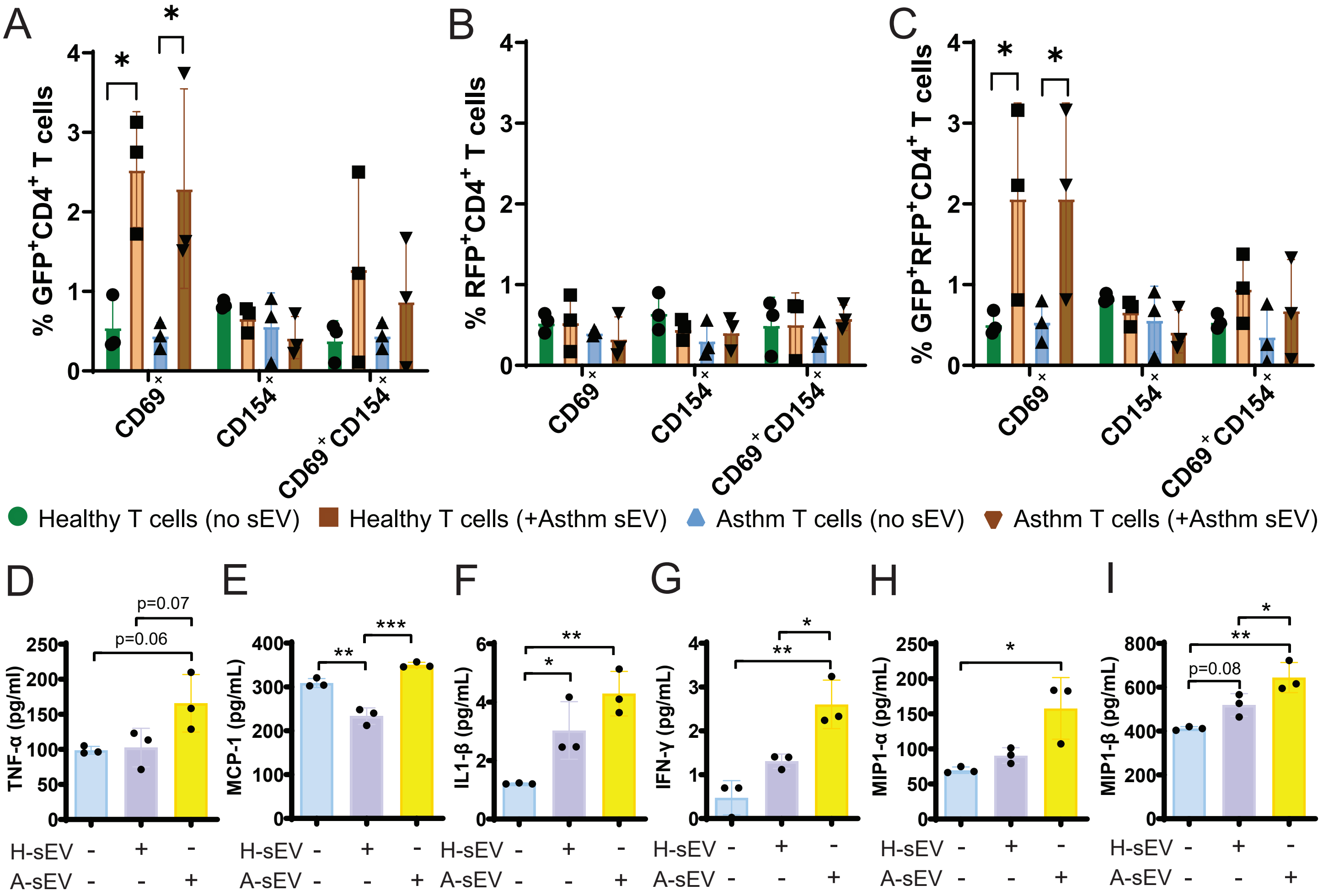

Supplementary Figure 14 – (A-C) MDRCs were transduced with CellLight Mito-GFP and CellLight Endo-RFP, and sEVs were purified from the supernatant 48 hours later. Purified MDRC sEVs were then co-cultured with their allogenic T cells (i.e. healthy sEV on asthmatic T cells, and vice versa; at a 1:10 T cell:sEV) for 24 hours. Activation was assessed by CD69 and CD154 expression using the ImageStream cytometry. n=3, Two way ANOVA; *p<0.05. (D-I) Airway sEVs were purified from BALF using a described ultracentrifugation method. Purified sEVs were cultured with THP-1 cells (1:10 cell:sEV) for 24 hours. Supernatants were harvested for BioRad Bioplex analysis of cytokines for (D) TNF-α, (E) MCP-1, (F) IL1- β, (G) IFNγ, (H) MIP1-α, and (I) MIP1-β. n=3. One-way ANOVA, *p<0.05, **p<0.01, ***p<0.001. Individual data points presented with each bar representing mean ± SD. Source data are provided as a Source Data file.

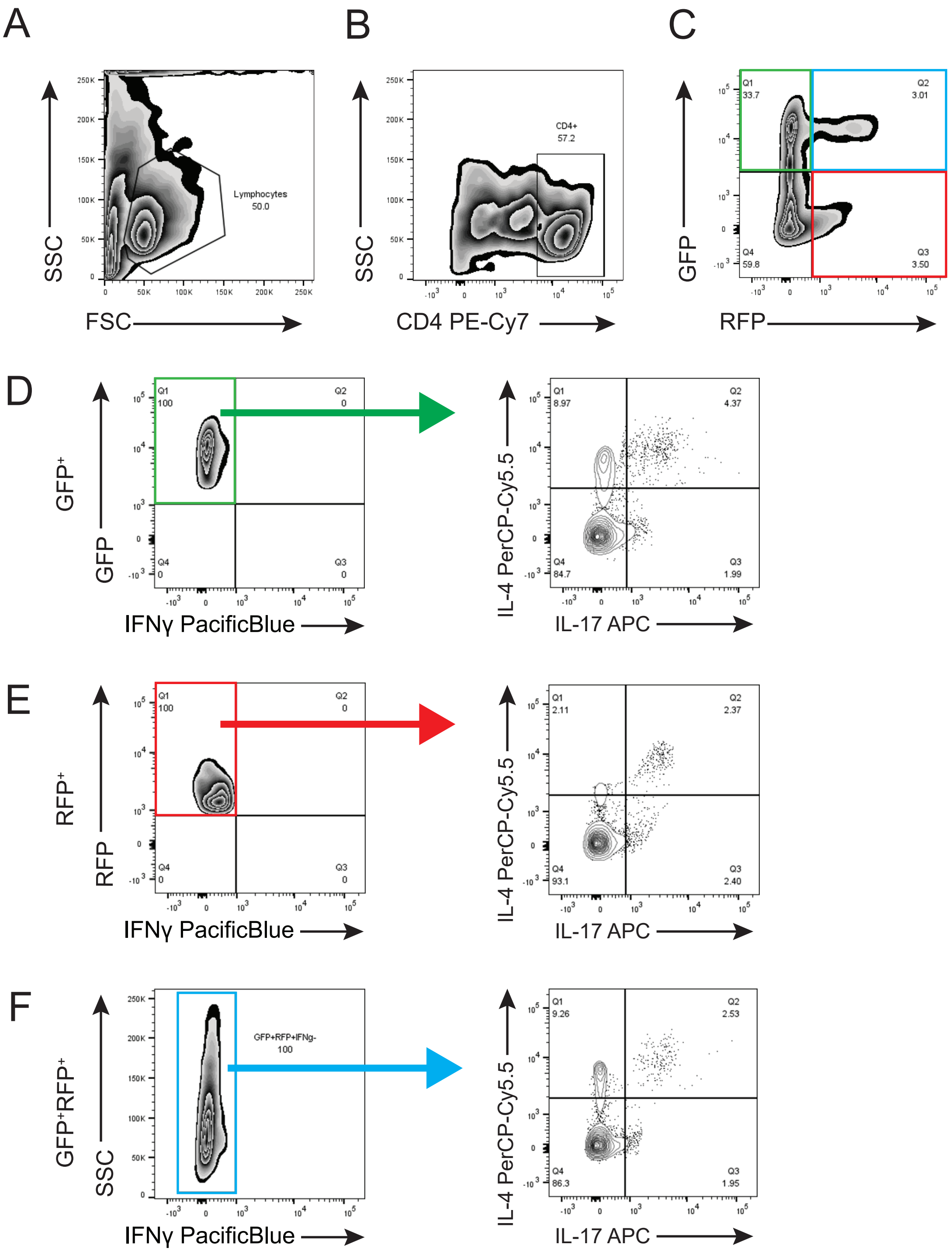

Supplementary Figure 15 – Gating Strategy for T cells from Healthy controls and Asthmatics that internalize MDRC-derived Mito-GFP^{+} sEVs and polarize to Th17 and Th2 subsets. Airway MDRCs were transduced with CellLight Mito-GFP and CellLight Endo-RFP, and sEVs were purified from the supernatant 48 hours later. Purified MDRC sEVs were co-cultured with autologous peripheral CD4^{+}T cells for 7 days in the presence of rhIL-2 (50 IU/ml) in a ratio of 1:10 T cells:sEVs. T helper subsets were assessed by flow cytometry 7 days later by staining intracellular for IL-4 (Th2) and IL-17(Th17) cytokines. (A-C) Cells were gated initially for CD4 followed by GFP^{+}, RFP^{+} or GFP^{+}RFP^{+} gate. (D) The IFNγ^{neg} were gated within the GFP^{+} gate and shown as IL-4^{+} cells or IL-4^{neg}IL-17^{+} cells, or IL-4^{+}IL-17^{+}. (E) The IFNγ^{neg} were gated within the RFP^{+} gate and shown as IL-4^{+} cells or IL-4^{neg}IL-17^{+} cells, or IL-4^{+}IL-17^{+}. (F) The IFNγ^{neg} were gated based on SSC within the GFP^{+}RFP^{+} gate and shown as IL-4^{+} cells or IL-4^{neg}IL-17^{+} cells or IL-4^{+}IL-17^{+}cells.

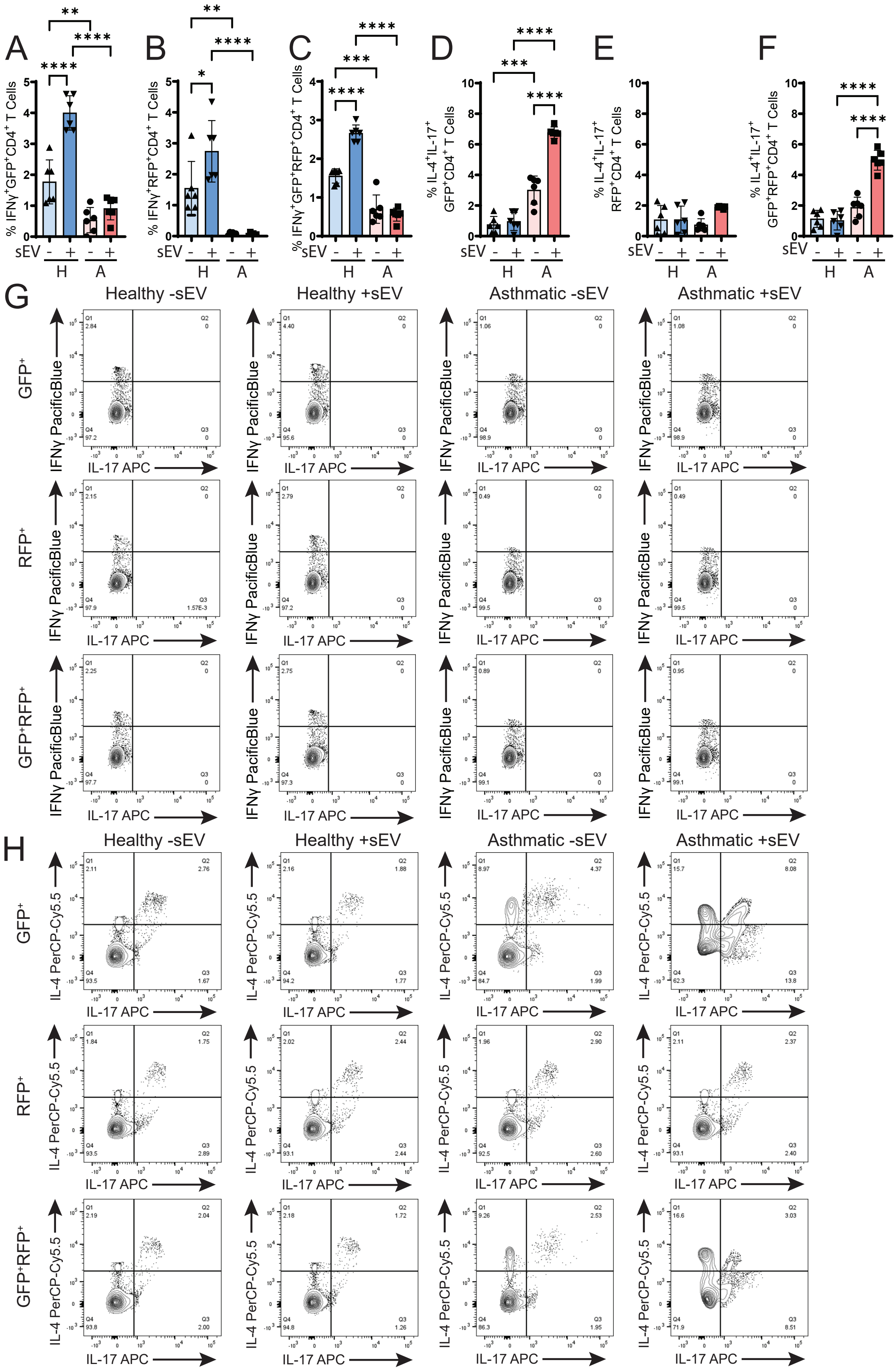

A
B
C
D
E
F
% IFNγ+GFP+CD4+ T Cells
% IFNγ+RFP+CD4+ T Cells
% IFNγ+GFP+RFP+CD4+ T Cells
% IL-4+IL-17+ GFP+CD4+ T Cells
% IL-4+IL-17+ RFP+CD4+ T Cells
% IL-4+IL-17+ GFP+RFP+CD4+ T Cells
sEV - + - +
H
A
G
Healthy -sEV
Healthy +sEV
Asthmatic -sEV
Asthmatic +sEV
GFP+
RFP+
GFP+RFP+
IFNγ PacificBlue
IL-17 APC
H
IL-4 PerCP-Cy5.5

Supplementary Figure 16 – Autologous peripheral $CD4^+$ T cells that internalize MDRC-derived Mito-GFP^+ sEVs purified from healthy subjects polarize to Th1 hybrid subset but not in asthmatics. Autologous peripheral $CD4^+$ T cells in asthmatics that internalize MDRC-derived Mito-GFP^+ sEVs polarize also to Th2/Th17 hybrid subset. Purified MDRC sEVs were co-cultured with autologous peripheral $CD4^+$ T cells for 7 days in the presence of rhIL-2 (50 IU/ml) in a ration of 1:10 T cells:sEVs. T helper subsets were assessed by flow cytometry 7 days later by staining intracellular for IFNγ (Th1), IL-4 (Th2) and IL-17 (Th17) cytokines. (A-C, & G) Cells were first gated on GFP^+, RFP^+, or GFP^+RFP^+, then IL-4^{neg} and GFP^+RFP^+, SSC was used for y-axis and gated on IL-4^{neg}IL-17^{neg} and the $IFN\gamma^+$ population plotted and quantitated. Mixed Effect ANOVA with Sidak's multiple comparisons test, n=6, *$p<0.05$, **$p<0.01$, ***$p<0.001$, ****$p<0.0001$. (D-F, & H) Co-culture was performed as in (A-C). T helper subsets were assessed by flow cytometry 7 days later by staining intracellular for IL-4 (Th2) and IL-17 (Th17) cytokines. Cells were first gated on GFP^+, RFP^+, or GFP^+RFP^+, then gated on $IFN\gamma^{neg}$ cells as in Figures 8 and S15, and then plotted IL-4 versus IL-17 and determined % IL-4^+IL-17^+ cells. n=6 as above. Individual data points presented with each bar representing mean ± SD. Source data are provided as a Source Data file.

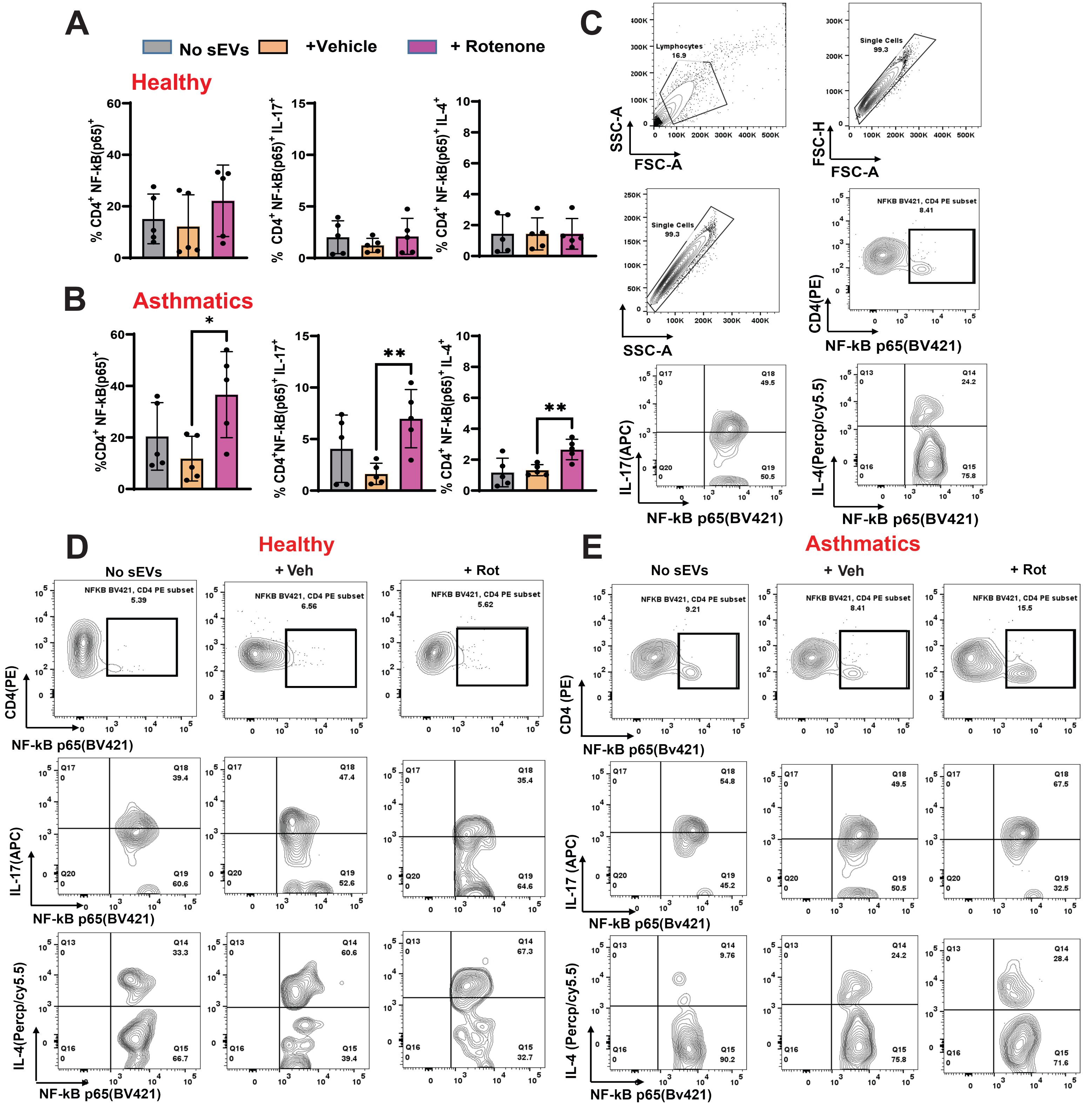

Supplementary Figure 17 – Human CD4+ T cells co-cultured with human BALF sEVs treated with rotenone, a Complex I inhibitor, showed enhanced percent of CD4+ T cells with activation of NF- B p65 and increased Th2 and Th17 polarization in the co-cultures. Purified human BALF sEVs from healthy and asthmatic subjects were treated with 10 μM rotenone or vehicle control, washed and purified. Autologous CD4+ T cells were co-cultured with these sEVs at a 10 sEV: 1 T cell ratio or with no sEVs and 7 days later assessed for intracellular expression of IL-17 and IL-4 within the NF-kB p65(pS529)+ CD4+ T cells. Quantitation of flow cytometry analyses of NF-kB p65 (pS529)+ CD4+ T cells and percent of Th2 and Th17 cells within this population from n=5 samples from (A) Healthy study subjects (B) Asthmatic study subjects. n=5, One way ANOVA analyses with multiple comparison between treatments and study groups, $^{**}p<0.01$, $^{*}p<0.05$. (C) Gating strategy for SSC-FSC and singlets and the cell populations described above. Representative flow cytometry plots showing gating of NF-kB p65 (pS529)+ CD4+ T cells and IL-17+ and IL-4+ cells within the gated cells in (D) samples from Healthy study subjects (E) samples from Asthmatic study subjects. Individual data points presented with each bar representing mean ± SD. Source data are provided as a Source Data file.

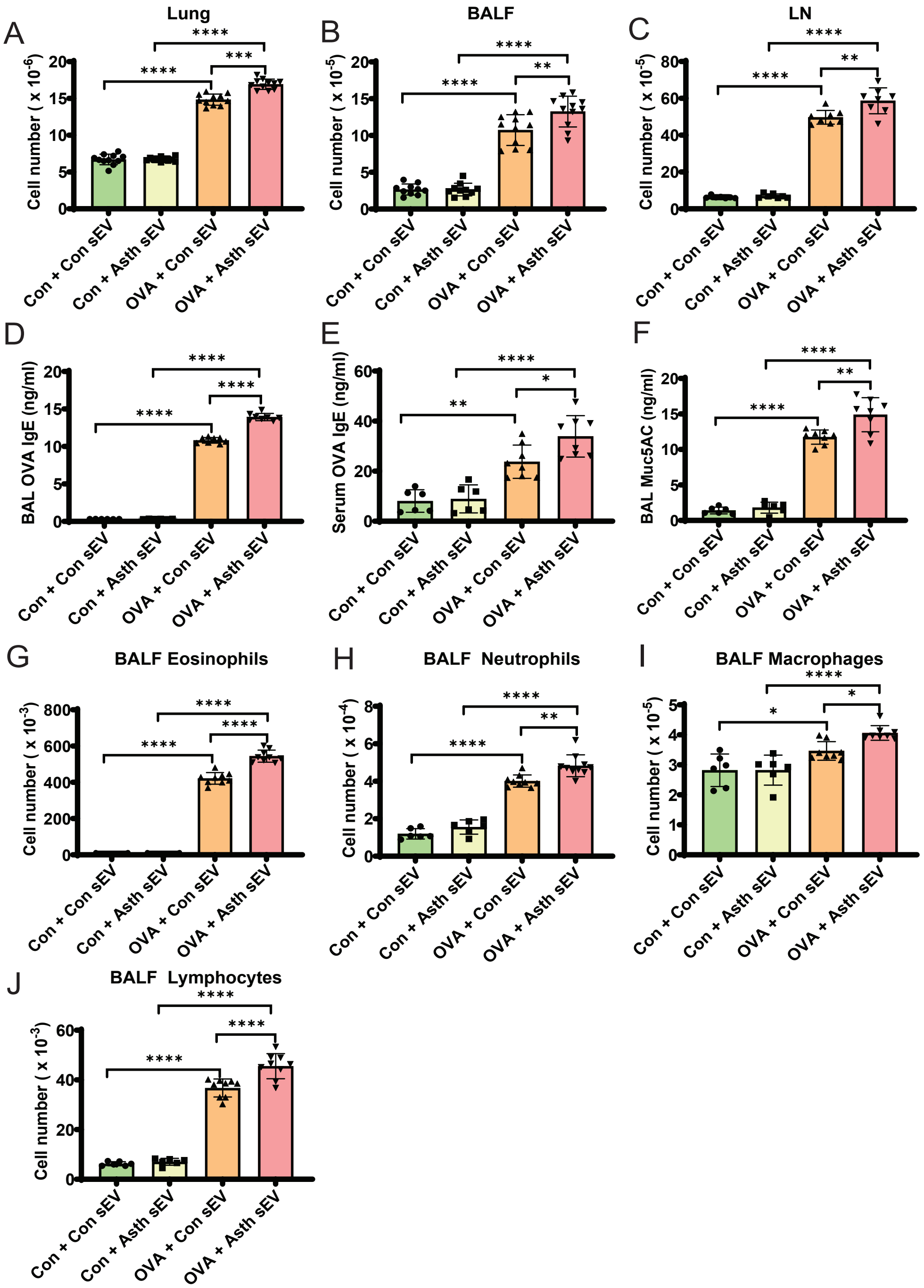

Supplemental Figure 18 – Intranasal (i.n.) transfer of asthmatic lung MDRC-derived sEVs exacerbates airway inflammation. Mice were sensitized by intraperitoneal injection on d0 and d7 with 50 µg of alum-adsorbed OVA. On d14, d15 & d16 mice were challenged once i.n. with 15 µg OVA in 30 µl PBS or PBS alone. On d16, 4 hrs after i.n. challenge with OVA or PBS, i.n. delivery of lung proinflammatory MDRC-derived sEVs (1 x 10^8 particles/-mouse in 30 µl PBS) from control or OVA challenged donor Mito-QC mice was carried out. (A). Total lung cells from sEV recipient controls and OVA challenged mice at two days after transfer of sEVs. Donor Mito-QC mice were used at n=5 mice per group for isolation of MDRC-sEVs. (B). Total BALF cells from sEV-recipient controls and OVA challenged mice at two days after sEV transfer. (C) Total cells of lung draining LN from sEV-recipient controls and OVA challenged mice at two days after sEV transfer. (D). OVA-IgE levels in BALF harvested at two days after sEV delivery detected by ELISA. (E). OVA-IgE levels in sera at two days after sEV delivery detected by ELISA. (F). Muc5AC levels in BALF by ELISA at two days after-sEV delivery. BALF cells were collected and stained by Diff-Quik. Differential analysis was performed using standard morphological criteria on cytospin slides. 300 cells were examined in each cytospin slide. Numbers of eosinophils (G), neutrophils (H), macrophages (I), and lymphocytes (J) were calculated based on the percentage of each cell population. n=6-11 recipient mice/group,. One-way ANOVA with Tukey's multiple comparison testing. * $p<0.05$, ** $p<0.01$, *** $p<0.005$, **** $p< 0.001$. Individual data points presented with each bar representing mean ± SD. Source data are provided as a Source Data file.

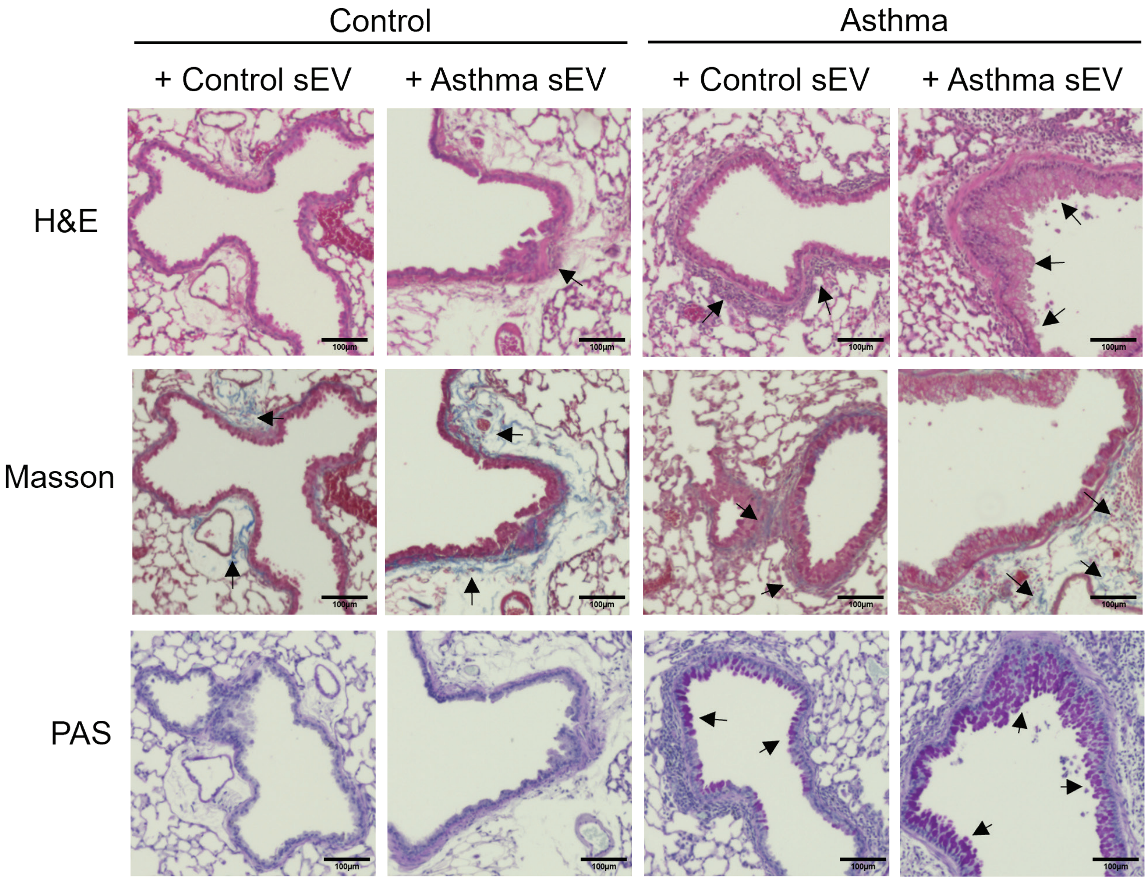

Supplementary Figure 19 – Intranasal transfer of pro-inflammatory lung MDRC-derived sEVs from sensitized and challenged donor Mito-QC mice with asthma enhanced inflammatory cell infiltration, goblet cell hyperplasia and mucus hyper secretion in sensitized and challenged recipients. Magnified photomicrographs of lung sections (shown in Figure 9) from recipient experimental groups stained with hematoxylin and eosin for morphological analysis and assessment of inflammatory cell infiltration (top panel), Masson Trichrome to compare collagen accumulation (middle panel), and periodic acid-schiff (PAS) to assess changes in goblet cell hyperplasia and mucus production (bottom panel).

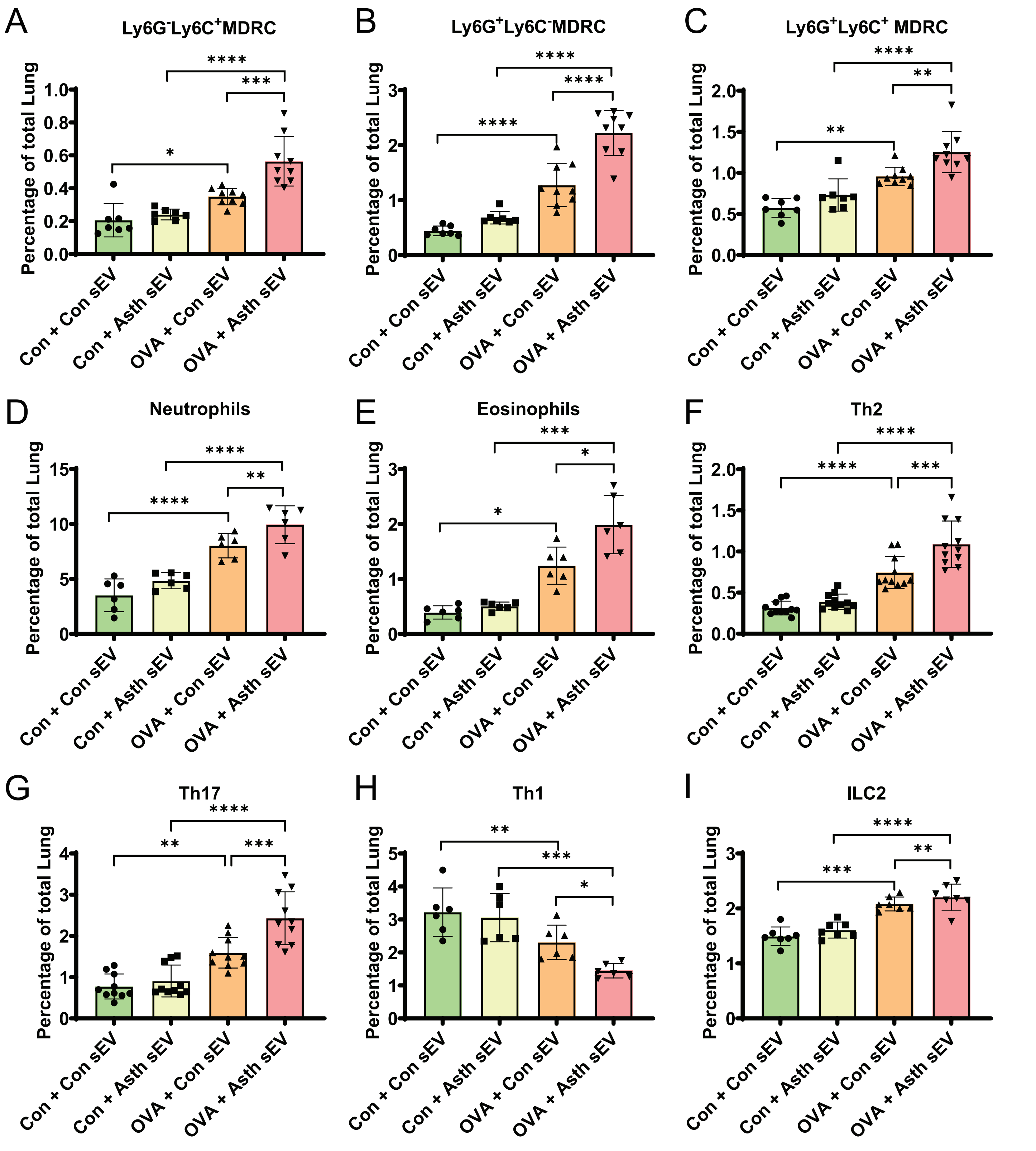

Supplemental Figure 20 - Intranasal transfer of pro-inflammatory lung MDRC-derived sEVs from sensitized and challenged donor Mito-QC mice with asthma exacerbates Th2 and Th17 responses and allergic airway inflammation in sensitized and challenged recipients. Mice were sensitized and challenged as in Supplemental Figure 19. On d16, i.n. delivery of lung MDRC-derived sEVs (1 x 10^8 particles/mouse in 30 µl PBS) from control or OVA challenged Mito-QC mice were carried out as before. MDRC-sEVs were isolated from n=5 mice per group donor Mito-QC mice. Lung infiltration of immune cells in lung tissue was determined by FACS analyses. Cell numbers of $Ly6G^{neg}Ly6C^{+}$ MDRCs (A), $Ly6G^{+}Ly6C^{neg}$ MDRCs (B), $Ly6G^{+}Ly6C^{+}$ MDRCs (C), Neutrophils (D), Eosinophils (E), Th2 cells (F), Th17 cells (G), Th1 cells (H), and ILC2 cells (I) in the lung tissue were determined by flow cytometry. n=6-11 recipient mice/group. One-way ANOVA with Tukey's multiple comparison testing. *p<0.05, **p<0.01, ***p<0.005, ****p<0.001. Individual data points presented with each bar representing mean ± SD. Source data are provided as a Source Data file.

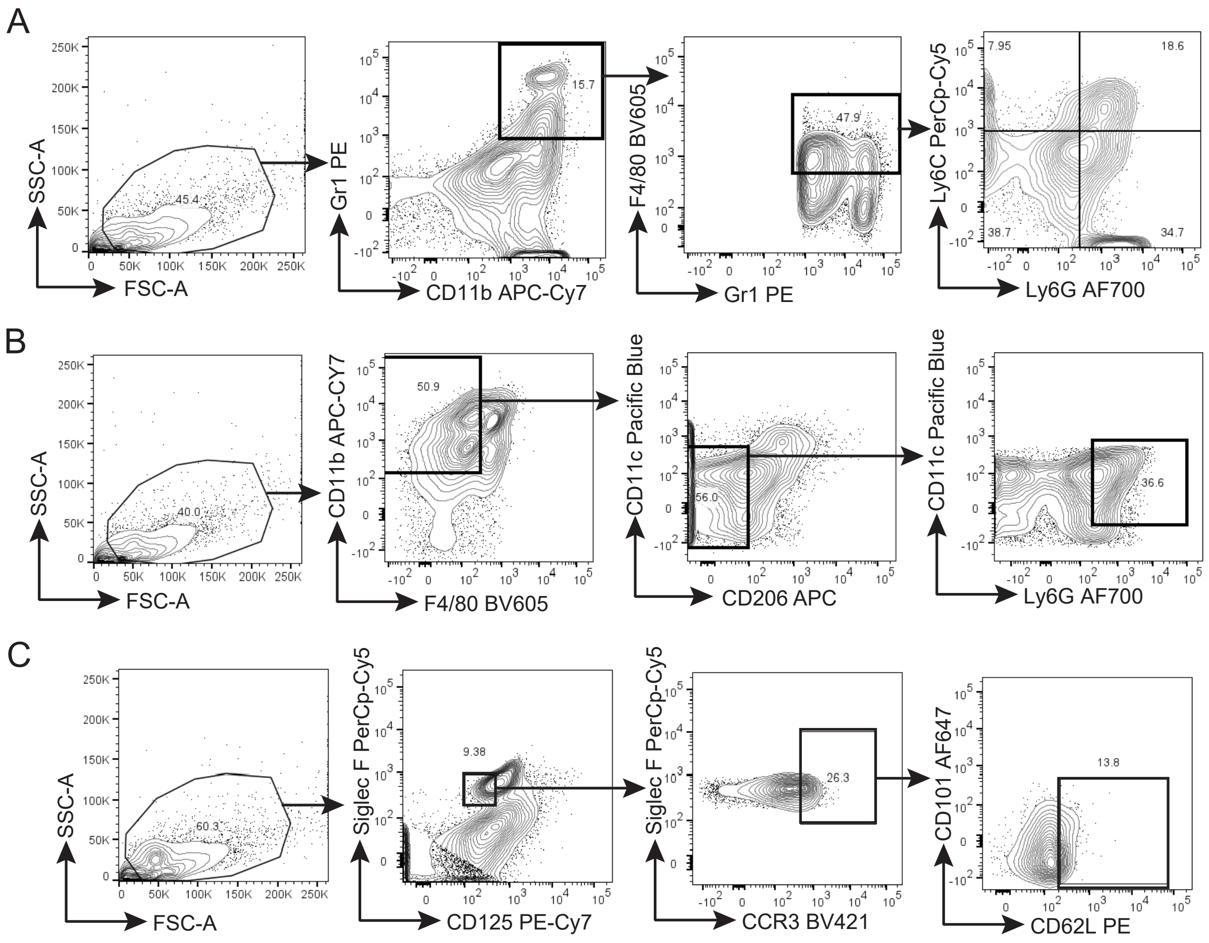

Supplementary Figure 21 – Gating strategies for myeloid cell populations in the lung tissues of sensitized and challenged mice that are recipients of MDRC-derived sEVs from control or OVA challenged-Mito-QC mice. Mice were sensitized by intraperitoneal injection on d0 and d7 with 50 µg of alum adsorbed OVA. On d14, d15 & d16 mice were challenged once i.n. with 15 µg OVA in 30 µl PBS or PBS alone. On d16, i.n. delivery of lung MDRC-derived sEVs (1 x 108 particles/mouse in 30 µl PBS) from control or OVA challenged Mito-QC mice were carried out as before. Lung infiltration of immune cells in lung tissue was determined by FACS analyses. (A) Gating strategy for MDRC-subpopulations in the lung tissue. (B) Gating strategy for neutrophils in the lung tissue. (C) Gating strategy for eosinophils in the lung tissue.

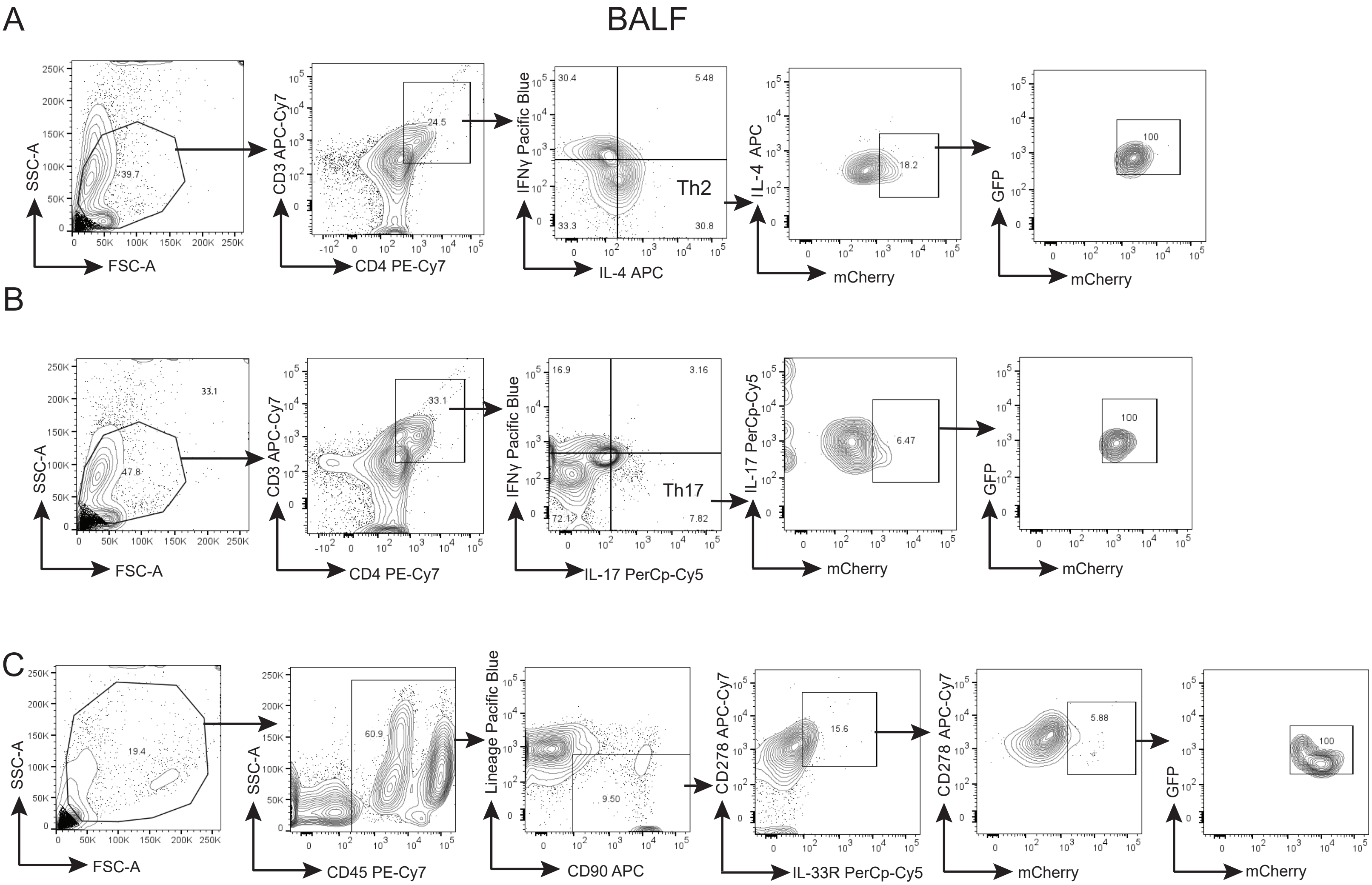

Supplementary Figure 22 - Gating strategies for mCherry$^+$GFP$^+$ Th2 and Th17 cells in the BALF of sensitized and challenged recipients. Mice were sensitized by intraperitoneal injection on d0 and d7 with 50 µg of alum-adsorbed OVA. On d14, d15 & d16 mice were challenged once i.n. with 15 µg OVA in 30 µl PBS or PBS alone. On d16, i.n. delivery of lung MDRC-derived sEVs (1 x 108 particles/mouse in 30 µl PBS) from control or OVA challenged Mito-QC mice were carried out as before. Infiltration of immune cells in BALF was determined by FACS analyses. (A) Gating strategy for Th2 cells showing mCherry$^+$ and mCherry$^+$GFP$^+$ cells in the BALF. (B) Gating strategy for Th17 cells showing mCherry$^+$ and mCherry$^+$GFP$^+$ cells in BALF. (C) Gating strategy for ILC2 cells showing mCherry$^+$GFP$^+$ cells in the BALF.

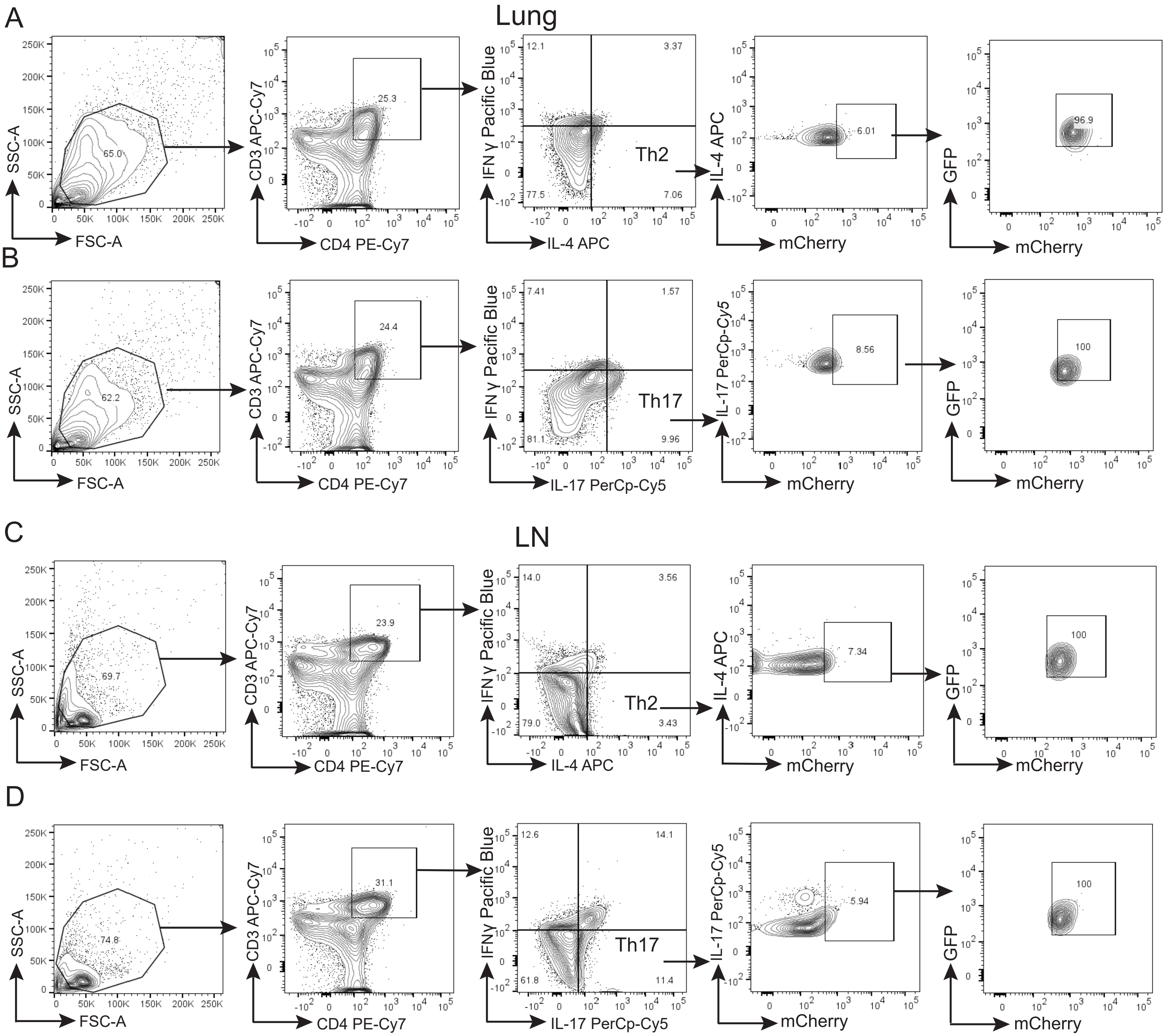

Supplementary Figure 23 - Gating strategies for mCherry^{+}GFP^{+} Th2 and Th17 cells in the lung tissue and draining LN of sensitized and challenged recipients. Mice were sensitized by intraperitoneal injection on d0 and d7 with 50 µg of alum-adsorbed OVA. On d14, d15 & d16 mice were challenged once i.n. with 15 µg OVA in 30 µl PBS or PBS alone. On d16, i.n. delivery of lung MDRC-derived sEVs (1 x 108 particles/mouse in 30 µl PBS) from control or OVA challenged Mito-QC mice were carried out as before. Lung infiltration of immune cells in lung tissue was determined by FACS analyses. (A) Gating strategy for Th2 cells showing mCherry^{+} and mCherry^{+}GFP^{+} cells in the lung. (B) Gating strategy for Th17 cells showing mCherry^{+} and mCherry^{+}GFP^{+} cells in the lung. Immune cell populations in draining LN tissue were detected by FACS analyses. (C) Gating strategy for Th2 cells showing mCherry^{+}GFP^{+} population in the LN. (D) Gating strategy for Th17 cells showing mCherry^{+} and mCherry^{+}GFP^{+} population in the LN.

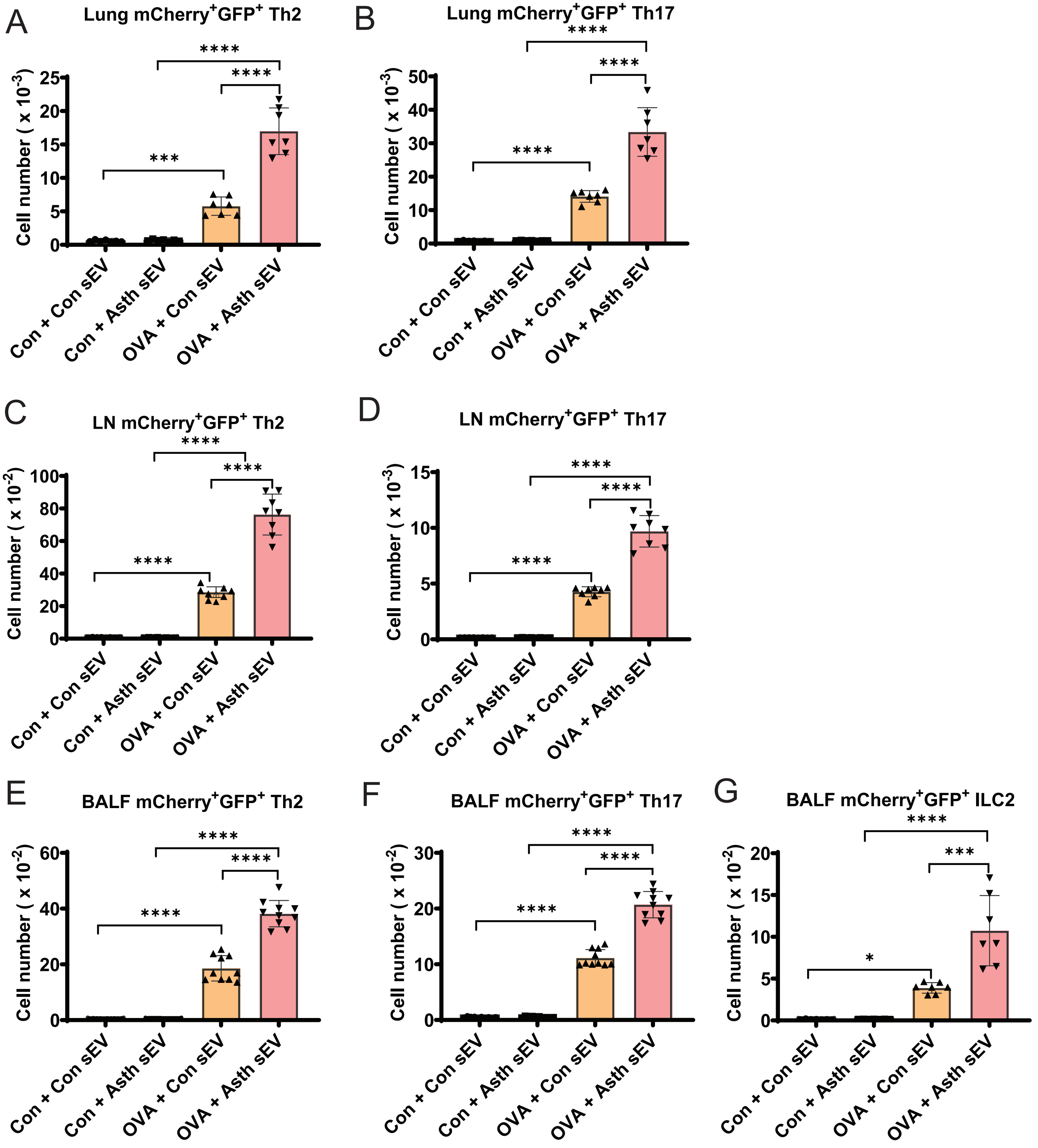

Supplemental Figure 24 - Intranasal transfer of pro-inflammatory lung MDRC-derived sEVs from sensitized and challenged donor Mito-QC mice with asthma enhances mCherry$^+$GFP$^+$ Th2 and Th17 cell infiltrations in the lung tissue, draining LNs, and BALF in sensitized and challenged recipients. Mice were sensitized by intraperitoneal injection on d0 and d7 with 50 µg of alum-adsorbed OVA. On d14, d15 & d16 mice were challenged once i.n. with 15 µg OVA in 30 µl PBS or PBS alone. On d16, i.n. delivery of lung MDRC-derived sEVs (1×10^8 particles/mouse in 30 µl PBS) from control or OVA challenged Mito-QC mice were carried out as before. MDRC-sEV isolation was from n=5 mice/group. Infiltration of immune cells in the lung, BALF and draining LNs was determined by FACS analyses. Cell numbers of mCherry$^+$GFP$^+$ Th2 (A) and Th17 (B) in the lung tissue were determined by flow cytometry. Cell numbers of mCherry$^+$GFP$^+$ Th2 (C) and Th17 (D) in draining LNs were determined by flow cytometry. Cell numbers of mCherry$^+$GFP$^+$ Th2 (E), Th17 (F) and ILC2 (G) in BALF were determined by flow cytometry. n=7-10 mice/group. One-way ANOVA with Tukey's multiple comparison testing. *$p<0.05$, **$p<0.01$, ***$p<0.005$, ****$p<0.001$. Individual data points presented with each bar representing mean ± SD. Source data are provided as a Source Data file.

Supplementary Table 1

Reagent or Resource	Source	Identifier
**Chemicals & Reagents & Kits**		
Ficoll Paque	GE Healthcare	Cat: 45-001-749
Human CD4^{+} T cells Enrichment Cocktail	STEMCELL Technologies	Cat: 15062
HLA Myeloid Cell Enrichment Cocktail	STEMCELL Technologies	Cat: 15272H
Ketamine hydrochloride	Zoetis	Cat: 000680
Xylazine	Dechra	Cat: Romp100-50
Isoflurane	Vet One	Cat: 03060
Ova-IgE ELISA Assay Kit	Biolegend	Cat: 439807
MUC5AC ELISA Assay Kit	MyBioSource	Cat: MBS265057
RPMI	Corning	Cat: 15-040-CV
FBS	Atlas Biologicals	Cat: F-0500-DR
Penicillin-Streptomycin	MP Biomedicals, Inc	Cat: ICN1674049
Glutamine	Corning	Cat: MT25005CI
Human AB Heat Inactivated Serum	Corning	Cat: 35-060-CI
Recombinant Human IL2	R&D Systems	Cat: 202-IL-010
0.2 μm Cellulose Acetate Filter	Corning	Cat; 430320
Total Exosome Isolation Kit	Thermo Fisher Scientific	Cat: 4478362
100 nm Polystyrene Latex Microspheres	Malvern	Cat: NTA4088
300 Mesh Copper Lacey Carbon Grids	Ted Pella	Cat: 01895F
Pelco easiGlow Glow Discharger	Ted Pella	Cat: 91000
EV Profiler Kit 2	ONi	Cat: 900-00206
10X RIPA Buffer	Cell Signaling	Cat: 9806S
25X Protease Inhibitor Cocktail	Thermo Fisher Scientific	Cat: A32961
0.45 Immobilon PVDF Membrane	Millipore	Cat: IPVH00010
BSA	Thermo Fisher Scientific	Cat: BP1600-100

Immobilon Western Chemiluminescent HRP Substrate	Millipore	Cat: WBKLS0500
PKH26	Sigma	Cat: MINI26-1KT
CellTrace CFSE	Invitrogen	Cat: 50-591-407
Apogee Bead Mix	Apogee Flow Systems Ltd.	Cat: 1493
BD CytoPerm/CytoFix Kit	BD Biosciences	Cat:554655
Rotenone	Sigma	Cat: R-8875
MitoTracker Green	Thermo Fisher Scientific	Cat: M7514
Permeabilization Buffer	eBioscience	Cat: 00-5523-00
MitoView 633	Biotium	Cat: 70055
Thenoyltrifluoroacetone (TTFA)	Sigma	Cat: T-9888
Antimycin A	Sigma	Cat: A-8674
Oligomycin	Sigma	Cat: O-4876
MitoTEMPOL	Dr. Balaraman Kalyanaraman, University of Wisconsin	N/A
PitStop2 (50 nM)	Abcam	Cat: ab120687
Dynasore (50 nM)	Caymen Chemical	Cat: 14062
Quantikine Human IL-17 Immunoassay Kit	R&D Systems	Cat: D1700
Quantikine Human IL-4 Immunoassay Kit	R&D Systems	Cat: D4050
Quantikine Mouse IL-17 Immunoassay Kit	R&D Systems	Cat: M1700
Quantikine Mouse IL-4 Immunoassay Kit	R&D Systems	Cat: M4000B
PureLink RNA Mini Kit	Thermo Fisher Scientific	Cat: 12183018A
NucleoSpin@RNAplus	Takara	Cat: 740985.05
Trizol	Thermo Fisher Scientific	Cat: 15596018
Chloroform	Sigma	Cat: 472476-500ML
RNAse Free Molecular Grade Water	Fisher Scientific	Cat: BP561-1
PrimeScript 1st Strand cDNA Synthesis kit	Takara Bio	Cat: 6100A
cDNA Synthesis Kit	Takara Bio	Cat: RR039A

VeriQuest SYBR Green qPCR Master Mix	Thermo Fisher Scientific	Cat: 75600
TB Green Premix Ex Taq II	Takara	Cat: RR820A
Lipofectamine 3000	Thermo Fisher Scientific	Cat: L3000001
Micro BCA Protein Assay Kit	Thermo Fisher Scientific	Cat: 23225
P110 (1 μM)	Tocris	Cat: 6897
Mini-PROTEAN TGX StainFree 4-15%	BioRad	Cat: 456-8086
n-Dodecyl β-D-maltoside (DDM)	Invitrogen	Cat: BN2005
Native Page 4x Sample Buffer	Invitrogen	Cat: 3010934
Glycine	Fisher Scientific	Cat: BP381-1
Tris Base	Fisher Scientific	Cat: BP152-1
Sodium Chloride	Fisher Scientific	Cat: BP358-1
Tween 20	Fisher Scientific	Cat: BP337-500
Non-fat Dry Milk	Lab Scientific	Cat: M0841
10% Neutral Buffered Formalin	Fisher Scientific	Cat: 22050105
H&E Staining Kit	Vector Labs	Cat: H-3502
Masson Trichrome Staining Kit	Poly Scientific RnD Corp	Cat: k-037
Periodic Acid Schiff Staining Kit	Abcam	Cat: ab150680
Cytoseal XYL	Epradia	Cat: 8312-4
Diff-Quik	Millipore	Cat:
PBS	Corning	Cat: 21-031-CV
OVA (grade VII)	Sigma	Cat:
Collagenase B	Sigma	Cat: 11088831001
ACK Lysis Buffer	Fisher Scientific	Cat: 50-983-219
Miliplex MAP Human cytokine/chemokine	EMD Millipore	Cat: HCYTOMAG-60K
**Viral Vectors**		
CellLight Mitochondria-GFP, BacMam 2.0	Thermo Fisher Scientific	Cat: C10508

CellLight Early Endosome-RFP, BacMam 2.0	Thermo Fisher Scientific	Cat: C10587
CellLight Lysosomes-RFP, BacMam 2.0	Thermo Fisher Scientific	Cat: C10504
CellLight Tubulin-RFP, BacMam 2.0	Thermo Fisher Scientific	Cat: C10503
**Oligonucleotides**		
Human ACTB (forward primer)<br>5`-TGCTATCCAGGCTGTGCTAT-3`	Hecker, et al., 2014	N/A
Human ACTB (reverse primer)<br>5`-AGTCCATCACGATGCCAGT-3`	Bernard, et a., 2015	N/A
GAPDH (forward primer)<br>5’ GTCTCCTCTGACTTCAACAGCG 3’	Eurofins Genomics	(Gene Accession#NM_002046)
GAPDH (reverse primer)<br>5’ACCACCCTGTTGCTGTAGCCAA 3’	Eurofins Genomics	(Gene Accession#NM_002046)
Human IL17 A (forward primer)<br>5`-TACTACAACCGATCCACCTC-3`	Cytogence Genie<br>https://genie.cytogence.com	Primer ID: 301342
Human IL17A (reverse primer)<br>5`-GAGTTCATGTGGTAGTCCAC-3`	Cytogence Genie<br>https://genie.cytogence.com	Primer ID: 301342
Human IL4 (forward primer)<br>5`-ACGGACACAAGTGCGATATC-3`	Cytogence Genie<br>https://genie.cytogence.com	Primer ID: 302718
Human IL4 (reverse primer)<br>5`-CTTCTCATGGTGGCTGTAGA-3`	Cytogence Genie<br>https://genie.cytogence.com	Primer ID: 302718
RELA (forward primer)<br>5’ TGAACCGAAACTCTGGCAGCTG 3’	Eurofins Genomics	(Gene Accession#NM_021975)
RELA (reverse primer)<br>5’ CATCAGCTTGCGAAAAGGAGCC 3’	Eurofins Genomics	(Gene Accession#NM_021975)
GATA-3 (reverse primer)<br>5’-TCGGTTTCTGGTCTGGATGCCT-3’	Eurofins Genomics	(Gene Accession#NM_001002295)

GATA-3 (forward primer) 5’ACCACAACCACACTCTGGAGGA-3’	Eurofins Genomics	(Gene Accession#NM_0010 02295)
RORC (forward primer) 5’GAGGAAGTGACTGGCTACCAGA 3’	Eurofins Genomics	(Gene Accession#NM_0050 12)
RORC (reverse primer) 5’GCACAATCTGGTCATTCTGGCAG 3’	Eurofins Genomics	(Gene Accession#NM_0050 12)
Human DNM1L (forward primer) 5`-GCTCCAGGACGTCTTCAACA-3`	This Paper (Designed using UCSC Genome Browser and in silico PCR for hg38)	N/A
Human DNM1L (reverse primer) 5`-TAGCACTGAGCTCTTTCCGC-3`	This Paper (Designed using UCSC Genome Browser and in silico PCR for hg38)	N/A
ON-TARGETplus SMARTpool Human DNM1L	Dharmacon	Cat: L-012092-00-0005
**Antibodies**		
CD11b APC Cy7 (clone: ICRF44; 1:200)	BD Biosciences	Cat: 17-0118-42
CD169 BV510 (clone: 7-239; 1:200)	BD Biosciences	Cat: 742992
HLA-DR APC (clone: LN3; 1:200)	eBioscience	Cat: 17-9956-42
CD163 PE (clone: eBioGHI/61; 1:200)	eBioscience	Cat: A15792
CD33 PE-Cy7 (clone: WM53; 1:200)	eBioscience	Cat: 25-0338-42
CD14 PerCp-Cy5.5 (clone: 61D3; 1:200)	eBioscience	Cat: 45-0149-42
CD11c PE-Cy5 (clone: 3.9; 1:200)	eBioscience	Cat: 15-0116-42
Tomm20 (1:200)	Abcam	Cat: 56783
Donkey anti-mouse AZ647	ONi	Cat: 800-00089
Tim23 (clone: polyclonal; 1:1000)	Proteintech	Cat: 11123-1-AP
CD81 (clone: D3N2D; 1:500)	Cell Signaling	Cat: 56039
Anti-rabbit HRP (1:5000)	Promega	Cat: PR-W4011
CD63 eFlour450 (clone: H5C6; 1:200)	Affymetrix, Inc.	Cat: 48-0639-42
HLA-DR APC (clone: LN3; 1:200)	Affymetrix, Inc.	Cat: 17-9956-42
CD54 PE (clone: 86 HA58; 1:200)	Affymetrix, Inc.	Cat: 12-0549-42
CD9 PE (clone: M-L13; 1:200)	BD Biosciences	Cat: 341637
CD81 PE-Cy7 (clone: 5A6; 1:200)	BioLegend	Cat: 349511
TSG101 Alexa Fluor 647 (clone: 4A10; 1:200)	Novus Biologicals	Cat: NB200-112AF647
CD4 PE-Cy7 (clone: SK3; 1:200)	Thermo Fisher Scientific	Cat: 25-0041-81

IL-4 PE (clone: 8D4-8; 1:200)	Thermo Fisher Scientific	Cat: 12-7049-42
IL-17A APC (clone: eBio64DEC17; 1:200)	Thermo Fisher Scientific	Cat: 17-7179-42
IFNγ BV421 (clone: B27; 1:200)	BD Biosciences	Cat: 562988
CD69 eFluor450 (clone: FN50; 1:200)	Thermo Fisher Scientific	Cat: 48-0699-41
CD154 APC (clone: 24-31; 1:200)	Thermo Fisher Scientific	Cat: 17-1548-41
pZap70 Alexa Fluor 647 (clone: 17A/P-ZAP70; 1:200)	BD Bioscience/BD Phosflow	Cat: 557817
α-tubulin eFluor 615 (clone: DM1A; 1:200)	Thermo Fisher Scientific	Cat: 42-4502-82
NF-κB p65 BV421 (clone: K10-895.12.50; 1:200)	BD Biosciences	Cat: 565446
CD4 PE (clone: RPA-T4, 1:200)	Invitrogen	Cat: 12-0049-42
IL-17A APC (clone: eBio64Dec17, 1:200)	Thermo Fisher Scientific	Cat: 17-7179-42
IL-4 PerCP-Cy5.5 (clone: 8D4-8, 1:200)	BD Biosciences	Cat: 561234
LFA-1 (clone: R7.1; 1 μg/mL)	Fisher Scientific	Cat: 50-176-82
Pan-HLA (DR/DP/DQ; clone: TU39; 10 μg/mL)	BD Biosciences	Cat: 555557
Drp1 (Clone: 8; 1:1000)	BD Bioscience	Cat: 611113
MHC-II eFlour450 (clone: M5/114/15.2; 1:200)	Life Technologies	Cat: 48-5321-82
2.4G2 (anti-mouse CD16/CD32, FC block)	BD Biosciences	Cat: 553142
CD63 APC (clone: NVG2; 1:200)	Life Technologies	Cat: 17-0631-82
CD81 PE (clone: Eat-2; 1:200)	Biolegend	Cat: 104905
CD9 PE-Cy7 (clone: MZ3; 1:200)	Biolegend	Cat: 124816
CD45 PE (clone: 30-F11; 1:200)	Life Technologies	Cat: 12-0451-82
CD4 PE-Cy7 (clone: GK1.5; 1:200)	Life Technologies	Cat: 25-0047-41
CD69 eFluor450 (clone: H1.2F3; 1:200)	Life Technologies	Cat:48-0691-82
Gr-1 PE (clone: RB6-8C5; 1:200)	Life Technologies	Cat: 12-5931-82
CD25 PE (clone: PC61.5; 1:200)	Life Technologies	Cat: 12-0251-82
CD62L PE (clone: MEL-14; 1:200)	Life Technologies	Cat: 12-0621-82
CD206 APC (clone: MR6F3; 1:200)	Life Technologies	Cat: 17-2061-82
IL-4 APC (clone: 11B11; 1:200)	Life Technologies	Cat: 17-7041-81
Ly6C PerCp-Cy5.5 (clone: HK1.4; 1:200)	Life Technologies	Cat: 45-5932-82
CD170 PerCp-eFluor 710 (Siglec F; clone: 1RNM44N; 1:200)	Life Technologies	Cat: 46-1702-80
MHC-II PE-Cy5 (I-A/I-E; clone: M5/114.15.2; 1:200)	Life Technologies	Cat: 15-5321-82
CD4 PE-Cy7 (clone: GK1.5; 1:200)	Life Technologies	Cat: 25-0041-81
F4/80 BV605 (clone: T45-2342; 1:200)	BD Biosciences	Cat: 743281
CD11b APC-Cy7 (clone: M1/70; 1:200)	BD Biosciences	Cat: 557657
CD3 APC-Cy7 (clone: 145-2C11; 1:200)	BD Biosciences	Cat: 557596
Ly6G AlexaFluor 700 (clone: 1A8; 1:200)	BD Biosciences	Cat: 561236
CD101 AlexaFluor 647(Igsf2; clone: 307707; 1:200)	BD Biosciences	Cat: 564473

CD278 PE (ICOS; clone: C398.4A; 1:200)	Biolegend	Cat: 313507
IL-17A PE (clone: TC11-18H10.1; 1:200)	Biolegend	Cat: 506903
IL-33Rα PerCp-Cy5.5 (IL1RL1, ST2; clone: DIH9; 1:200)	Biolegend	Cat: 145311
CD45 PE-Cy7 (clone: 30-F11; 1:200)	Biolegend	Cat: 103113
CD4 PE-Cy7 (clone: GK1.5; 1:200)	Biolegend	Cat: 100421
CD125 PE-Cy7 (IL-5Rα; clone: DIH37; 1:200)	Biolegend	Cat: 153407
Lineage cocktail Pacific Blue (including CD3 (17A2), B220 (RA3-6B2), CD11b (M1/70), TER-119 321 (Ter-119), Gr-1(RB6-8C5); 1:200)	Biolegend	Cat: 133305
CD4 Pacific Blue (clone: GK1.5; 1:200)	Biolegend	Cat: 100427
CD8a Pacific Blue (clone: 53-6.7; 1:200)	Biolegend	Cat: 100728
CD11c Pacific Blue (clone: N418; 1:200)	Biolegend	Cat: 117321
NK1.1 Pacific Blue (clone: PK136; 1:200)	Biolegend	Cat: 108721
FcεRIα Pacific Blue (clone: MAR-1; 1:200)	Biolegend	Cat: 134313
IFN-γ Pacific Blue (clone: XMF1.2; 1:200)	Biolegend	Cat: 505817
CD193 BV421 (CCR3; clone: J073E5; 1:200)	Biolegend	Cat: 144517
CD90.2 APC (clone: 30-H12; 1:200)	Biolegend	Cat: 105311
CD127 APC (clone: A7R34; 1:200)	Biolegend	Cat: 135011
CD3 APC (clone: 17A2; 1:200)	Biolegend	Cat: 100235
**Software**		
GraphPad Prism v5.04	https://www.graphpad.com/	N/A
FlowJo X	https://www.flowjo.com/	N/A
IDEAS 6.2	Luminex Corporation	N/A
R 3.5.2 64-bit	https://www.r-project.org/	N/A
Metaboanalyst 3.0	https://www.metaboanalyst.ca/	N/A
nSolver v3	Nanostring	N/A
FIJI ImageJ 1.52n	https://fiji.sc/	N/A
Nikon NIS-Element	Nikon	N/A
