# Supplementary material for: Small extracellular vesicle signaling and mitochondrial transfer reprogram T helper cell function in human asthma
Source: Nat Commun. 2026 May 26;17:6842. doi: 10.1038/s41467-026-73684-y (PMC13389491; doi:10.1038/s41467-026-73684-y)
Supplement: Supplementary file 2 — Description of Additional Supplementary Files [file 41467_2026_73684_MOESM2_ESM.pdf]

Description of Additional Supplementary Files

Supplementary Video 1 - A z-stack of confocal images indicating co-localization (yellow) of the Mito-GFP+ sEVs with cytoplasmic actin in T cells.

Supplementary Video 2 - Live confocal imaging of Tubulin-RFP transduced and Mitoview-labeled T cells, co-cultured with Mito-GFP+ sEVs shows co-localization of Mito-GFP with the polarized cytoskeleton and mitochondrial network of recipient CD4+ T cells.
