# Supplementary material for: Small extracellular vesicle signaling and mitochondrial transfer reprogram T helper cell function in human asthma
Source: Nat Commun. 2026 May 26;17:6842. doi: 10.1038/s41467-026-73684-y (PMC13389491; doi:10.1038/s41467-026-73684-y)
Supplement: Supplementary file 5 — Reporting Summary [file 41467_2026_73684_MOESM5_ESM.pdf]

nature portfolio

Corresponding author(s): Jessy S. Deshane

Last updated by author(s): Apr 20, 2026

Reporting Summary

Nature Portfolio wishes to improve the reproducibility of the work that we publish. This form provides structure for consistency and transparency in reporting. For further information on Nature Portfolio policies, see our Editorial Policies and the Editorial Policy Checklist.

Statistics

For all statistical analyses, confirm that the following items are present in the figure legend, table legend, main text, or Methods section.

n/a	Confirmed	
☐	☒	The exact sample size (*n*) for each experimental group/condition, given as a discrete number and unit of measurement
☐	☒	A statement on whether measurements were taken from distinct samples or whether the same sample was measured repeatedly
☐	☒	The statistical test(s) used AND whether they are one- or two-sided<br>*Only common tests should be described solely by name; describe more complex techniques in the Methods section.*
☒	☐	A description of all covariates tested
☐	☒	A description of any assumptions or corrections, such as tests of normality and adjustment for multiple comparisons
☐	☒	A full description of the statistical parameters including central tendency (e.g. means) or other basic estimates (e.g. regression coefficient) AND variation (e.g. standard deviation) or associated estimates of uncertainty (e.g. confidence intervals)
☐	☒	For null hypothesis testing, the test statistic (e.g. *F*, *t*, *r*) with confidence intervals, effect sizes, degrees of freedom and *P* value noted<br>*Give P values as exact values whenever suitable.*
☒	☐	For Bayesian analysis, information on the choice of priors and Markov chain Monte Carlo settings
☒	☐	For hierarchical and complex designs, identification of the appropriate level for tests and full reporting of outcomes
☐	☒	Estimates of effect sizes (e.g. Cohen's *d*, Pearson's *r*), indicating how they were calculated

Our web collection on statistics for biologists contains articles on many of the points above.

Software and code

Policy information about availability of computer code

Data collection	FACSDiva software version 8.0.1 was used for flow cytometry data acquisition. sEV concentration and size distributions were determined using a NanoSight NS300 and a Spectradyne's nCS1 nanoparticle analyzer with Hardware Version 1. Cryo-EM data was acquired using a Glacios 2 electron microscope equipped with a Falcon 4i direct detector. Nanoimaging and quantitation of sEVs were performed using a ONi Nanoimager.
Data analysis	Flow cytometry data were analyzed with FlowJo (version 8.5.2; version 10; TreeStar) or IDEAS software (version 6.2). Spectradyne nCS1 data was analyzed with the nCS1 Data Viewer. Cryo-EM images were filtered and and scale bars were added using Fiji software by the core facility. Nanoimaging and quantitation of sEVs was completed using the CODI software by ONi. Differential gene expression analysis was performed using nSolver version 3. Data were plotted with GraphPad Prism 5.04, Metaboanalyst 3.0, or R 331 3.5.2 (64-bit). The NanoString custom panel gene expression data has been deposited in the Gene 339 Expression Omnibus (GEO) under accession ACCESSION# GSE144813.

For manuscripts utilizing custom algorithms or software that are central to the research but not yet described in published literature, software must be made available to editors and reviewers. We strongly encourage code deposition in a community repository (e.g. GitHub). See the Nature Portfolio guidelines for submitting code & software for further information.

Data

Policy information about availability of data

All manuscripts must include a data availability statement. This statement should provide the following information, where applicable:

- Accession codes, unique identifiers, or web links for publicly available datasets
- A description of any restrictions on data availability
- For clinical datasets or third party data, please ensure that the statement adheres to our policy

All data are included in the Supplementary Information or available from the authors, as are unique reagents used in this article. The raw numbers for charts and graphs are available in the Source Data file whenever possible. The NanoString custom panel gene expression data has been deposited in the Gene Expression Omnibus (GEO) under accession #GSE144813 (https://www.ncbi.nlm.nih.gov/geo/query/acc.cgi?acc=GSE144813) and # GPL28122 (https://www.ncbi.nlm.nih.gov/geo/query/acc.cgi?acc=GPL28122).

Research involving human participants, their data, or biological material

Policy information about studies with human participants or human data. See also policy information about sex, gender (identity/presentation), and sexual orientation and race, ethnicity and racism.

Reporting on sex and gender	All available samples from consented patients were utilized for the described study, with equal recruitment of men and women. Sex and Gender were not adjusted for due to sample size. For the healthy control group 36.36% of participants were male and for the asthmatic group 19.04% of patients were male (see Table 1).
Reporting on race, ethnicity, or other socially relevant groupings	No reporting on race, ethnicity, or socially relevant groupings was included in this mansucript.
Population characteristics	Population characteristics are detailed in Table 1. Participant median age was 44 for the healthy control group and 40 for the asthmatic group. Median IgE Titer, predicted % FEV1, and median eosinophils significantly varied between control and asthmatic groups.
Recruitment	Asthmatic and healthy control subjects were enrolled through the University of Alabama at Birmingham Lung Health Center and screened for IgE titer, blood eosinophil frequencies, past medical history, and FEV1 (Table I). Healthy controls did not have histories of asthma, pulmonary infections or other known lung diseases. All asthmatic patients had a prior diagnosis of asthma and demonstrated a 12% or greater increase in FEV1 within 30 minutes of administrating 400 µg of albuterol, as outlined in the GINA guidelines (Global Strategy for Asthma Management and Prevention; http://www.ginasthma.org/). Screened subjects with serum cotinine levels greater than 10ng/ml (smokers) and those subjects who received treatment with inhaled or systemic corticosteroids within the six weeks prior to or during the study were excluded. Previous exposure to secondhand smoke was determined by LC-MRM mass spectrometry for serum cotinine levels between 0.05-10 ng/ml. Healthy controls and asthmatics with serum cotinine levels below 0.05 ng/ml were considered non-SHS-exposed subjects.
Ethics oversight	The study was approved by the University of Alabama at Birmingham Institutional Review Board (Protocol IRB-151209005), and written informed consent was obtained from all participants.

Note that full information on the approval of the study protocol must also be provided in the manuscript.

Field-specific reporting

Please select the one below that is the best fit for your research. If you are not sure, read the appropriate sections before making your selection.

☒ Life sciences ☐ Behavioural & social sciences ☐ Ecological, evolutionary & environmental sciences

For a reference copy of the document with all sections, see nature.com/documents/nr-reporting-summary-flat.pdf

Life sciences study design

All studies must disclose on these points even when the disclosure is negative.

Sample size	Sample size for this study was based on sample availability during the study period. As the methodology described is novel and statistical evaluation showed meaningful differences between comparisons, the sample size was sufficient for the study.
Data exclusions	Outlier analysis was performed on the data shown in Figure 1E. Identified outliers were excluded from the graph. The source data file denotes the identified outliers.
Replication	Independent experiments were performed to ensure reproducibility of experimental findings. Biological heterogeneity between tissue samples was expected, therefore biological replicates were completed for each study group. Technical replicates were used in cytokine ELISA, qRT-PCR, Western blots, ImageStream flow, nanoimaging, particle analyses, co-culture experiments, flow cytometry analysis and averages were reported when applicable.
Randomization	Experimental groups were determined based on physician determined asthma status. and inclusion and exclusion criteria. Randomization was not performed for recruitment.

Blinding	Human subject recruitment and sample collection were not blinded. Experiments for 3 main figures with human cells and sEVs were performed by one individual and data analyzed by two other individuals. Animal experiments were not blinded and a team were involved in data collection and analyses. For histological analyses, animal experiments were performed by one individual and histological imaging analyses was blinded to two other individuals. Once the final images were acquired, they were unblended. Western blots were performed and repeated by different individuals.

Reporting for specific materials, systems and methods

We require information from authors about some types of materials, experimental systems and methods used in many studies. Here, indicate whether each material, system or method listed is relevant to your study. If you are not sure if a list item applies to your research, read the appropriate section before selecting a response.

Materials & experimental systems

n/a	Involved in the study
☐	☒ Antibodies
☒	☐ Eukaryotic cell lines
☒	☐ Palaeontology and archaeology
☐	☒ Animals and other organisms
☐	☒ Clinical data
☒	☐ Dual use research of concern
☒	☐ Plants

Methods

n/a	Involved in the study
☒	☐ ChIP-seq
☐	☒ Flow cytometry
☒	☐ MRI-based neuroimaging

Antibodies

Antibodies used	CD11b APC Cy7 (1:200, clone: ICRF44, BD Biosciences, Cat. #:17-0118-42); CD169 BV510 (1:200, clone: 7-239, BD Biosciences, Cat. #:742992); HLA-DR APC (1:200, clone: LN3, eBioscience, Cat. #:17-9956-42); CD163 PE (1:200, clone: eBioGHI/61, eBioscience, Cat. #:A15792); CD33 PE-Cy7 (1:200, clone: WM53, eBioscience, Cat. #:25-0338-42); CD14 PerCp-Cy5.5 (1:200, clone: 61D3, eBioscience, Cat. #: 45-0149-42); CD11c PE-Cy5 (1:200, clone: 3.9, eBioscience, Cat. #:15-0116-42); CD63 eFlour450 (1:200, clone: H5C6, Affymetrix, Inc., Cat #: 48-0639-42); HLA-DR APC (1:200, clone: LN3, Affymetrix, Inc., Cat. #:17-9956-42); CD54 PE (1:200, clone: 86 HA58, Affymetrix, Inc., Cat. #:12-0549-42); CD9 PE (1:200, clone: M-L13, BD Biosciences, Cat. #:341637); CD81 PE-Cy7 (1:200, clone 5A6, BioLegend, Cat. #:349511); TSG101 Alexa Fluor 647 (1:200, clone: 4A10, Novus Biologicals, Cat. No:NB200-112AF647); CD4 PE-Cy7 (1:200, clone: SK3, ThermoFisher, Cat. #:25-0041-81); IL-4 PE (1:200, clone: 8D4-8, ThermoFisher, Cat. #:12-7049-42); IL-17A APC (1:200, clone: eBio64DEC17, ThermoFisher, Cat. #:17-7179-42): IFNγ BV421 (1:200, clone: B27, BD Horizon, Cat. #:562988), CD69 eFluor450 (1:200, clone: FN50, 120 ThermoFisher, Cat. #:48-0699-41): CD154 APC (1:200, clone: 24-31, ThermoFisher, Cat. #:17-1548-41); pZap70 Alexa Fluor 647 (1:200, clone: 17A/P-ZAP70, BD Phosflow, Cat. #:557817); α-tubulin eFluor 615 (1:200, clone DM1A, ThermoFisher, Cat. #:42-4502-82); LFA-1 (1 μg/mL, clone: R7.1, Fisher Scientific, Cat. #:50-176-82); Pan-HLA (10 μg/mL, DR/DP/DQ, clone: TU39, BD Pharmigen, Cat #:555557); Drp1 (1:1000; Clone: 8; BD Bioscience, Cat #: 611113); MHC-II eFlour450 (1:200, clone: M5/114/15.2, Life Technologies, Cat. #48-5321-82); CD63 APC (1:200, clone: NVG2, Life Technologies, Cat. #17-0631-82); CD81 PE (1:200, clone: Eat-2, Biolegend, Cat. #:104905); CD9 PE-Cy7 (1:200, clone: MZ3, Biolegend, Cat. #:124816); CD45 PE (1:200, clone: 30-F11, Life Technologies, Cat. #12-0451-82); CD4 PE-Cy7 (1:200, clone: GK1.5, Life Technologies, Cat. #25-0047-41); CD69 eFluor450 (1:200, clone: H1.2F3, Life Technologies, Cat. #); Gr-1 PE (1:200, clone: RB6-8C5, Life Technologies, Cat #:12-5931-82); CD25 PE (1:200, clone: PC61.5, Life Technologies, Cat #:12-0251-82); CD62L PE (1:200, clone: MEL-14,Life Technologies, Cat #:12-0621-82); CD206 APC (1:200, clone: MR6F3, Life Technologies, Cat #:17-2061-82); IL-4 APC (1:200, clone: 11B11, Life Technologies, Cat #:17-7041-81); Ly6C PerCp-Cy5.5 (1:200, clone: HK1.4, Life Technologies, Cat #:45-5932-82); CD170 PerCp-eFluor 710 (Siglec F, 1:200, clone: 1RNM44N, Life Technologies, Cat #:46-1702-80); MHC-II PE-Cy5 (I-A/I-E, 1:200, clone: M5/114.15.2, Life Technologies, Cat #:15-5321-82); CD4 PE-Cy7 (1:200, clone: GK1.5, Life Technologies, Cat #:25-0041-81); F4/80 BV605 (1:200, clone: T45-2342, BD Biosciences, Cat #:743281); CD11b APC-Cy7 (1:200, clone: M1/70, BD Biosciences, Cat #:557657); CD3 APC-Cy7 (1:200, clone: 145-2C11,BD Biosciences, Cat #:557596); Ly6G AlexaFluor 700 (1:200, clone: 1A8, BD Biosciences, Cat #:561236); CD101 AlexaFluor 647(Igsf2, 1:200, clone: 307707,BD Biosciences, Cat #:564473); CD278 PE (ICOS, 1:200, clone: C398.4A, Biolegend, Cat. #:313507); IL-17A PE (1:200, clone: TC11-18H10.1,Biolegend, Cat. #:506903); IL-33Rα PerCp-Cy5.5 (IL1RL1, 1:200, clone: DIH9, Biolegend, Cat. #:145311); CD45 PE-Cy7 (1:200, clone: 30-F11,Biolegend, Cat. #:103113); CD4 PE-Cy7 (1:200, clone: GK1.5,Biolegend, Cat. #:100421); CD125 PE-Cy7 (IL-5Rα, 1:200, clone: DIH37, Biolegend, Cat. #153407:); lingeage cocktail Pacific Blue (including CD3 (17A2), B220 (RA3-6B2), CD11b (M1/70), TER-119 321 (Ter-119), Gr-1(RB6-8C5), 1:200, Biolegend, Cat. #:133305); CD4 Pacific Blue (1:200, clone: GK1.5,Biolegend, Cat. #:100427); CD8a Pacific Blue (1:200, clone: 53-6.7, Biolegend, Cat. #:100728); CD11c Pacific Blue (1:200, clone: N418, Biolegend, Cat. #:117321); NK1.1 Pacific Blue (1:200, clone: PK136,Biolegend, Cat. #:108721); FcεRIα Pacific Blue (1:200, clone: MAR-1, Biolegend, Cat. #:134313); IFN-γ Pacific Blue (1:200, clone: XMF1.2, Biolegend, Cat. #:505817); CD193 BV421 (CCR3, 1:200, clone: J073E5, Biolegend, Cat. #:144517); CD90.2 APC (1:200, clone: 30-H12, Biolegend, Cat. #:105311); CD127 APC (1:200, clone: A7R34, Biolegend, Cat. #:135011); CD3 APC (1:200, clone: 17A2, Biolegend, Cat. #:100235); NF-κB p65 BV421 (1:200, clone: K10-895.12.50, BD Biosciences, Cat. #:565446); CD81 (1:500, clone: D3N2D, Cell Signaling, Cat. #: 56039); Tim23 (1:1000, clone: polyclonal, Proteintech, Cat. #: 11123-1-AP); Tomm20 (1:200, Abcam, Cat #:56783), CD4 PE (1:200, clone:RPA-T4, Invitrogen, Cat. #:12-0049-42); IL-17A APC (1:200, clone: eBio64Dec17, Thermo Fisher Scientific, Cat #: 17-7179-42), and IL-4 PerCP-Cy5.5 (1:200, clone:8D4-8, BD Biosciences, Cat. #: 561234).
Validation	Antibodies were chosen based on manufacturers published statements and citations, and further confirmed with adequate flow cytometry controls.

Animals and other research organisms

Policy information about studies involving animals; ARRIVE guidelines recommended for reporting animal research, and Sex and Gender in Research

Laboratory animals	Female C57BL/6J mice at 6 to 8 weeks of age were purchased from The Jackson Laboratory (Stock #: 000664) and Mito-QC mice obtained on an MTA. Mice were maintained in a conventional pathogen-free housing facility under standard conditions and handled in accordance with the Guidelines for Animal Experiments at the University of Alabama at Birmingham. Experimental and control animals were maintained in separate cages but co-housed in the same space within the animal facility.
Wild animals	Study did not involve wild animals.
Reporting on sex	Female mice were utilized for this study as this best represented the human asthmatic population who were recruited for the study. Mito-QC mice obtained was a mixture of female and male mice. We did not see sex differences in our sEV transfer experiments. Sex differences in mitochondrial fission and packing of mitochondria into sEVs has not been identified so far in mice or humans. Sex differences in BALF sEVs have also not been reported so far. A much larger sample size would be required to assess sex as a biological variable in these biological processes.
Field-collected samples	Study did not involve samples collected from the field.
Ethics oversight	All studies were completed in accordance with the Guidelines for Animal Experiments at the University of Alabama at Birmingham with approved animal protocols (Animal Protocol Numbers: 20273, 21974).

Note that full information on the approval of the study protocol must also be provided in the manuscript.

Clinical data

Policy information about clinical studies

All manuscripts should comply with the ICMJE guidelines for publication of clinical research and a completed CONSORT checklist must be included with all submissions.

Clinical trial registration	*Provide the trial registration number from ClinicalTrials.gov or an equivalent agency.*
Study protocol	*Note where the full trial protocol can be accessed OR if not available, explain why.*
Data collection	*Describe the settings and locales of data collection, noting the time periods of recruitment and data collection.*
Outcomes	*Describe how you pre-defined primary and secondary outcome measures and how you assessed these measures.*

Plants

Seed stocks	N/A
Novel plant genotypes	N/A
Authentication	N/A

Flow Cytometry

Plots

Confirm that:

- [x] The axis labels state the marker and fluorochrome used (e.g. CD4-FITC).
- [x] The axis scales are clearly visible. Include numbers along axes only for bottom left plot of group (a 'group' is an analysis of identical markers).
- [x] All plots are contour plots with outliers or pseudocolor plots.
- [x] A numerical value for number of cells or percentage (with statistics) is provided.

Methodology

Sample preparation	Autologous peripheral CD4+ T cells were blocked for 30 min in sEV-depleted RPMI 1640 with 10% human AB serum. Cells were fixed and permeabilized using BD Cytoperm/Cytofix Kit (when appropriate), followed by antibody staining. Murine Lung

tissues were harvested and digested with collagenase B. Red blood cells were removed by ACK lysis buffer. Fc receptors were blocked with 3% BSA in PBS containing 2.4G2 antibody (anti-mouse CD16/CD32; BD Pharmingin), followed by antibody staining.

Instrument	LSR-II flow cytometer (Becton Dickinson) or Amnis ImageStream
Software	FlowJo (version 8.5.2; TreeStar) for flow cytoemtry data, and IDEAS software (version 6.2) for ImageStream data
Cell population abundance	For flow cytometry analysis, a minimum of 100,000 events were captured to obtain meaningful data for cell populations. The abundance of relevant cell populations was assessed by gating based on specific markers, ensuring accurate identification. For extracellular vesicles (EVs), a minimum of 5000-10,000 events were captured on the ImageStream to ensure robust analysis. The purity of the samples was confirmed by analyzing the gated populations, with the target cell populations typically exceeding 95%.
Gating strategy	Cells were first gated based on forward scatter (FSC) and side scatter (SSC) to exclude debris and doublets. Single cells were then identified using FSC-A vs. FSC-H. Thresholds for positive cells were determined post-compensation using single-stained color controls, isotypes as needed and unstained controls to accurately distinguish positive from negative populations. For extracellular vesicle (EV) analysis, the ApogeeBead mix was used to determine size based on scatter and to match the fluorescence tags of the beads with the scatter. Spectradyne analyses of extracellular vesicles included cartridge based size exclusion followed by gating based on median size determined by the analyzer and the controls provided by the analyzer. All gating strategies are included in the main figures and supplementary figures. Mito-GFP and Mito-RFP positive cells were identified by gating on negative and positive controls for lentiviral transduced cells. T cells positive for Mito-GFP+ and Mito-RFP + vesicles were gated based on negative and positive controls in co-culture experiments.

☒ Tick this box to confirm that a figure exemplifying the gating strategy is provided in the Supplementary Information.
