# Supplementary material for: Small extracellular vesicle signaling and mitochondrial transfer reprogram T helper cell function in human asthma
Source: Nat Commun. 2026 May 26;17:6842. doi: 10.1038/s41467-026-73684-y (PMC13389491; doi:10.1038/s41467-026-73684-y)
Supplement: Supplementary file 6 — Transparent Peer Review file [file 41467_2026_73684_MOESM6_ESM.pdf]

nature portfolio

Peer Review File

Small Extracellular Vesicle Signaling and Mitochondrial Transfer Reprograms T Helper Cell Function in Human Asthma

Corresponding Author: Dr Jessy Deshane

Parts of this Peer Review File have been redacted as indicated to maintain the confidentiality of unpublished data.

This file contains all reviewer reports in order by version, followed by all author rebuttals in order by version.

Version 0:

Reviewer comments:

Reviewer #1

(Remarks to the Author)
This is a well-designed and executed study with a defined goal of establishing the mechanism by which sEVs play a role in immune regulation of asthma. The authors focus on fragmented mitochondria in the sEVs as mediators of immune cell proliferation and differentiation. Authors use human patient-derived T cells for in vitro studies and corroborate their findings using a murine model of asthma. The study findings are valuable in advancing our understanding of immunopathogenesis of asthma. Following are a few comments for the authors to consider.
Figure 1A-D: sEVs are not well characterized to confirm the expression of Class II MHC. Fig 1E data are not very convincing. Fig 1F data needs to be validated using flow cytometry or western blotting. For transcriptomics data, the authors have used only Th17 differentiation and not Th2 differentiation or CD4+ T cell proliferation phenotype augmented by sEVs.
Fig 4 and 5 rely (few other datasets) entirely on the expression of markers after treatment with different inhibitors. These data could be strengthened by measuring cytokine release or some other functional readouts.
Drp1-mediated and mitochondrial fission responsible for mitochondrial packaging in sEV is explored in this study. However, the data seem to suggest that Drp1-fission-mediated packaging is only a small portion of mitochondrial packaging as the packaging is not eliminated in knockdown cells.
Mitochondrial DAMP signaling involved in the immune activation muddles the key message of the manuscript. How rest of the data showing mitochondrial transfer into cells directly activate T cells reconcile with DAMP?
In vivo studies using OVA mice lack complete characterization of asthma phenotype. The authors primarily show the effect on airway inflammation.

Reviewer #2

(Remarks to the Author)
In this manuscript, Hough et.al. identified a pathological role of airway MDRC-derived sEVs in asthmatics with extensive ImageStream data. The authors demonstrated that only asthmatic MDRC-derived sEVs contain dysfunctional mitochondria (may also called mitovesicles), which are indispensable for triggering T cell activation and Th cell polarization. They also explored the mechanisms underlying the secretion and uptake of these mitovesicles. They showed that the secretion of mitovesicles is drp1-dependent, and the uptake is mediated by membrane fusion. Importantly, they modeled the airway inflammation in animal models by intranasal administration of these mitovesicles. In general, this study is of great interest and significance. Several comments need to be addressed.
1. Lack of basic EV characterization. The authors used a Total Exosome Isolation kit to isolate EVs, which usually co-precipitate a lot of other extracellular compartments, including small cell debris, large EVs, ribonucleoproteins, etc. The authors need to provide rigorous evidence demonstrating that the isolated ‘EVs’ are sEVs. This includes size distribution, zeta potential, electron microscopy, and immunoblotting of classical EV markers, mitochondrial markers (confirming the presence of mitochondria in asthmatic EVs), and cellular (Golgi) markers (excluding cell debris contamination). The authors used a mito-GFP marker to determine mitochondria-containing sEVs, which needs additional electron microscopy images to show the presence and morphology of mitochondria in sEVs.

2. ImageStream that combines flow cytometry with fluorescence imaging technology may be used to analyze large EVs but not small EVs precisely. The author could determine this by comparing the events/volume detected by ImageStream and the nanoparticle number measured by Nanosight. Also, please present the average size and smallest size of detected EVs by ImageStream.
3. The authors chose a very low dose of EVs for the treatments, both in vitro and in vivo, compared with most current EV studies. A ratio of 1:10 (T cells:sEVs) was used to treat T cells, and 1x10e8 was used to treat mice. Could the authors provide a rationale for choosing these doses? Also, are there dose-dependent effects of these EVs in vitro and in vivo?
4. The authors showed the presence of dysfunctional mitochondria in MDRC-derived EVs from asthmatic patients. Is there any mitochondrial dysfunction in MDRCs among healthy and asthmatic patients?
5. The authors showed that blockage of MHC-II inhibited the EV-mediated T cell activation without affecting their internalization. However, they also showed that EV-mediated T-cell activation is dependent on ROS produced by dysfunctional mitochondria upon internalization. Would blockage of MHC-II inhibit/prevent the release/generation of ROS from these EV-transported mitochondria? Please discuss the potential mechanism.
6. The authors used two methods for isolating EVs. Ultracentrifugation was used to isolate sEVs from BALF, and the Total Exosome Isolation kit was used to isolate sEVs from the MDRC conditional medium. Please explain the reasons.
7. Some typos to be corrected. Line 172, “CD81” should be “CD81+”. Line306, it should be “with unchanged” or “without changed”. Line 677, “E” should be “G”
8. Figure 2J should be placed before Figure 2F-I.

Guoping Li

Reviewer #3

(Remarks to the Author)
Hough and colleagues report in this manuscript that myeloid-derived regulatory cells (MDRCs) can secrete small extracellular vesicles (sEVs) that control CD4+ T cell responses in asthma. They found that BALF sEVs from asthmatic patients promote CD4+ T cell proliferation and Th2/Th17 immune responses. Interestingly, MDRC-derived sEVs contain similar amounts of mitochondria (Mito) in both healthy controls and asthmatic patients, but sEVs from asthmatics exhibited elevated levels of mitochondrial membrane potential. These sEVs can enter CD4+ T cells through membrane fusion at the site of cytoskeletal polarization and mitochondrial trafficking. This fusion is mediated by dynamin and requires sEV-derived MHC-II, mitochondrial complex III/V activity, and reactive oxygen species (ROS) to activate CD4+ T cells. Additionally, DRP1 appears to regulate mitochondrial packaging in Mito-sEVs. Intranasal transfer of Mito-sEVs also exacerbates the asthmatic immune response. Overall, while this work presents some interesting findings, it is very difficult to read due to a lack of clear logical flow and poor organization. The manuscript would benefit from restructuring and consolidating the data presentation to improve readability. The major concerns are technical concerns regarding the imaging and flow cytometry, which will need to be carefully addressed, before solid conclusions can be drawn from these experiments.

Concerns and Comments:

1. Technical Concerns with Imaging: The ability of imaging to determine co-expression of multiple markers, particularly cytokine expression, is a major technical concern. Consider verifying "co-expression" at the population level using flow cytometry.

2. Flow Cytometry Data Quality: The flow cytometry data lacks proper control groups, appears to be improperly compensated, and may have incorrect gating. These issues need to be addressed.

3. Logical Flow: The manuscript needs a better logical flow. Summarize the data more effectively, rather than randomly assigning them slots in the main figures and repeatedly presenting similar data in different formats.

4. Figure Presentation: Many figures of the same grouping are presented in different formats, making them busy and difficult to follow. For example, Fig. 1A, C, D, E, and F could follow the same format. Sub-figures are too close to each other, making it difficult to locate legends. Figures addressing similar questions and using the same experimental strategy should be grouped together.

5. Figure Legends: Figure legends do not need to repeat the methods in great detail, such as the dose of IL-2 in stimulation and the flow cytometry machine model.

6. Endo-RFP+ Cells: The Endo-RFP+ cells are presented throughout the main figures, but it was determined that the endosome pathway is minimally involved in CD4+ activation by sEVs. It is suggested that the key figure on this finding be retained in the main text, while the rest of the Endo-RFP data should be moved to the supplementary section to improve readability.

7. Figure 2: Panels E and J are representative figures of labeling, while panels C, D, F-I quantify the effects of sEVs from control and asthmatic BALF. Panels E and J can be moved to the supplementary section. Panels C, D, F-I should have consistent formats.

8. Figure 3: This figure is confusing. The functions of Mito-GFP and Endo-RFP should be described (e.g., "to label XX with XX, and XX with XX"). The pie chart in panel 3B has confusing colors that should be annotated, and the colored legends should intersect with panel 3C. Panel 3D can be moved to the supplementary section. Additionally, why is panel 3B

needed? It is an essential part of panel 3C, but presented differently. Not every experiment needs to be included—consider consolidating the data and presenting a comprehensive summary.

9. Figure 4: Similar issues are present as in Figure 3.

10. Combining Figures: Figures 3, 4, and 5A-B could be combined, as they share the same schematics with different inhibitors. All of these address the required sEV factors in CD4+ activation.

11. Figure 5E (right): Controls should include multiple replicates from healthy and asthmatic samples. The validation of knockdown (KD) should be presented before the functional data. Panel 5E (left) is a summary of panel 5F.

12. Mitochondrial Fission Section: This section could be a standalone figure and should precede Figures 3, 4, and 5A-B, as it describes how Mito-sEVs are packaged.

13. Figure 6: This figure can be merged with Figure 2, as both analyze how MDRC-derived sEVs enter CD4+ T cells using the same experimental strategy.

14. Figure 7: Move panel 7G, which verifies imaging techniques, to the supplementary section, and move panels 7H-J, which analyze allogenic effects, to the supplementary section as well. Panels K-P should test Mito-GFP+ sEVs and should not claim their mechanism as DAMP-mediated. Instead, this data suggests that sEVs can promote both innate and adaptive inflammatory responses associated with asthma.

15. Figures 8 and 10: These figures need experimental schematics and an OVA-sensitized + non-sEV control group. The gating strategies shown in Figures S10 and S11 are concerning, suggesting that the flow data were not properly compensated, and most of the markers were gated on backgrounds rather than actual signals. Please note that cells from inflamed airways tend to be larger and exhibit higher background signals in flow cytometry. The gating strategy should include FSC, SSC, singlets, viability, and markers. The gating strategy for the myeloid cells should be included as well.

16. Th2/Th17 Response in OVA Model: The OVA model is Th2-biased. The high response of Th17 may be an artifact of the flow gating. To verify this, analyze BALF for Th2 and Th17 cytokines by ELISA.

17. Lung Histology: The lung histology of the asthmatic mouse model should be presented to reveal the effects of sEVs in asthma.

Reviewer #4

(Remarks to the Author)
In this study, Hough et al. found that airway Myeloid-derived regulatory cells (MDRCs) from asthmatics can transfer mitochondria through Small extracellular vesicles (sEVs) via membrane fusion. This is associated with T cell activation and Th17 and Th2, but not Th1 cell differentiation. sEV-derived mitochondria co-localized with immune synapse and their fission and function affected the functions of recipient T cells and related responses. These findings could be of interest. Addressing the following comments may help to improve the current study.

It is important to reveal what are the critical signaling pathways activated by sEV with or without rotenone to lead to T cell activation, especially only for Th2, Th17 but not for Th1 cells. In addition, it is important to address if sEV-mediated effects are different in naïve vs effector vs Treg cells.

Direct evidence of increased mitochondrial fission in asthmatics sEVs should be provided. In addition, it should be addressed if Drp1 mutants that cannot oligomerize affect mitochondrial fission in sEVs and the function of sEVs in T cells.

Direct evidence of sEVs affecting the organization of the synapse through cytoskeletal and mitochondrial reorganization and related downstream signaling should be provided. In addition, the relationship between mitochondrial functon(ATP, ROS, fission) in the sEVs and synapse formation and downstream signaling in T cells needs further investigation and elaboration.

For mouse study, it is unclear if the observations were due to a direct sEV-mediated effect on CD4 T cells or also due to an indirect, sEV-mediated effect on other immune or non-immune cells. Can sEV containing/conditioned CD4 T cells promote disease in mice on their own?

Fig. 1A-1B, FACS plots of CFSE dilution should be shown. The % of proliferating cells are small. Another method, e.g. BrdU incorporation assays, should also be used to evaluate T cell proliferation.

Fig. 1C, 1D and S1A, why less than 2% activated T cells expressed CD69 and pZap70 (Fig. 1D), while more than 40% T cell proliferated? A co-identification of CFSE, CD69, CD154, CD25 should be done to correlate TCR activation and T cell proliferation.

The difference between + vs. – sEVs of IL4 and IL17 protein production was about 2 folds per flow-cytometry (Fig. 1E and Fig. S1B), yet the mRNA expression was about 10 folds. What accounts for such a big difference?

Fig. 1G, GATA3 was downregulated in + sEVs samples, why Th2 was upregulated in these samples? Were Th17 specific

gene signatures, including Ror, preferentially upregulated in + sEVs samples?

Fig. S3A. T cell proliferation assays as in Fig. 1D should also be performed to assess T cell activation, as the readout could be more robust.

Version 2:

Reviewer comments:

Reviewer #1

(Remarks to the Author)
Thanks for a thorough revision of the manuscript.

Reviewer #2

(Remarks to the Author)
The authors addressed my concerns. No further comments.

Reviewer #3

(Remarks to the Author)
The authors propose that airway myeloid-derived regulatory cells (MDRCs) in asthma secrete small extracellular vesicles (sEVs) that transfer mitochondria-related signals to CD4 T cells, driving T cell proliferation and Th2/Th17 polarization via mitochondrial oxidant–dependent NF-κB signaling. They further argue that internalized sEV/mitochondrial signal localizes with polarized cytoskeleton/mitochondrial networks in recipient T cells, that DRP1-promoted mitochondrial dynamics contribute to mitochondrial packaging into sEVs, and that intranasal transfer of MDRC-derived sEVs exacerbates allergic airway inflammation in vivo.

The revision addresses some of my previous comments concerning repeated presentations of the same data, potential misinterpretation of co-expression of multiple markers based solely on imaging data, the lack of flow data quality control and gating, and data consolidation (some of them). The authors seem to prefer detailed (most of the time repeated) presentation of methods and similar datasets, which may be further consolidated in the overall research presentation. Currently, the manuscript appears long, which may not be necessary. For example, the asthmatic phenotyping data were spread into three main text figures. The reviewer hopes that the authors reconsider combining subfigures for the asthma experimental workflow, cell characterization, histology images (with scores) into one main figure.

Beyond formatting, I have the following major questions:

1. It is not surprising to see significant roles of MDRC/sEV MHC-II and mitochondrial activity in Th2/Th17 differentiation. However, it is unlikely that these could be therapeutic targets to attenuate pathogenic T helper cells during asthma and other related diseases. Can blocking/inhibiting sEVs or sEV-specific signals attenuate asthmatic responses?

2. The authors did not test whether MHC-II blockade alters mitochondrial ROS generation/release or other sEV characteristics that are directly relevant to their roles in regulating T cells.

3. Given that no viability dye was included in the flow cytometric analyses, and that the cytokine staining signals in the highly inflamed lung samples appeared dim. It is possible that some dead cells, which tend to show autofluorescence in flow cytometry, were included in the analyses and biased the conclusions. To ensure that Th2/Th17 responses are indeed enhanced in the asthma model, additional validation, such as cytokine ELISA, should be considered.

4. The method section listed flow antibodies for Treg cells, but no Treg-related data were presented. What happened to the Treg cells?

Reviewer #4

(Remarks to the Author)
My concerns are addressed.

Open Access This Peer Review File is licensed under a Creative Commons Attribution 4.0 International License, which permits use, sharing, adaptation, distribution and reproduction in any medium or format, as long as you give appropriate credit to the original author(s) and the source, provide a link to the Creative Commons license, and indicate if changes were made.
In cases where reviewers are anonymous, credit should be given to 'Anonymous Referee' and the source.
The images or other third party material in this Peer Review File are included in the article’s Creative Commons license, unless indicated otherwise in a credit line to the material. If material is not included in the article’s Creative Commons license and your intended use is not permitted by statutory regulation or exceeds the permitted use, you will need to obtain permission directly from the copyright holder.
To view a copy of this license, visit https://creativecommons.org/licenses/by/4.0/

Response to Reviewers:

Reviewer #1

This is a well-designed and executed study with a defined goal of establishing the mechanism by which sEVs play a role in immune regulation of asthma. The authors focus on fragmented mitochondria in the sEVs as mediators of immune cell proliferation and differentiation. Authors use human patient-derived T cells for in vitro studies and corroborate their findings using a murine model of asthma. The study findings are valuable in advancing our understanding of immunopathogenesis of asthma. Following are a few comments for the authors to consider.

Response: We are grateful to the reviewer for these comments and provide a point-by-point response below.

Comment 1: Figure 1A-D: sEVs are not well characterized to confirm the expression of Class II MHC.

Response: We appreciate this comment and provide data in Supplementary Figure 1 showing Class II MHC expression in sEVs. Previously, we characterized BALF EVs and reported on Class II expression in PMID: 29986209.

Comment 2: Fig 1E data are not very convincing. Fig 1F data needs to be validated using flow cytometry or western blotting.

Response: We recognize that we didn't provide adequate supporting data previously. We now provide flow cytometry plots (contour with outliers) and gating strategies with additional representative plots for all Th polarization studies. These are provided in Main Figures 1 and 7 as well as Supplementary Figures 3, 4, 12 and 13. Additionally, we provide cytokine measurements from our in vitro studies (Figure 1) and in vivo studies (Figure 10).

Comment 3: For transcriptomics data, the authors have used only Th17 differentiation and not Th2 differentiation or CD4+ T cell proliferation phenotype augmented by sEVs.

Response: We appreciate this comment and have provided additional data for RORC and GATA3 expression in Figure 1. RORC expression significantly changed with sEV-co-culture with T cells in asthmatics. We saw an increase in expression for GATA3, but this did not reach significance. Similar human sample to sample variability was seen in our transcriptomic data for GATA-3. But our Th2 cytokine data are supportive of Th2 differentiation.

Comment 4: Fig 4 and 5 rely (few other datasets) entirely on the expression of markers after treatment with different inhibitors. These data could be strengthened by measuring cytokine release or some other functional readouts.

Response: We appreciate this comment from the reviewer. In the studies with rotenone, we performed experiments to further evaluate the role of NF-kB signaling in the effect we see with rotenone and have quantitated RELA expression as RELA and NF-kB signaling are both involved in Th differentiation. These results are now shown in Supplementary Figure S10.

Comment 5: Drp1-mediated and mitochondrial fission responsible for mitochondrial packaging in sEV is explored in this study. However, the data seem to suggest that Drp1-fission-mediated packaging is only a small portion of mitochondrial packaging as the packaging is not eliminated in knockdown cells.

Response: We agree that, although significant reduction in sEV packaging is achieved, it is not eliminated in knockdown cells. We are not achieving complete knockdown with siRNA pool in multiple samples; the elimination may be enhanced if this was achieved. Additional unexplored mechanisms may be involved.

Comment 6: Mitochondrial DAMP signaling involved in the immune activation muddles the key message of the manuscript. How rest of the data showing mitochondrial transfer into cells directly activate T cells reconcile with DAMP?

Response: We agree with the reviewer that based on our current data, we can only claim that sEVs promote both innate and adaptive immune responses; these may be independent of DAMP signaling.

Comment 7: In vivo studies using OVA mice lack complete characterization of asthma phenotype. The authors primarily show the effect on airway inflammation.

Response: We agree with the reviewer that we have not performed lung function studies with flexivent in our in vivo experiments. We have provided OVA IgE measurements in BALF and serum and readouts of Muc5AC in BALF. MitoQC mice were obtained on an MTA with investigators from Australia. This required us for these mice to be bred, genotyped and provided by investigators on the MTA done with non-NIH and non-foundation grant funds. Our sensitization and challenge experiments had to be performed on only institutional funds. We regret that this limited us from maintaining a large colony with enough animals ourselves or obtaining enough numbers of animals to perform cell sorting from sensitized and challenged animals as well as flexivent studies and additional extensive histologic evaluations.

Reviewer #2:

In this manuscript, Hough et.al. identified a pathological role of airway MDRC-derived sEVs in asthmatics with extensive ImageStream data. The authors demonstrated that only asthmatic MDRC-derived sEVs contain dysfunctional mitochondria (may also called mitovesicles), which are indispensable for triggering T cell activation and Th cell polarization. They also explored the mechanisms underlying the secretion and uptake of these mitovesicles. They showed that the secretion of mitovesicles is drp1-dependent, and the uptake is mediated by membrane fusion. Importantly, they modeled the airway inflammation in animal models by intranasal administration of these mitovesicles. In general, this study is of great interest and significance. Several comments need to be addressed.

Response: We are grateful to the reviewer for these comments and provide a point-by-point response below.

Comment: 1. Lack of basic EV characterization. The authors used a Total Exosome Isolation kit

to isolate EVs, which usually co-precipitate a lot of other extracellular compartments, including small cell debris, large EVs, ribonucleoproteins, etc. The authors need to provide rigorous evidence demonstrating that the isolated ‘EVs’ are sEVs. This includes size distribution, zeta potential, electron microscopy, and immunoblotting of classical EV markers, mitochondrial markers (confirming the presence of mitochondria in asthmatic EVs), and cellular (Golgi) markers (excluding cell debris contamination). The authors used a mito-GFP marker to determine mitochondria-containing sEVs, which needs additional electron microscopy images to show the presence and morphology of mitochondria in sEVs.

Response: We appreciate these suggestions from the reviewer. We have previously characterized human BALF EVs and human MDRC-derived EVs previously by NanoSight and ImageStream flow cytometry and reported our findings in PMID: 29986209 and PMID: 29985427. Now, we have access to two cutting-edge platforms and have now performed more precise and extensive characterization using these platforms. We have used Spectradyne nCS1 particle analyzer that delivers accurate particle size and concentration and these data are included in the Supplementary Figures S1 and S15. Additionally, we have used Oxford Nanoimaging platform in conjunction with the EV profiler kit to characterize BALF and MDRC derived EVs from humans and mice. These data are now presented in Supplementary Figures S2 and S16. In our nanoimaging studies, we have evaluated Tomm20 expression in the human sEVs (Supplementary Figure S2). Additionally, we performed western blot analyses (Supplementary Figure S2) of BALF sEVs which shows the presence of CD81 and TIM23. We have performed limited CryoEM studies with MDRC-derived sEVs isolated from Mito-QC mice with asthma compared to sensitized controls. The EM details of the morphology of these MDRC-derived vesicles are aligned what we have reported before (PMID: 29986209). In the Cryo-EM images provided in Supplementary Figure S16, we show double membrane EVs with diameters consistent with sEVs and containing electron dense inclusions. Confirmation of these electron components would require significant extended efforts by Immunogold approaches and resources that are beyond the scope of this current study.

Comment 2: ImageStream that combines flow cytometry with fluorescence imaging technology may be used to analyze large EVs but not small EVs precisely. The author could determine this by comparing the events/volume detected by ImageStream and the nanoparticle number measured by Nanosight. Also, please present the average size and smallest size of detected EVs by ImageStream.

Response: We have performed additional characterization as mentioned above by Spectradyne nCS1 particle analyzer, Oxford Nanoimaging platform and western blot analyses. These data are provided in Supplementary Figures S1, S2, S15 and S16.

Comment 3: The authors chose a very low dose of EVs for the treatments, both in vitro and in vivo, compared with most current EV studies. A ratio of 1:10 (T cells:sEVs) was used to treat T cells, and 1x10e8 was used to treat mice. Could the authors provide a rationale for choosing these doses? Also, are there dose-dependent effects of these EVs in vitro and in vivo?

Response: In our previous in vitro studies (published) with macrophages and T cells, we established functional responses of sEVs following internalization at a 1:10 ratio. During those studies, we performed dose studies and found that there was cytotoxicity associated with higher doses of sEVs *in vitro*. We have also evaluated in vivo transport of EVs using different doses and

found that transfer of 10^8 EVs resulted in in vivo internalization by cell populations in the lung. We have therefore used these doses to evaluate in vivo modulation of T cell polarization. While some of these studies were published in the context of tumors, we have not reported this in the in vivo models of asthma.

Comment 4: The authors showed the presence of dysfunctional mitochondria in MDRC-derived EVs from asthmatic patients. Is there any mitochondrial dysfunction in MDRCs among healthy and asthmatic patients?

In both human and mouse MDRCs, increased DRP1 oligomer and monomer ratio was noted in the asthmatics compared to controls (See Figure 5 and Figure 9) indicative of altered mitochondrial dynamics in these MDRCs. We have published previously that ROS production is enhanced by these MDRCs (PMIDs 25420684 & 21471960) and their effects on human and mouse T cell proliferative responses.

Comment 5: The authors showed that blockage of MHC-II inhibited the EV-mediated T cell activation without affecting their internalization. However, they also showed that EV-mediated T-cell activation is dependent on ROS produced by dysfunctional mitochondria upon internalization. Would blockage of MHC-II inhibit/prevent the release/generation of ROS from these EV-transported mitochondria? Please discuss the potential mechanism.

Response: We have not assessed this mechanism. But we did investigate ROS-mediated NF-kB signaling and RELA expression that may contribute to Th responses. These data are now provided in Supplementary Figure S10.

Comment 6: The authors used two methods for isolating EVs. Ultracentrifugation was used to isolate sEVs from BALF, and the Total Exosome Isolation kit was used to isolate sEVs from the MDRC conditional medium. Please explain the reasons.

Response: As large volumes of human BALF are utilized to isolate sEVs, we have utilized ultracentrifugation. We have extensively characterized EVs isolated from both BALF and MDRCs and show that they have characteristic markers, size and morphology of sEVs. These additional data are provided in the Supplementary Figures S1, S2, and S16.

Comment 7: Some typos to be corrected. Line 172, “CD81” should be “CD81+”. Line306, it should be “with unchanged” or “without changed”. Line 677, “E” should be “G”

Response: We regret this oversight and have made these corrections in the revised manuscript.

Comment 8: Figure 2J should be placed before Figure 2F-I.

Response: We appreciate this comment and have made this change in the Figure 2.

Reviewer #3:

Hough and colleagues report in this manuscript that myeloid-derived regulatory cells (MDRCs) can secrete small extracellular vesicles (sEVs) that control CD4+ T cell responses in asthma.

They found that BALF sEVs from asthmatic patients promote CD4+ T cell proliferation and Th2/Th17 immune responses. Interestingly, MDRC-derived sEVs contain similar amounts of mitochondria (Mito) in both healthy controls and asthmatic patients, but sEVs from asthmatics exhibited elevated levels of mitochondrial membrane potential. These sEVs can enter CD4+ T cells through membrane fusion at the site of cytoskeletal polarization and mitochondrial trafficking. This fusion is mediated by dynamin and requires sEV-derived MHC-II, mitochondrial complex III/V activity, and reactive oxygen species (ROS) to activate CD4+ T cells. Additionally, DRP1 appears to regulate mitochondrial packaging in Mito-sEVs. Intranasal transfer of Mito-sEVs also exacerbates the asthmatic immune response. Overall, while this work presents some interesting findings, it is very difficult to read due to a lack of clear logical flow and poor organization. The manuscript would benefit from restructuring and consolidating the data presentation to improve readability. The major concerns are technical concerns regarding the imaging and flow cytometry, which will need to be carefully addressed, before solid conclusions can be drawn from these experiments.

Response: We are grateful to the reviewer for these comments and provide a point-by-point response below.

Comment 1: Technical Concerns with Imaging: The ability of imaging to determine co-expression of multiple markers, particularly cytokine expression, is a major technical concern. Consider verifying "co-expression" at the population level using flow cytometry.

Response: We appreciate this comment of the reviewer. Fig 7A-F in the previous submission were flow cytometry plots. We recognize that we did not provide representative flow cytometry plots for Th cell polarization or the flow gating strategies and controls for flow cytometry in the previous submission. These are now presented in Figures 1, 7, Supplementary Figures S3, S4, S12 and S13. Flow cytometry was used always to determine Th polarization at the population level. The imageStream image strips were included to show co-expression of Th cytokines and sEV markers at the cellular level. These have now been removed.

Comment 2: Flow Cytometry Data Quality: The flow cytometry data lacks proper control groups, appears to be improperly compensated, and may have incorrect gating. These issues need to be addressed.

Response: We have provided flow cytometry plots and gating strategies for our flow cytometry data as mentioned in the response to Comment 1. These data are now presented in Main Figures 1 and 7 and Supplementary Figures S3, S4, S12 and S13. Additional flow cytometry gating strategies are provided in Supplementary Figures S19, S20 and S21.

Comment 3. Logical Flow: The manuscript needs a better logical flow. Summarize the data more effectively, rather than randomly assigning them slots in the main figures and repeatedly presenting similar data in different formats.
Response: We have made efforts to summarize data effectively and carefully.

Comment 4. Figure Presentation: Many figures of the same grouping are presented in different formats, making them busy and difficult to follow. For example, Fig. 1A, C, D, E, and F could

follow the same format. Sub-figures are too close to each other, making it difficult to locate legends. Figures addressing similar questions and using the same experimental strategy should be grouped together.

Response: We have reformatted the graphs to follow the same format. We have also adjusted the spacing between the figures to make the legends more visible. Some of our co-culture experimental groups are extensive and these pose difficulties in restructuring the entire paper.

Comment 5. Figure Legends: Figure legends do not need to repeat the methods in great detail, such as the dose of IL-2 in stimulation and the flow cytometry machine model.

Response: As the figures and supplementary figures had to stand alone with figure legends for each figure, we have included all the details for each figure.

Comment 6. Endo-RFP+ Cells: The Endo-RFP+ cells are presented throughout the main figures, but it was determined that the endosome pathway is minimally involved in CD4+ activation by sEVs. It is suggested that the key figure on this finding be retained in the main text, while the rest of the Endo-RFP data should be moved to the supplementary section to improve readability.

Response: We have maintained the RFP figures throughout as these comparisons are needed for sEV fusion, internalization, activation and polarization.

Comment 7. Figure 2: Panels E and J are representative figures of labeling, while panels C, D, F-I quantify the effects of sEVs from control and asthmatic BALF. Panels E and J can be moved to the supplementary section. Panels C, D, F-I should have consistent formats.

Response: We have restructured Figure 2 to have consistent format for the graphs.

Comment 8. Figure 3: This figure is confusing. The functions of Mito-GFP and Endo-RFP should be described (e.g., "to label XX with XX, and XX with XX"). The pie chart in panel 3B has confusing colors that should be annotated, and the colored legends should intersect with panel 3C. Panel 3D can be moved to the supplementary section. Additionally, why is panel 3B needed? It is an essential part of panel 3C, but presented differently. Not every experiment needs to be included—consider consolidating the data and presenting a comprehensive summary.

Response: We agree this is a complex figure and have made the best effort to show the data in the most concise manner.

Comment 9. Figure 4: Similar issues are present as in Figure 3.
Response: We agree this is a complex figure and have made the best effort to show the data in the most concise manner.

Comment 10. Combining Figures: Figures 3, 4, and 5A-B could be combined, as they share the same schematics with different inhibitors. All of these address the required sEV factors in CD4+ activation.

Response: These figures address the role of different mitochondrial complex proteins. Hence we have maintained these figures separate.

Comment 11. Figure 5E (right): Controls should include multiple replicates from healthy and asthmatic samples. The validation of knockdown (KD) should be presented before the functional data. Panel 5E (left) is a summary of panel 5F.

Response: We appreciate this comment. We have performed siRNA knockdown in multiple samples (n=3 healthy and n=4 asthma subjects). These data are presented in Figure 5.

Comment 12. Mitochondrial Fission Section: This section could be a standalone figure and should precede Figures 3, 4, and 5A-B, as it describes how Mito-sEVs are packaged.

Response: Figure 5 summarizes all data regarding mitochondrial function in the sEVs that is contributed by alteration in mitochondrial dynamics in the MDRCs.

Comment 13. Figure 6: This figure can be merged with Figure 2, as both analyze how MDRC-derived sEVs enter CD4+ T cells using the same experimental strategy.

Response: We have restructured several figures and added supplementary figures in support of our observations.

Comment 14. Figure 7: Move panel 7G, which verifies imaging techniques, to the supplementary section, and move panels 7H-J, which analyze allogenic effects, to the supplementary section as well. Panels K-P should test Mito-GFP+ sEVs and should not claim their mechanism as DAMP-mediated. Instead, this data suggests that sEVs can promote both innate and adaptive inflammatory responses associated with asthma.

Response: We have moved these data on allogeneic effects and sEV-mediated innate responses to the supplementary figures. The imaging data have been removed.

15. Figures 8 and 10: These figures need experimental schematics and an OVA-sensitized + non-sEV control group. The gating strategies shown in Figures S10 and S11 are concerning, suggesting that the flow data were not properly compensated, and most of the markers were gated on backgrounds rather than actual signals. Please note that cells from inflamed airways tend to be larger and exhibit higher background signals in flow cytometry. The gating strategy should include FSC, SSC, singlets, viability, and markers. The gating strategy for the myeloid cells should be included as well.

Response: We have provided gating strategies in Supplementary Figures 19, 20 and 21.

Comment 16. Th2/Th17 Response in OVA Model: The OVA model is Th2-biased. The high response of Th17 may be an artifact of the flow gating. To verify this, analyze BALF for Th2 and Th17 cytokines by ELISA.

Response: We appreciate this comment. We have included BALF cytokine data in Figure 10.

Comment 17. Lung Histology: The lung histology of the asthmatic mouse model should be presented to reveal the effects of sEVs in asthma.

Response: MitoQC mice were obtained on an MTA with investigators from Australia. This required us for these mice to be bred, genotyped and provided by investigators on the MTA done with non-NIH and non-foundation grant funds. Our sensitization and challenge experiments had to be performed on only institutional funds. We regret that this limited us from maintaining a large colony with enough animals ourselves or obtaining enough numbers of animals to perform cell sorting from sensitized and challenged animals as well as flexivent studies and additional extensive histologic evaluations. We have provided IgE measure

Reviewer#4

In this study, Hough et al. found that airway Myeloid-derived regulatory cells (MDRCs) from asthmatics can transfer mitochondria through Small extracellular vesicles (sEVs) via membrane fusion. This is associated with T cell activation and Th17 and Th2, but not Th1 cell differentiation. sEV-derived mitochondria co-localized with immune synapse and their fission and function affected the functions of recipient T cells and related responses. These findings could be of interest. Addressing the following comments may help to improve the current study.

Response: We are grateful to the reviewer for these comments and provide a point-by-point response below.

Comment 1: It is important to reveal what are the critical signaling pathways activated by sEV with or without rotenone to lead to T cell activation, especially only for Th2, Th17 but not for Th1 cells. In addition, it is important to address if sEV-mediated effects are different in naïve vs effector vs Treg cells.

Response: We appreciate this comment from the reviewer. We performed experiments to investigate the effects of rotenone treated sEVs on T cells; specifically we wanted to address if it is through rotenone's effects on reverse electron transport or through its effects on ROS-mediated signaling mediated by sEVs. As shown in Supplementary Figure S10, % of $CD4^+$ T cells that have internalized $MitoTracker^+$ sEVs treated with rotenone showed significant differences in % NF-kB $p65(pS529)^+$ cells in both Healthy and Asthmatic groups compared to untreated controls. The change in NF-kB activation in T cells internalizing rotenone treated sEVs in T cells from healthy individuals were significantly lower than from asthmatics. The % NF-kB $p65(pS529)^+$ T cells from asthmatics that internalized untreated sEVs (DMSO controls for rotenone treatment) was increased, but not significantly different from similar controls from healthy subjects. Consistent with this, RELA expression was significantly higher in the +rotenone sEV + T cell group from asthmatics compared to healthy T cells and other relevant controls.

Comment 2: Direct evidence of increased mitochondrial fission in asthmatics sEVs should be provided. In addition, it should be addressed if Drp1 mutants that cannot oligomerize affect mitochondrial fission in sEVs and the function of sEVs in T cells.

Response: Our Cryo-EM studies reveal the presence of electron dense inclusions. Our data suggest that proinflammatory ROS-producing MDRCs (humans and mice) have increased mitochondrial fission and Drp1 oligomerization that allows packaging of these mitochondria that have undergone fission to be packaged within sEVs and these can modulate T cell responses.

Our data suggest that DNM1L KO in T cells reduces the packaging in sEVs. We are not proposing that mitochondrial fission occurs within the sEVs. We have no evidence at this point to support this.

Comment 3: Direct evidence of sEVs affecting the organization of the synapse through cytoskeletal and mitochondrial reorganization and related downstream signaling should be provided. In addition, the relationship between mitochondrial function (ATP, ROS, fission) in the sEVs and synapse formation and downstream signaling in T cells needs further investigation and elaboration.

[TABLE REDACTED]

Response: We have only very preliminary evidence from a global kinomic analyses in T of both groups cells treated with autologous sEVs that PKC family is increased in sEV-treated T cells from asthmatics (see Table). Class II MHC blocking may modulate phosphorylation of PKC family members at the polarized cytoskeleton of activated T cells. But providing direct evidence affecting synapse organization would require a series of in vitro and in vivo experiments that are beyond the scope of this study. The only supportive data we have is co-localization of sEVs containing mitochondria in the polarized cytoskeleton of recipient activated T cells and TCR downstream signaling we see in the T cells. Additionally, blocking antibody studies and membrane fusion events suggest that there are membrane signaling occurring at the site of sEV-T cell fusion. Therefore, we cannot provide direct evidence of the synapse, and hence, have revised our discussion of potential role for sEVs in the organization of the synapse.

Comment 4: For mouse study, it is unclear if the observations were due to a direct sEV-mediated effect on CD4 T cells or also due to an indirect, sEV-mediated effect on other immune or non-immune cells. Can sEV containing/conditioned CD4 T cells promote disease in mice on their own?

Response: Transfer of MDRC-sEVs isolated from donor mice with asthma into sensitized recipients did not exacerbate airway inflammation, IgE levels, Muc5Ac or enhance Th2 type responses in recipient mice. Our data suggest that non-immune $CD45^{neg}$ cells from human BALF internalizing MDRC-sEVs did not activate T cells. These data are provided in Supplementary Figure S9. We have not transferred sEV conditioned CD4 T cells to see if it will promote disease.

Comment 5: Fig. 1A-1B, FACS plots of CFSE dilution should be shown. The % of proliferating cells are small. Another method, e.g. BrdU incorporation assays, should also be used to evaluate T cell proliferation.

Response: Thank you for this comment. We have provided representative FACS plots of CFSE dilution in both peripheral and airway T cell-co-cultures with sEVs. These are provided in the Supplementary Figure S3 of the revised manuscript.

Comment 6: Fig. 1C, 1D and S1A, why less than 2% activated T cells expressed CD69 and pZap70 (Fig. 1D), while more than 40% T cell proliferated? A co-identification of CFSE, CD69, CD154, CD25 should be done to correlate TCR activation and T cell proliferation.

Response: We agree that we are not seeing as robust an increase in TCR activation as the increase in T cell proliferation. We would like to point out that TCR activation shown with pZap70 in Figure 1 is in the peripheral CD4 T cells and not airway T cells where we saw a robust change in proliferation. Unfortunately, isolating sufficient airway T cells for all assays utilized was not feasible. We are in the process of evaluating if we can assess this adequately in T cells from explanted lungs and lymph nodes.

Comment 7: The difference between + vs. – sEVs of IL4 and IL17 protein production was about 2 folds per flow-cytometry (Fig. 1E and Fig. S1B), yet the mRNA expression was about 10 folds. What accounts for such a big difference?

Response: We provide cytokine data for IL-4 and IL-17 in support of our in vitro human data and in vivo mouse data. We agree with the reviewer that mRNA changes are more robust in those samples. There is variability in extent of T cell proliferation and Th polarization elicited by sEVs in autologous T cells from asthmatics. This may account for these differences.

Comment 8: Fig. 1G, GATA3 was downregulated in + sEVs samples, why Th2 was upregulated in these samples? Were Th17 specific gene signatures, including Ror, preferentially upregulated in + sEVs samples?

Response: We appreciate this comment and have provided additional data for RORC and GATA3 expression in Figure 1. RORC expression significantly changed with sEV-co-culture with T cells in asthmatics. We saw an increase in expression for GATA3, but didnt reach significance. Similar sample variability was seen in our NanoString transcriptomic data for GATA-3. But our Th2 cytokine data are supportive of Th2 differentiation.

Comment 9: Fig. S3A. T cell proliferation assays as in Fig. 1D should also be performed to assess T cell activation, as the readout could be more robust.

Response: We agree that readouts could be more robust in proliferation assays. These robust responses are more often seen with airway T cells, for which high enough purified cell numbers are not easy to obtain from BALF.

Response to Reviewers:

Reviewer #1

This is a well-designed and executed study with a defined goal of establishing the mechanism by which sEVs play a role in immune regulation of asthma. The authors focus on fragmented mitochondria in the sEVs as mediators of immune cell proliferation and differentiation. Authors use human patient-derived T cells for in vitro studies and corroborate their findings using a murine model of asthma. The study findings are valuable in advancing our understanding of immunopathogenesis of asthma. Following are a few comments for the authors to consider.

Response: We are grateful to the reviewer for these comments, acknowledging the translational approach of our study. We have performed additional experiments and modified the manuscript accordingly, and provide a point-by-point response below.

Comment 1: Figure 1A-D: sEVs are not well characterized to confirm the expression of Class II MHC.

Response: We appreciate this comment and now provide data in Supplementary Figure 1 E, F, G and J, clearly showing Class II MHC expression in sEVs in both healthy individuals and asthmatics, on single EVs measured by Image Stream. Previously, we have also characterized BALF EVs and reported on Class II expression in PMID: 29986209.

Comment 2: Fig 1E data are not very convincing. Fig 1F data needs to be validated using flow cytometry or western blotting.

Response: We recognize that we did not provide adequate supporting data previously. We now provide flow cytometry plots (contour with outliers) and gating strategies and with additional representative plots for all Th polarization studies. These are now presented in Main Figures 1 and 8 as well as Supplementary Figures 3, 4, 10, 16, 17 and 18. Additionally, we now provide cytokine measurements in supernatants from our in vitro studies (Figure 1G) and in BALF from our in vivo studies (Figure 11 H, I), which support our previous conclusions that asthma sEVs induce more T cell activation.

Comment 3: For transcriptomics data, the authors have used only Th17 differentiation and not Th2 differentiation or CD4+ T cell proliferation phenotype augmented by sEVs.

Response: We appreciate this comment and have provided additional data for RORC and GATA3 expression in Figure 1. RORC expression significantly changed with sEV-co-culture with T cells in asthmatics (Fig. 1I, left plot). We saw an increase in expression for GATA3 (Fig. 1I, right plot), but this did not reach significance. Similar human sample to sample variability was seen in our transcriptomic data for GATA-3, but our Th2 cytokine data (Fig. 1G) are clearly supportive of Th2 differentiation.

Comment 4: Fig 4 and 5 rely (few other datasets) entirely on the expression of markers after treatment with different inhibitors. These data could be strengthened by measuring cytokine release or some other functional readouts.

Response: We appreciate this comment from the reviewer. In the studies with rotenone, we performed experiments to further evaluate the role of NF-kB signaling in the effect we see with rotenone and have quantitated RELA expression as RELA and NF-κB signaling are both involved in Th differentiation. These results are now shown in Supplementary Figure S10. Additionally, we looked at potential outcomes of rotenone-induced NF-κB signaling on Th differentiation and show enhancement of Th2 and Th17 differentiation in T cells from asthmatics. These results are now presented in Supplementary Figure S18.

Comment 5: Drp1-mediated and mitochondrial fission responsible for mitochondrial packaging in sEV is explored in this study. However, the data seem to suggest that Drp1-fission-mediated packaging is only a small portion of mitochondrial packaging as the packaging is not eliminated in knockdown cells.

Response: We agree that, although significant reduction in sEV packaging is achieved, it is not completely eliminated in knockdown cells. We have performed additional experiments to use a peptide inhibitor of Drp1 oligomerization and activity. This inhibition was performed in MDRCs sorted from in vivo studies with allergic Mito-QC mice as well as C57BL/6 mice. In these studies, again we see significant reduction in sEV-mitochondria but not complete inhibition. However, we argue that the mechanism is likely of biological importance as we see a significant effect, even though additional unexplored mechanisms may be involved. Furthermore, we are not achieving complete knockdown with siRNA pool in multiple samples; therefore, the elimination may be enhanced if this was achieved. The data from these studies on mitochondrial dynamics has been now assembled as a separate Figure 7.

Comment 6: Mitochondrial DAMP signaling involved in the immune activation muddles the key message of the manuscript. How rest of the data showing mitochondrial transfer into cells directly activate T cells reconcile with DAMP?

Response: We agree with the reviewer that based on our current data, we can only claim that sEVs promote both innate and adaptive immune responses and we have shortened this part (lines 363-374).

Comment 7: In vivo studies using OVA mice lack complete characterization of asthma phenotype. The authors primarily show the effect on airway inflammation.

Response: We now have performed histological evaluation of lung tissues from our in vivo studies and show increased inflammatory cell infiltration, goblet cell hyperplasia, collagen accumulation and mucin hypersecretion (Figure 10). We have provided OVA IgE measurements in BALF and serum and readouts of Muc5AC in BALF and measures of inflammatory cell infiltration (Supplementary Figure S19) MitoQC mice were obtained on an MTA with investigators from Australia and had to be maintained on non –NIH funds in agreement with MTA. This allowed us to perform only limited studies. We agree that asthma is not confirmed in the mice with additional flexivent studies and we have focused on airway inflammation and other important signatures of allergic airway inflammation in the mouse studies.

Reviewer #2:

In this manuscript, Hough et.al. identified a pathological role of airway MDRC-derived sEVs in asthmatics with extensive ImageStream data. The authors demonstrated that only asthmatic MDRC-derived sEVs contain dysfunctional mitochondria (may also called mitovesicles), which are indispensable for triggering T cell activation and Th cell polarization. They also explored the mechanisms underlying the secretion and uptake of these mitovesicles. They showed that the secretion of mitovesicles is drp1-dependent, and the uptake is mediated by membrane fusion. Importantly, they modeled the airway inflammation in animal models by intranasal administration of these mitovesicles. In general, this study is of great interest and significance. Several comments need to be addressed.

Response: We are grateful to the reviewer for these comments. We have performed additional experiments and modified the manuscript according to the suggestions, and provide a point-by-point response below.

Comment: 1. Lack of basic EV characterization. The authors used a Total Exosome Isolation kit to isolate EVs, which usually co-precipitate a lot of other extracellular compartments, including small cell debris, large EVs, ribonucleoproteins, etc. The authors need to provide rigorous evidence demonstrating that the isolated 'EVs' are sEVs. This includes size distribution, zeta potential, electron microscopy, and immunoblotting of classical EV markers, mitochondrial markers (confirming the presence of mitochondria in asthmatic EVs), and cellular (Golgi) markers (excluding cell debris contamination). The authors used a mito-GFP marker to determine mitochondria-containing sEVs, which needs additional electron microscopy images to show the presence and morphology of mitochondria in sEVs.

Response: We appreciate these suggestions from the reviewer. Now, we have access to two new cutting-edge platforms and have now performed more precise and extensive characterization using these platforms. We have used Spectradyne nCS1 particle analyzer that delivers accurate particle size and concentration and these data are included in the Supplementary Figures S1 and S12. Additionally, we now also included the Oxford Nanoimaging platform in conjunction with the EV profiler kit to characterize BALF and MDRC derived EVs from humans and mice. These data are now presented in Supplementary Figures S2 and S13. In our nanoimaging studies, we have evaluated Tomm20 expression in the human sEVs (Supplementary Figure S2). Additionally, we performed western blot analyses of BALF sEVs (Supplementary Figure S2) which show the presence of CD81 and TIM23. We have previously characterized human BALF EVs and human MDRC-derived EVs by Cryo-EM, NanoSight and ImageStream flow cytometry and reported our findings in PMID: 29986209 and PMID: 29985427. We have now performed limited CryoEM studies with MDRC-derived sEVs isolated from Mito-QC mice with asthma compared to sensitized controls. The EM details of the morphology of these MDRC-derived vesicles are aligned with our earlier reports (PMID: 29986209). In the Cryo-EM images provided in Supplementary Figure S13, we show double membrane EVs with diameters consistent with sEVs and containing electron dense inclusions. Confirmation of these electron components would require significant extended efforts by Immunogold approaches and resources that are beyond the scope of this current study.

Comment 2: ImageStream that combines flow cytometry with fluorescence imaging technology may be used to analyze large EVs but not small EVs precisely. The author could determine this by comparing the events/volume detected by ImageStream and the nanoparticle number

measured by Nanosight. Also, please present the average size and smallest size of detected EVs by ImageStream.

Response: We have performed additional characterization as mentioned above by Spectradyne nCS1 particle analyzer, Oxford Nanoimaging platform and western blot analyses. These data are provided in Supplementary Figures S1, S2, S12 and S13, and described on lines 118-134 and 311-323.

Comment 3: The authors chose a very low dose of EVs for the treatments, both in vitro and in vivo, compared with most current EV studies. A ratio of 1:10 (T cells:sEVs) was used to treat T cells, and 1x10e8 was used to treat mice. Could the authors provide a rationale for choosing these doses? Also, are there dose-dependent effects of these EVs in vitro and in vivo?

Response: In our previous in vitro studies (published) with macrophages and T cells, we established functional responses of sEVs following internalization at a 1:10 ratio. During those studies, we performed dose studies and found that there was cytotoxicity associated with higher doses of sEVs *in vitro*. We have also evaluated in vivo transport of EVs using different doses and found that transfer of 10^8 EVs resulted in in vivo internalization by cell populations in the lung. We have therefore used these doses to evaluate in vivo modulation of T cell polarization. While some of these studies were published in the context of tumors, we have not reported this in the in vivo models of asthma.

Comment 4: The authors showed the presence of dysfunctional mitochondria in MDRC-derived EVs from asthmatic patients. Is there any mitochondrial dysfunction in MDRCs among healthy and asthmatic patients?

Response: Yes, there is, in asthmatics. In both human and mouse MDRCs, increased DRP1 oligomer and monomer ratio was noted in the asthmatics compared to controls (See Figure 7) indicative of altered mitochondrial dynamics in these MDRCs. We have published previously that ROS production is enhanced by these MDRCs (PMIDs 25420684 & 21471960) and their effects on human and mouse T cell proliferative responses.

Comment 5: The authors showed that blockage of MHC-II inhibited the EV-mediated T cell activation without affecting their internalization. However, they also showed that EV-mediated T-cell activation is dependent on ROS produced by dysfunctional mitochondria upon internalization. Would blockage of MHC-II inhibit/prevent the release/generation of ROS from these EV-transported mitochondria? Please discuss the potential mechanism.

Response: We have not exactly assessed this particular mechanism. However, we did investigate ROS-mediated NF-kB signaling and RELA expression that may contribute to T cell activation and Th polarization. These data are now provided in Supplementary Figures S10 and S18.

Comment 6: The authors used two methods for isolating EVs. Ultracentrifugation was used to isolate sEVs from BALF, and the Total Exosome Isolation kit was used to isolate sEVs from the MDRC conditional medium. Please explain the reasons.

Response: As large volumes of human BALF are utilized to isolate sEVs, we have utilized ultracentrifugation. We have extensively characterized EVs isolated from both BALF and MDRCs and show that they have characteristic markers, size and morphology of sEVs. These additional data are provided in the Supplementary Figures S1, S2, S12 & S13.

Comment 7: Some typos to be corrected. Line 172, “CD81” should be “CD81+”. Line306, it should be “with unchanged” or “without changed”. Line 677, “E” should be “G”

Response: We regret this oversight and have made these corrections in the revised manuscript.

Comment 8: Figure 2J should be placed before Figure 2F-I.

Response: We appreciate this comment and have made this change in the Figure 2.

Reviewer #3:

Hough and colleagues report in this manuscript that myeloid-derived regulatory cells (MDRCs) can secrete small extracellular vesicles (sEVs) that control CD4+ T cell responses in asthma. They found that BALF sEVs from asthmatic patients promote CD4+ T cell proliferation and Th2/Th17 immune responses. Interestingly, MDRC-derived sEVs contain similar amounts of mitochondria (Mito) in both healthy controls and asthmatic patients, but sEVs from asthmatics exhibited elevated levels of mitochondrial membrane potential. These sEVs can enter CD4+ T cells through membrane fusion at the site of cytoskeletal polarization and mitochondrial trafficking. This fusion is mediated by dynamin and requires sEV-derived MHC-II, mitochondrial complex III/V activity, and reactive oxygen species (ROS) to activate CD4+ T cells. Additionally, DRP1 appears to regulate mitochondrial packaging in Mito-sEVs. Intranasal transfer of Mito-sEVs also exacerbates the asthmatic immune response. Overall, while this work presents some interesting findings, it is very difficult to read due to a lack of clear logical flow and poor organization. The manuscript would benefit from restructuring and consolidating the data presentation to improve readability. The major concerns are technical concerns regarding the imaging and flow cytometry, which will need to be carefully addressed, before solid conclusions can be drawn from these experiments.

Response: We are grateful to the reviewer for the positive comments. Regarding the flow of the manuscript, we have tried to clarify certain parts, including some elaboration on why a certain experiment is performed. For the rest of the comments, we provide a point-by-point response below.

Comment 1: Technical Concerns with Imaging: The ability of imaging to determine co-expression of multiple markers, particularly cytokine expression, is a major technical concern. *Consider* verifying "co-expression" at the population level using flow cytometry.

Response: We appreciate this comment of the reviewer. In the revised manuscript Figure 8 (Fig 7A-F) in the previous submission were quantitation from flow cytometry analyses. We recognize that we did not provide representative flow cytometry plots for Th cell polarization or the flow gating strategies and controls for flow cytometry in the previous submission. These are now presented in Figures 1, 8, Supplementary Figures S3, S4, S16 and S17 and S18. Flow

cytometry was used always to determine Th polarization at the population level. The imageStream image strips were included to show co-expression of Th cytokines and sEV markers at the cellular level. These have now been removed as we acknowledge reviewer's point that cytokine expression with multiple markers cannot be accurately represented in imaging studies.

Comment 2: Flow Cytometry Data Quality: The flow cytometry data lacks proper control groups, appears to be improperly compensated, and may have incorrect gating. These issues need to be addressed.

Response: We have provided flow cytometry plots and gating strategies for our flow cytometry data as requested. These data are now presented in Main Figures 1 and 8 and Supplementary Figures S3, S4, S10, S16, S17, S18, S21, S22 & S23.

Comment 3. Logical Flow: The manuscript needs a better logical flow. Summarize the data more effectively, rather than randomly assigning them slots in the main figures and repeatedly presenting similar data in different formats.

Response: We have made efforts to summarize data effectively and carefully, and we also added a few explanatory sentences to explain the purpose of certain experiments which we hope has helped the flow of the paper.

Comment 4. Figure Presentation: Many figures of the same grouping are presented in different formats, making them busy and difficult to follow. For example, Fig. 1A, C, D, E, and F could follow the same format. Sub-figures are too close to each other, making it difficult to locate legends. Figures addressing similar questions and using the same experimental strategy should be grouped together.

Response: We have reformatted the graphs to follow the same format. We have also adjusted the spacing between the figures to make the legends more visible. Some of our co-culture experimental groups are extensive and these pose difficulties in restructuring the entire paper.

Comment 5. Figure Legends: Figure legends do not need to repeat the methods in great detail, such as the dose of IL-2 in stimulation and the flow cytometry machine model.

Response: As the figures and supplementary figures had to stand alone with figure legends for each figure according to instruction to authors, we have included all the details for each figure.

Comment 6. Endo-RFP+ Cells: The Endo-RFP+ cells are presented throughout the main figures, but it was determined that the endosome pathway is minimally involved in CD4+ activation by sEVs. It is suggested that the key figure on this finding be retained in the main text, while the rest of the Endo-RFP data should be moved to the supplementary section to improve readability.

Response: We have maintained the RFP figures throughout as these comparisons are needed for sEV fusion, internalization, activation and polarization. It is also unknown whether inhibition of mitochondrial complex proteins would change their co-localization within the cells.

Comment 7. Figure 2: Panels E and J are representative figures of labeling, while panels C, D, F-I quantify the effects of sEVs from control and asthmatic BALF. Panels E and J can be moved to the supplementary section. Panels C, D, F-I should have consistent formats.

Response: We have restructured Figure 2 to have consistent format for the graphs.

Comment 8. Figure 3: This figure is confusing. The functions of Mito-GFP and Endo-RFP should be described (e.g., "to label XX with XX, and XX with XX"). The pie chart in panel 3B has confusing colors that should be annotated, and the colored legends should intersect with panel 3C. Panel 3D can be moved to the supplementary section. Additionally, why is panel 3B needed? It is an essential part of panel 3C, but presented differently. Not every experiment needs to be included—consider consolidating the data and presenting a comprehensive summary.

Response: We agree this is a complex figure and have made the best effort to show the data in the most concise manner. We have removed 3B and 4B as it is representing the same data as in the graphs.

Comment 9. Figure 4: Similar issues are present as in Figure 3.

Response: Both fig 3B and 4B have been removed.

Comment 10. Combining Figures: Figures 3, 4, and 5A-B could be combined, as they share the same schematics with different inhibitors. All of these address the required sEV factors in CD4+ activation.

Response: These figures address the role of different mitochondrial complex proteins. Hence we have maintained these figures separate. But we hope that the removal of the B figures in both will simplify the interpretation of the data.

Comment 11. Figure 5E (right): Controls should include multiple replicates from healthy and asthmatic samples. The validation of knockdown (KD) should be presented before the functional data. Panel 5E (left) is a summary of panel 5F.

Response: We appreciate this comment. We have performed siRNA knockdown in multiple samples (n=3 healthy and n=4 asthma subjects). These data are now presented in Figure 7.

Comment 12. Mitochondrial Fission Section: This section could be a standalone figure and should precede Figures 3, 4, and 5A-B, as it describes how Mito-sEVs are packaged.

Response: Figure 7 now summarizes all data regarding mitochondrial function in the sEVs that is contributed by alteration in mitochondrial dynamics and Drp1 in the MDRCs, so we would prefer to keep it in this order.

Comment 13. Figure 6: This figure can be merged with Figure 2, as both analyze how MDRC-derived sEVs enter CD4+ T cells using the same experimental strategy.

Response: We have restructured several figures and added supplementary figures in support of our observations.

Comment 14. Figure 7: Move panel 7G, which verifies imaging techniques, to the supplementary section, and move panels 7H-J, which analyze allogenic effects, to the supplementary section as well. Panels K-P should test Mito-GFP+ sEVs and should not claim their mechanism as DAMP-mediated. Instead, this data suggests that sEVs can promote both innate and adaptive inflammatory responses associated with asthma.

Response: We appreciate this comment from the reviewer. We have now revised our interpretations and do not claim that the mechanisms is DAMP-mediated. As requested, we now describe that sEVs can promote both innate and adaptive inflammatory responses associated with asthma (Lines 363-374). We have furthermore moved these data on allogeneic effects and sEV-mediated innate responses to the supplementary figure S15.

15. Figures 8 and 10: These figures need experimental schematics and an OVA-sensitized + non-sEV control group. The gating strategies shown in Figures S10 and S11 are concerning, suggesting that the flow data were not properly compensated, and most of the markers were gated on backgrounds rather than actual signals. Please note that cells from inflamed airways tend to be larger and exhibit higher background signals in flow cytometry. The gating strategy should include FSC, SSC, singlets, viability, and markers. The gating strategy for the myeloid cells should be included as well.

Response: We thank the reviewer for this suggestion. An experimental schematic is now included as Supplementary Figure 14. We have provided gating strategies in revised Supplementary Figures 21, 22 and 23.

Comment 16. Th2/Th17 Response in OVA Model: The OVA model is Th2-biased. The high response of Th17 may be an artifact of the flow gating. To verify this, analyze BALF for Th2 and Th17 cytokines by ELISA.

Response: We appreciate this comment. We have included BALF cytokine data in Figure 11.

Comment 17. Lung Histology: The lung histology of the asthmatic mouse model should be presented to reveal the effects of sEVs in asthma.

Response: We now provide histoplogy data in Figure 10 which shows that transfer of MDRC-derived sEVs enhance airway inflammation, goblet cell hyperplasia, mucus hypersecretion and collagen accumulation compared to controls.

Reviewer#4

In this study, Hough et al. found that airway Myeloid-derived regulatory cells (MDRCs) from asthmatics can transfer mitochondria through Small extracellular vesicles (sEVs) via membrane fusion. This is associated with T cell activation and Th17 and Th2, but not Th1 cell differentiation. sEV-derived mitochondria co-localized with immune synapse and their fission and function affected the functions of recipient T cells and related responses. These findings could be of interest. Addressing the following comments may help to improve the current study.

Response: We are grateful to the reviewer for these comments and provide a point-by-point response below.

Comment 1: It is important to reveal what are the critical signaling pathways activated by sEV with or without rotenone to lead to T cell activation, especially only for Th2, Th17 but not for Th1 cells. In addition, it is important to address if sEV-mediated effects are different in naïve vs effector vs Treg cells.

Response: We appreciate this comment from the reviewer. We performed experiments to investigate the effects of rotenone treated sEVs on T cells; specifically we wanted to address if it is through rotenone's effects on reverse electron transport or through its effects on ROS-mediated signaling mediated by sEVs. The data are now added in Supplementary Figure S10, % of $CD4^+$ T cells that have internalized $MitoTracker^+$ sEVs treated with rotenone showed significant differences in % NF-κB $p65(pS529)^+$ cells in both Healthy and Asthmatic groups compared to untreated controls. The change in NF-κB activation in T cells internalizing rotenone treated sEVs in T cells from healthy individuals were significantly lower than from asthmatics. The % NF-κB $p65(pS529)^+$ T cells from asthmatics that internalized untreated sEVs (DMSO controls for rotenone treatment) was increased, but not significantly different from similar controls from healthy subjects. Additionally, we investigated if rotenone-induced NF-κB signaling promoted Th polarization and saw increased Th2 and Th17 polarization. These results are now summarized in Supplementary Figure S18. Consistent with this, RELA expression was significantly higher in the +rotenone sEV^+ T cell group from asthmatics compared to healthy T cells and other relevant controls.

Comment 2: Direct evidence of increased mitochondrial fission in asthmatics sEVs should be provided. In addition, it should be addressed if Drp1 mutants that cannot oligomerize affect mitochondrial fission in sEVs and the function of sEVs in T cells.

Response: Our data suggest that proinflammatory ROS-producing MDRCs (humans and mice) have increased Drp1 oligomerization. Drp1 oligomerization is a critical step mitochondrial fission, this would allow packaging of these mitochondria within sEVs and these can modulate T cell responses. Our Cryo-EM studies reveal the presence of electron dense inclusions. We have now used P110, an inhibitor of Drp1 oligomerization and activity and show that P110 treatment of MDRCs reduced packaging of mitochondria in sEVs compared to vehicle treatment. Our data suggest that DNM1L KO in MDRCs also reduces the packaging in sEVs. We are not suggesting that mitochondrial fission occurs within the sEVs. We have no evidence at this point to support this. These data are now presented in Figure 7.

Comment 3: Direct evidence of sEVs affecting the organization of the synapse through cytoskeletal and mitochondrial reorganization and related downstream signaling should be provided. In addition, the relationship between mitochondrial function (ATP, ROS, fission) in the sEVs and synapse formation and downstream signaling in T cells needs further investigation and elaboration.

[TABLE REDACTED]

Response: We have only very preliminary evidence from a global kinomic analyses in T of both groups cells treated with autologous sEVs that PKC family is increased in sEV-treated T cells from asthmatics (see Table). Class II MHC blocking may modulate phosphorylation of PKC family members at the polarized cytoskeleton of activated T cells. But providing direct evidence affecting synapse organization would require a series of in vitro and in vivo experiments that are beyond the scope of this study. The only supportive data we have is co-localization of sEVs containing mitochondria in the polarized cytoskeleton of recipient activated T cells and TCR downstream signaling we see in the T cells. Additionally, blocking antibody studies and membrane fusion events suggest that there are membrane signaling occurring at the site of sEV-T cell fusion. Therefore, we cannot provide direct evidence of the synapse, and hence, have revised our discussion of potential role for sEVs in the organization of the synapse (lines 520-523).

Comment 4: For mouse study, it is unclear if the observations were due to a direct sEV-mediated effect on CD4 T cells or also due to an indirect, sEV-mediated effect on other immune or non-immune cells. Can sEV containing/conditioned CD4 T cells promote disease in mice on their own?

Response: Transfer of MDRC-sEVs isolated from donor mice with asthma into sensitized recipients did not exacerbate airway inflammation, IgE levels, Muc5Ac or enhance Th2 type responses in recipient mice. Further, our histological evaluation is supportive of this lack of exacerbation. Additionally, our data shown in Supplementary Figure S9 suggest that non-immune $CD45^{neg}$ cells from human BALF internalizing MDRC-sEVs did not activate T cells. We have not transferred sEV conditioned CD4 T cells to see if it will promote disease.

Comment 5: Fig. 1A-1B, FACS plots of CFSE dilution should be shown. The % of proliferating cells are small. Another method, e.g. BrdU incorporation assays, should also be used to evaluate T cell proliferation.

Response: Thank you for this comment. We have provided representative FACS plots of CFSE dilution in both peripheral and airway T cell-co-cultures with sEVs. These are provided in the Supplementary Figure S3 of the revised manuscript.

Comment 6: Fig. 1C, 1D and S1A, why less than 2% activated T cells expressed CD69 and pZap70 (Fig. 1D), while more than 40% T cell proliferated? A co-identification of CFSE, CD69, CD154, CD25 should be done to correlate TCR activation and T cell proliferation.

Response: We agree that we are not seeing as robust an increase in TCR activation as the increase in T cell proliferation. We would like to point out that TCR activation shown with pZap70 in Figure 1 is in the peripheral CD4 T cells and not airway T cells where we saw a robust change in proliferation. Unfortunately, isolating sufficient human airway T cells from BALF to utilize for all assays utilized was not feasible. We are in the process of evaluating if we can assess this adequately in T cells from explanted lungs and lymph nodes.

Comment 7: The difference between + vs. – sEVs of IL4 and IL17 protein production was about 2 folds per flow-cytometry (Fig. 1E and Fig. S1B), yet the mRNA expression was about 10 folds. What accounts for such a big difference?

Response: We provide cytokine data for IL-4 and IL-17 in support of our in vitro human data (Figure 1F & 1G) and in vivo mouse data (Figure 11H & 11I). We agree with the reviewer that mRNA changes are more robust in those samples. There is variability in extent of T cell proliferation and Th polarization elicited by sEVs in autologous T cells from asthmatics. This may account for these differences.

Comment 8: Fig. 1G, GATA3 was downregulated in + sEVs samples, why Th2 was upregulated in these samples? Were Th17 specific gene signatures, including Ror, preferentially upregulated in + sEVs samples?

Response: We appreciate this comment and have provided additional data for RORC and GATA3 expression in Figure 1. RORC expression significantly changed with sEV-co-culture with T cells in asthmatics. We saw an increase in expression for GATA3, but didnt reach significance. Similar sample variability was seen in our NanoString transcriptomic data for GATA-3. But our Th2 cytokine data are supportive of Th2 differentiation (Figures 1F & 1G; 11H & 11I).

Comment 9: Fig. S3A. T cell proliferation assays as in Fig. 1D should also be performed to assess T cell activation, as the readout could be more robust.

Response: We agree that readouts of T cell activation markers have been provided as early indicators of T cell activation in sEV-T cell co-cultures. In our mechanistic studies with rotenone, we have assessed Th polarization in these experiments. These outcomes are presented in Supplementary Figure S18 of the revised manuscript.

Reviewer #3 (Remarks to the Author):

The authors propose that airway myeloid-derived regulatory cells (MDRCs) in asthma secrete small extracellular vesicles (sEVs) that transfer mitochondria-related signals to CD4 T cells, driving T cell proliferation and Th2/Th17 polarization via mitochondrial oxidant–dependent NF-κB signaling. They further argue that internalized sEV/mitochondrial signal localizes with polarized cytoskeleton/mitochondrial networks in recipient T cells, that DRP1-promoted mitochondrial dynamics contribute to mitochondrial packaging into sEVs, and that intranasal transfer of MDRC-derived sEVs exacerbates allergic airway inflammation in vivo.

The revision addresses some of my previous comments concerning repeated presentations of the same data, potential misinterpretation of co-expression of multiple markers based solely on imaging data, the lack of flow data quality control and gating, and data consolidation (some of them). The authors seem to prefer detailed (most of the time repeated) presentation of methods and similar datasets, which may be further consolidated in the overall research presentation. Currently, the manuscript appears long, which may not be necessary. For example, the asthmatic phenotyping data were spread into three main text figures. The reviewer hopes that the authors reconsider combining subfigures for the asthma experimental workflow, cell characterization, histology images (with scores) into one main figure.

Response: We appreciate the comments of the reviewer. As suggested by the reviewer, we have consolidated the asthma experimental workflow schematic, cell characterization and histology images (with scores) into one main figure which is now New Main Figure 9. The enlarged areas are now shown in Supplementary figure S19.

Beyond formatting, I have the following major questions:

1. It is not surprising to see significant roles of MDRC/sEV MHC-II and mitochondrial activity in Th2/Th17 differentiation. However, it is unlikely that these could be therapeutic targets to attenuate pathogenic T helper cells during asthma and other related diseases. Can blocking/inhibiting sEVs or sEV-specific signals attenuate asthmatic responses?

Response: We agree with the reviewer that this is a very important and interesting question. We have shown MDRC-sEV-mediated mechanisms and as a proof of principle, that sEV transfer exacerbate asthma pathology and airway inflammation. Currently, to address the reviewer’s question, there are no standard procedures to remove sEVs or reduce specific sEVs *in vivo*. The available inhibitors of sEV biogenesis and release, e.g. GW4869, inhibit sphingomyelinases nonspecifically and in all cells, and can affect the function of both beneficial and disease causing sEVs and their potential for intercellular communication. While this is a very interesting future question for us to explore with

novel genetic and transfer approaches, we feel that these are beyond the scope of the current manuscript. In the discussion of the manuscript, we have included this interesting question as a future direction. This has been included in the discussion on lines 486-490, page 21-22.

2. The authors did not test whether MHC-II blockade alters mitochondrial ROS generation/release or other sEV characteristics that are directly relevant to their roles in regulating T cells.

Response: We did not perform MHC class blocking, but instead we showed that regulation of T cell responses occurs only in co-culture with autologous sEVs and not with allogeneic sEVs. As pan-MHC blockade inhibits these responses, we consider that this experiment may be redundant.

3. Given that no viability dye was included in the flow cytometric analyses, and that the cytokine staining signals in the highly inflamed lung samples appeared dim. It is possible that some dead cells, which tend to show autofluorescence in flow cytometry, were included in the analyses and biased the conclusions. To ensure that Th2/Th17 responses are indeed enhanced in the asthma model, additional validation, such as cytokine ELISA, should be considered.

Response: We are sorry that we did not highlight the inclusion of new cytokine data in the latest submission, it was overlooked due to that this was changed in an earlier version only reviewed by the editor. We have performed ELISA for IL-4 and IL-17 both in *in vitro* supernatants and in *ex vivo* BAL fluid. Cytokine levels in co-cultures of human BALF sEVs with human T cells were presented in Figure 1. We have now highlighted these results on page 6, line 131 and in the figure legend. In our sEV transfer experiments in mice, we determined the concentrations of IL-4 and IL-17 A in BAL fluid collected from transfer recipients. These results were presented in Figure 11 of our previous submission which is now Figure 10 in the revised manuscript. We have now highlighted these results on page 18, lines 411-412 and in the figure legend.

4. The method section listed flow antibodies for Treg cells, but no Treg-related data were presented. What happened to the Treg cells?

We did not see any differences in proportions or T reg numbers in the lung tissues of the sEV transfer recipient mice.
